# Pure Molecular Inorganic Rings: Mixed Group 14/15 Metallacycles

**DOI:** 10.1002/anie.202520366

**Published:** 2025-11-22

**Authors:** Stefanie Maier, Xiaofei Sun, Lisa Zimmermann, Ralf Köppe, Manfred Scheer, Peter W. Roesky

**Affiliations:** ^1^ Karlsruhe Institute of Technology (KIT) Institute for Inorganic Chemistry Kaiserstr. 12 Karlsruhe 76131 Germany; ^2^ Institute of Inorganic Chemistry University of Regensburg Universitätsstr. 31 Regensburg 93040 Germany; ^3^ Karlsruhe Institute of Technology (KIT) Institute of Nanotechnology Kaiserstr. 12 Karlsruhe 76131 Germany

**Keywords:** Antimony, Arsenic, Heterocycles, Insertion, Phosphorus

## Abstract

A systematic study on the reactivity of the homodipnictogen tetrahedrane complexes [{Cp‘Mo(CO)_2_}_2_(μ,η^2:2^‐E_2_)] (E ═ P, As, Sb; Cp‘ = η^5^‐C_5_H_4_ *t*Bu) towards the disilylene [L^Ph^Si]_2_ and digermylene [L^Ph^Ge]_2_ (L^Ph^ = PhC(N*t*Bu)_2_) is presented. Depending on the specific combination of tetrel and pnictogen atom, a variety of novel silicon‐pnictogen and germanium‐pnictogen heterocycles in different bonding situations were obtained. This is a significant development, as these metallacycles are unprecedented and offer new possibilities for further reactivities. These three‐ to five‐membered pure inorganic heterocycles include one Mo, one or two tetrel, and one or two pnictogen atoms in each case. Especially for the heavier pnictogen Sb, this cyclization behavior forming novel three‐ and four‐membered Si–Sb as well as five‐membered Ge–Sb ring systems has not been observed so far. These results present unique examples of heavy group 14/15 heterocycles.

## Graphical abstract

The synthesis and characterization of novel mixed group 14/15 metallacycles incorporating a transition‐metal is described. All of the presented compounds were obtained by reduction of an organometallic Mo_2_E_2_ tetrahedrane using Si(I) and Ge(I) compounds, respectively. The size of the obtained metallacycles (three‐, four‐, five‐membered rings) is strongly dependent on the combination of the tetrel as well as the pnictogen atoms.

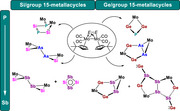

## Introduction

**Figure 1:**
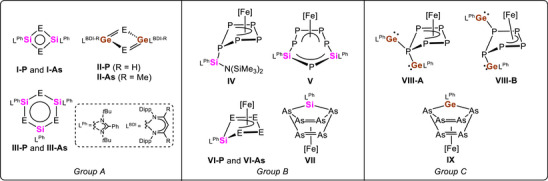
Selected examples of ring compounds based purely on main‐group elements: silaphospha‐ and silaarsa‐cyclobutadiene and benzene (*Group A*), tetrylene‐functionalized derivates of [Cp*Fe(η^5^‐E_5_)] (E ═ P, As; Cp* = C_5_Me_5_) obtained by reaction with mono‐ and disilylenes (*Group B*) as well as digermylenes (*Group C*); (L^Ph^ = PhC(N*t*Bu)_2_; L^BDI‐H^ = CH(CHNDipp)_2_; L^BDI‐Me^ = CH(CMeNDipp)_2_; Dipp = 2,6‐*i*Pr_2_C_6_H_3_; [Fe] = Cp*Fe).

In recent decades, the activation and subsequent functionalization of molecular group 15 compounds has evolved into a competitive area of research. Amongst them, organometallic transformation reactions focus on white phosphorus (P_4_)^[^ ^1^, ^2^, ^3^ ^]^ and to a minor extend on yellow arsenic (As_4_)^[^ ^4^, ^5^, ^6^, ^7^, ^8^ ^]^ with their readily available yet highly reactive tetrahedral cage structures, whereas bulk materials of grey arsenic and grey antimony mostly behave inertly in chemical reactions, with the exception of their nano‐sized forms.^[^ ^9^, ^10^, ^11^, ^12^, ^13^ ^]^ One of the most efficient and versatile main‐group compounds for pnictogen‐pnictogen bond activation reactions are silylenes with a large number of activation products known in literature which are of great scientific as well as industrial interest. In common P_4_‐activation pathways with silylenes, Si‐functionalized (poly)phosphorus cages,^[^ ^14^, ^15^, ^16^, ^17^, ^18^ ^]^ chains,^[^ ^18^, ^19^ ^]^ and fragmentation compounds^[^ ^20^, ^21^, ^22^ ^]^ have been received whereas much less reactivity is reported with the heavier As_4_. By far, only cages and other cyclic compounds were isolated.^[^ ^5^, ^7^ ^]^ Within the class of main‐group‐functionalized pnictogen compounds, special attention is placed on heterocyclic systems as they often possess unique electronic and structural properties.^[^ ^23^, ^24^, ^25^, ^26^, ^27^, ^28^, ^29^, ^30^, ^31^, ^32^ ^]^ In 2011, the groups of H. Roesky and M. Driess independently reported on a silicon‐phosphorus congener of cyclobutadiene, [(L^Ph^Si)_2_P_2_] (L^Ph^ = PhC(N*t*Bu)_2_; Figure 1: **I‐P**),^[^ ^21^, ^22^ ^]^ with a planar Si_2_P_2_ heterocycle possessing unique electronic properties. Few years later, these findings were extended towards the isostructural arsenic analogue: Using a zirconocene‐captured As_4_ cage as transfer agent provided four‐membered [(L^Ph^Si)_2_As_2_] (Figure 1: **I‐As**)^[^ ^6^ ^]^ under mild reaction conditions. Not being limited to silicon, similar results were obtained for the heavier homologue germanium by M. Driess and coworkers.^[^ ^33^, ^34^ ^]^ Starting from phospha‐ and arsaketenyl‐functionalized germylene compounds, 4π‐electron stabilized ring systems [(L^BDI‐H^Ge)_2_P_2_] (Figure 1: **II‐P**) and [(L^BDI‐Me^Ge)_2_As_2_] (L^BDI‐H^ ═ CH(CHNDipp)_2_; L^BDI‐Me^ ═ CH(CMeNDipp)_2_; Dipp = 2,6‐*i*Pr_2_C_6_H_3_; Figure 1: **II‐As**) with ylide‐like Ge–As bonds were accessed by CO‐elimination. Subsequently, larger pnictogen–silicon congener of benzene, [(L^Ph^Si)_3_E_3_] (E ═ P (Figure 1: **III‐P**), As (Figure 1: **III‐As**)),^[^ ^6^ ^]^ with electron delocalization and alternating pnictogen–silicon bonds were reported (Figure 1: *Group A*).

To extend this research towards mixed group 14/15 heterocycles, our groups successfully proved that *cyclo*‐E*_n_* (*n* = 3 ≤ n ≤ 5) ligands stabilized by transition‐metal units are excellent building blocks for functionalization reactions with different low‐valent main‐group compounds. The *cyclo*‐E_5_ ligands of the air‐stable pentaphospha‐ and pentaarsaferrocene [Cp*Fe(η^5^‐E_5_)] (E ═ P, As, Cp* = C_5_Me_5_) initially reported by Scherer^[^ ^35^, ^36^ ^]^ evolved as multifaceted polypnictogen ligands and their reactivity towards low‐valent s‐block^[^ ^37^, ^38^ ^]^ as well as p‐block^[^ ^39^, ^40^, ^41^, ^42^, ^43^, ^44^, ^45^, ^46^ ^]^ compounds has been thoroughly investigated in the past years. A large structural diversity of products was obtained using heavier group 14 species: reaction of mono‐ and disilylenes with [Cp*Fe(η^5^‐P_5_)] afforded monophosphorus functionalization compounds (Figure 1: **IV**) as well as the selective insertion of one or two silylene units in two adjacent P–P bonds of the *cyclo*‐P_5_ ligand yielding novel *cyclo*‐P_4_‐SiPSi‐ (Figure 1: **V**) and *cyclo*‐SiP_4_‐ligands (Figure 1: **VI‐P**) stabilized by a transition‐metal moiety.^[^ ^42^ ^]^ Compared to these results, reaction of the disilylene with [Cp*Fe(η^5^‐As_5_)] provided the isostructural *cyclo*‐SiAs_4_ ring (Figure 1: **VI‐As**) with an *envelope*‐conformation alongside with a norbornadiene‐like As_6_Si‐ligand (Figure 1: **VII**) in equal amounts (Figure 1: *Group B*). The isostructural complex of the latter compound was also formed when [Cp*Fe(η^5^‐As_5_)] and the heavier digermylene were reacted (Figure 1: **IX**).^[^ ^46^ ^]^ No alteration of the *cyclo*‐P_5_ ligand itself, but a di‐functionalization accompanied with subsequent 1,2‐migration of one germylene unit yielding the isomers **VIII‐A** and **VIII‐B** was observed in the reaction of the Ge(I) species with [Cp*Fe(η^5^‐P_5_)] (Figure 1, *Group C*).^[^ ^40^ ^]^ It is important to note that the transition‐metal atoms are not included in any of these inorganic cycles, they are solely exocyclic π‐coordinated (see Fe‐atoms, Figure 1: **IV** to **IX**). Recently, R. Wolf and coworkers expanded the scope of mixed group 14/15 heterocycles, proving that heavier group 14/15 cyclopentadienyl anions [SnP_4_]^2−^ and [PbP_4_]^2−^ as well as *envelope*‐[P_4_Sn] ring structures are also accessible from white phosphorus using heterometallic compounds.^[^ ^47^, ^48^ ^]^ Additionally, mixed group 14/15 heterocycles can also be obtained by salt metathesis reactions using mono‐reduced [CoE_3_] (E ═ P, As)^[^ ^49^ ^]^ as well as [CoP_4_]^[^ ^50^ ^]^ complexes as starting materials. In view of this state‐of‐the‐art the question arose, if a synthetic pathway can be established in which an access to novel chain, but especially ring structures can be achieved in which also a transition metal is incorporated into such systems.

**Scheme 1:**
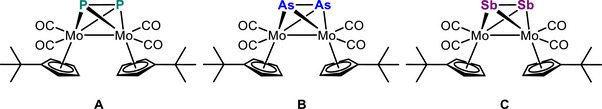
Homodipnictogen complexes [{Cp‘Mo(CO)_2_}_2_(μ,η^2:2^‐E_2_)] (E ═ P (A),^[^ ^51^ ^]^ As (B),^[^ ^52^, ^53^ ^]^ Sb (C)^[^ ^53^, ^54^ ^]^; Cp‘ = η^5^‐C_5_H_4_ *t*Bu).

Herein, we report our findings on novel group 14/15 metallacycles, which are a new class of compounds that feature the incorporation of a transition‐metal into their ring structures. We showcase the functionalization of one of the simplest organometallic *E*_n_ ligand compounds, the homodipnictogen complexes [{Cp‘Mo(CO)_2_}_2_(μ,η^2:2^‐E_2_)] (Scheme 1: E ═ P (**A**),^[^ ^51^ ^]^ As (**B**),^[^ ^52^, ^53^ ^]^ Sb (**C**)^[^ ^53^, ^54^ ^]^; Cp‘ = η^5^‐C_5_H_4_ *t*Bu) with ditetrylenes [L^Ph^Si]_2_^[^ ^55^ ^]^ and [L^Ph^Ge]_2_^[^ ^56^ ^]^ (L^Ph^ ═ PhC(N*t*Bu)_2_). From these reactions, a variety of unprecedented three‐ to five‐membered mixed group 14/15 metallacycles were obtained representing a variety of structural motifs and electronic properties.

## Results and Discussion

### Reduction Chemistry with Si(I) Compound [L^Ph^Si]_2_

**Scheme 2:**
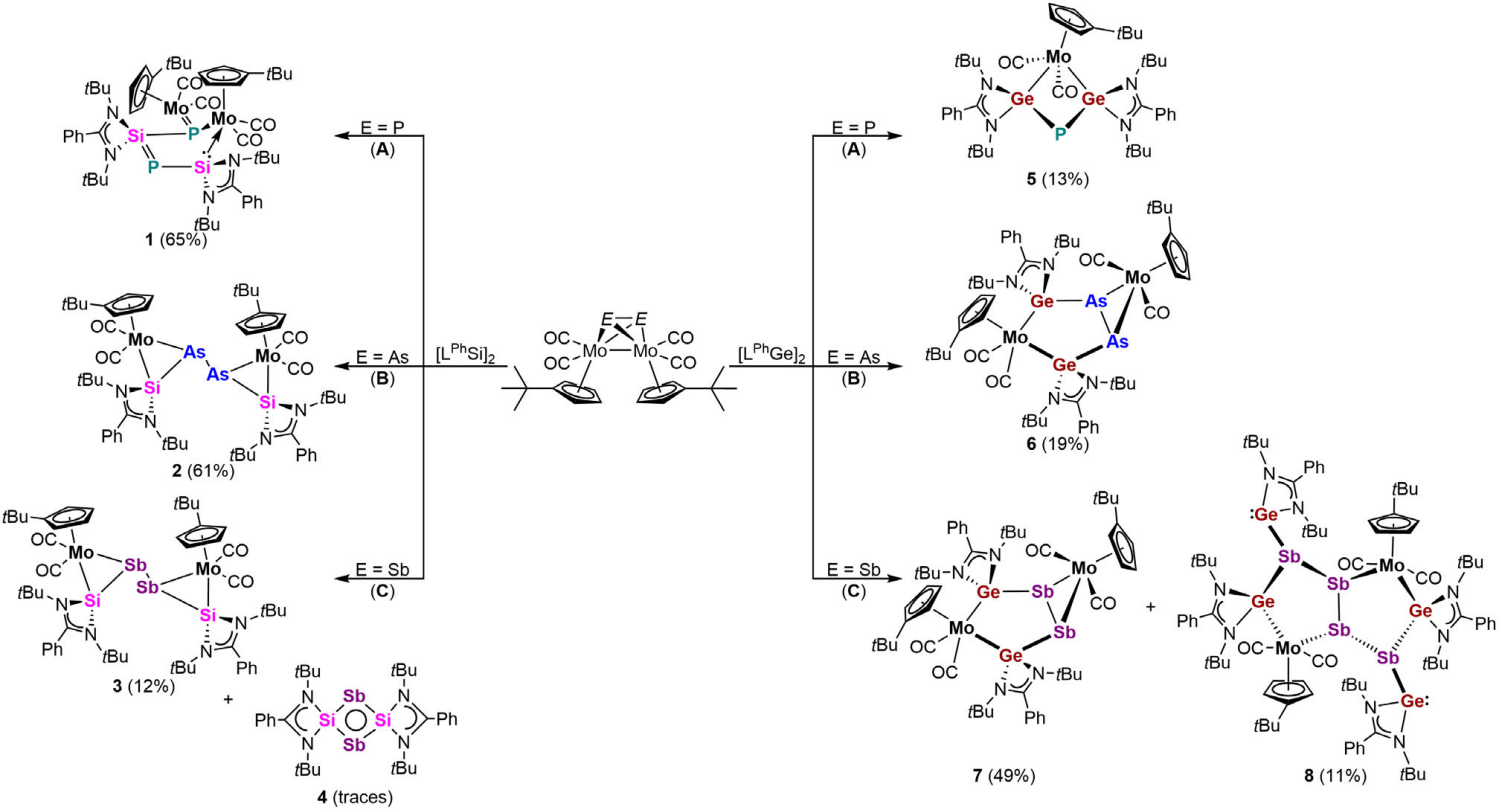
Reactivity of [{Cp‘Mo(CO)_2_}_2_(μ,η^2:2^‐E_2_)] (E ═ P (A), As (B), Sb (C); Cp‘ = η^5^‐C_5_H_4_ *t*Bu) towards interconnected disilylene [L^Ph^Si]_2_ (left) and digermylene [L^Ph^Ge]_2_ (right, L^Ph^ = PhC(N*t*Bu)_2_). Yields of single crystals are given in parenthesis.

**Figure 2:**
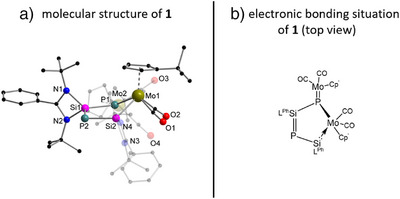
Left side: Molecular structure of **1** in the solid state. Hydrogen atoms are omitted for clarity. Selected bond distances [Å] and bond angles [°]: Mo1–P1 2.5412(12), Mo1–Si2 2.519(2), Mo2–P1 2.324(2), P1–Si1 2.283(2), P2–Si1 2.123(2), P2–Si2 2.199(2); Si2–Mo1–P1 76.84(5), Mo2–P1–Mo1 129.63(5), Si1–P1–Mo1 110.26(6), Si1–P1–Mo2 119.19(6), Si1–P2–Si2 92.53(7), P2–Si1–P1 113.78(7), P2–Si2–Mo1 126.58(6). Right side: Electronic bonding situation in **1** supported by determined force constants (Raman spectroscopy).

Reaction of [L^Ph^Si]_2_^[^ ^55^ ^]^ with the molybdenum diphosphorus complex [{Cp‘Mo(CO)_2_}_2_(μ,η^2:2^‐P_2_)] (**A**) at room temperature resulted in the highly selective formation of **1** as a new product (Scheme 2) along with traces of the literature‐known **I‐P** (Figure 1).^[^ ^21^, ^22^ ^]^ The latter compound can be identified as a singlet at −164.9 ppm when the reaction is monitored by ^31^P{^1^H} NMR spectroscopy. Interestingly, due to the high reactivity of [L^Ph^Si]_2_, the Mo–Mo bond was broken during this reaction which is a rather rare conversion mode for these species.^[^ ^57^, ^58^, ^59^ ^]^ Initially, small amounts of yellow crystals of **I‐P** were precipitated and removed from the reaction mixture. Subsequently, dark‐violet crystals of **1** were obtained by slow evaporation of the toluene mother liquor. Single crystal X‐ray diffraction (SCXRD) analysis of **1** (Figure 2) reveals a five‐membered MoSiP heterocycle as central core structure featuring an *envelope*‐geometry. Both silicon and phosphorus atoms are assembled in‐plane while the ring‐molybdenum atom with its Cp‘ and carbonyl ligands is lifted out of plane by 110.26(6)° towards the P1 and 126.58(6)° towards the Si2 atom, respectively. The Si1–P2 (2.123(2) Å) and Si2–P2 (2.199(2) Å) distances are in the respective double (∼2.09 Å)^[^ ^60^ ^]^ and single (∼2.25 Å)^[^ ^61^ ^]^ bond range and are comparable with Si–P distances in the *cyclo*‐P_4_‐SiPSi ligand in **V** (Figure 1) (for structural validation see discussion of Raman spectra vide infra).^[^ ^42^ ^]^ The Si1–P1 (2.283(2) Å) as well as the Mo1–P1 (2.5412(12) Å) and Mo1–Si2 (2.519(2) Å) bond lengths are slightly longer than their corresponding single bonds.^[^ ^61^ ^]^ Compound **1** also shows the coordinative versatility of the molybdenum unit as both transition‐metal atoms are coordinated differently which has been observed in previous reactions performed by our group:^[^ ^62^ ^]^ Mo1 within the heteronuclear ring system has a square‐pyramidal geometry, while the exocyclic Mo2 atom is only coordinated by four ligands in a piano‐stool coordination environment. The Mo2–P1 distance of 2.324(2) Å also hints towards having double bond character as it is slightly shorter than the theoretically expected single bonds (∼2.49 Å vs.∼2.25 Å, for structural validation see discussion of Raman spectra vide infra).^[^ ^60^, ^61^ ^]^ Compound **1** is very well soluble in common polar and non‐polar solvents, but readily decomposes in chlorinated solvents (CH_2_Cl_2_, CHCl_3_) accompanied with immediate color change from deep violet to orange and yellow, respectively. The ^31^P{^1^H} NMR spectrum of **1** reveals a broad singlet at 555.2 ppm for the di‐molybdenum coordinated phosphorus nuclei^[^ ^63^ ^]^ and a doublet at −148.8 ppm (^2^ *J*_PP_ = 56 Hz) corresponding to the di‐coordinated phosphorus atom with the former being strongly downfield‐shifted by ca. 704 ppm compared to the latter signal. The ^29^Si{^1^H} NMR spectrum shows a doublet at 126.5 ppm (^1^ *J*_SiP_ = 113 Hz) and a multiplet resonance at 58.0 ppm. Interestingly, the ^1^H NMR spectrum of **1** indicates a dynamic behavior in solution. Only very broad resonances are seen at room temperature but cooling a toluene‐*d*_8_ solution of **1** to 273 K and below led to a well‐defined sharp pattern (Figure S3).

**Figure 3:**
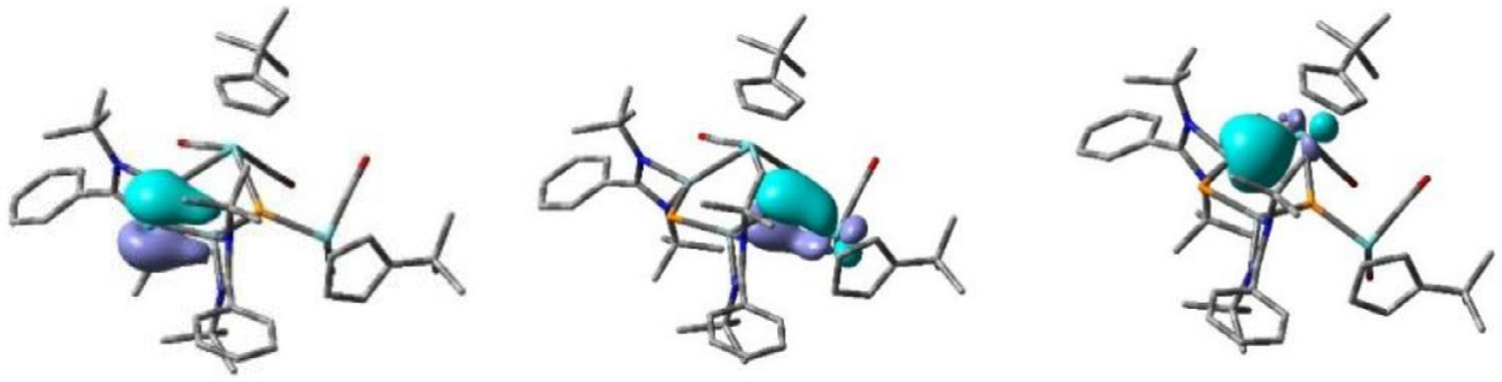
Isosurface plots (values ± 0.06 e) of the localized MOs describing the Si–P and Mo–P π‐bonds (LMOs 265 and 262) as well as the dative Si–Mo bond (LMO 233) of **1**.

To evaluate the bonding properties in **1**, vibrational investigations using Raman spectroscopy were carried out. For additional validation, the results were compared with those obtained by quantum chemical calculations (for full discussion of data cf. Supporting Information). The thereby obtained local force constants nicely describe the situation of the potential curve close to the equilibrium distance between two atoms and thus are particularly suitable for estimating molecular bond strengths.^[^ ^64^ ^]^ On the basis of these theoretical calculations (see Table S2) a local force constant of 1.194 mdyn Å^−1^ for the Mo1–P1 bond within the heterocycle and a value of 2.189 mdyn Å^−1^ for the *exo*‐Mo2–P1 bond are determined. Since, to our knowledge, no Mo–P force constants are known in the literature, the value in [Mo(CO)_5_PMe_3_] as an example of a typical Mo–P single bond was calculated for comparison (f(Mo–P) = 1.155 mdyn Å^−1^, see Supporting Information). From these findings, we conclude that the bond between Mo2 and P1 is best described as double bond, which is also supported by the rather divergent bond lengths of P1 towards both Mo atoms (P1–Mo1 2.5412(12) Å and P1–Mo2 2.324(2) Å). Significant differences are also observed within the derived local force constant values of the three Si–P bonds: The smallest value is 1.53 (Si1–P1) followed by 1.84 (Si2–P2) and 2.42 mdyn Å^−1^ (Si1–P2) with the latter local force constant being in line with a double bond for Si1–P2 (Figure 2, right). Additionally, the isosurface plots of the localized molecular orbitals (MOs) describing the Si–P and Mo–P π‐bonds as well as of the Si–Mo dative bond are depicted in Figure 3.

**Figure 4:**
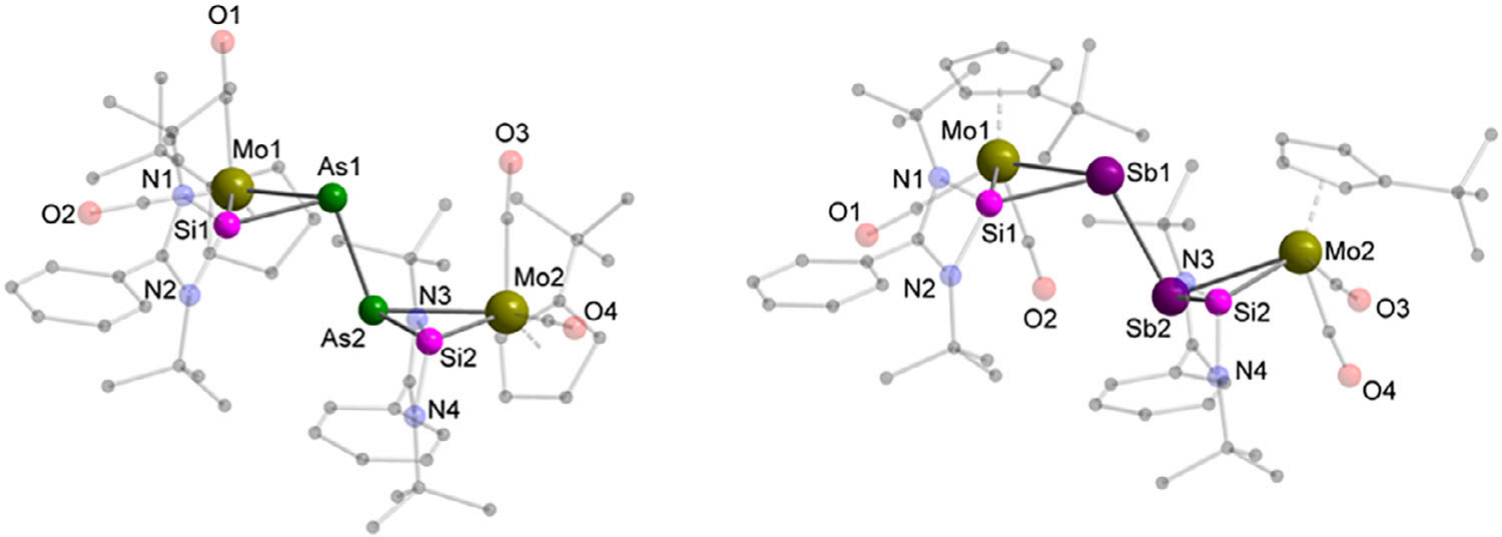
Molecular structure of **2** (left) and **3** (right) in the solid state. Hydrogen atoms and non‐coordinating solvent molecules are omitted for clarity. Selected bond distances [Å]: For **2**: Mo1–As1 2.7528(3), Mo1–Si1 2.4039(6), Mo2–As2 2.7351(3), Mo2–Si2 2.3943(6), As1–As2 2.4905(3), As1–Si1 2.2677(6), As2–Si2 2.2602(6); For **3**: Si1–Sb1 2.4621(7), Sb1–Mo1 2.9077(4), Mo1–Si1 2.4138(7), Sb1–Sb2 2.8456(4), Si2–Sb2 2.4763(7), Sb2–Mo2 2.9306(4), Mo2–Si2 2.4195(7).

Compared to the selective formation of five‐membered heterocycle **1** from diphosphorus complex **A**, reaction of [{Cp‘Mo(CO)_2_}_2_(μ,η^2:2^‐As_2_)] (**B**) (Scheme 2) with Si(I) species [L^Ph^Si]_2_ yielded orange crystals of the diarsine **2** as SCXRD analysis revealed (Figure 4, left). Upon reaction with [L^Ph^Si]_2_, the Mo–Mo bond and one of the two Mo–As bonds at each arsenic atom of **B** are cleaved while the As–As single bond remains intact. The resulting diarsine (As_2_) core adopts a *trans*‐bent geometry caused by one lone pair of electrons at each arsenic atom.^[^ ^65^ ^]^ Its As–As single bond distance of 2.4905(3) Å is significantly longer than in the precursor **B** (2.312 Å).^[^ ^53^ ^]^ Both pnictogen atoms are bonded towards one Mo as well as one Si unit forming a purely inorganic MoAsSi three‐membered ring system which is only the second example of a three‐membered As/group 14 metallacycle bearing a transition‐metal atom after the seminal reports of a NiAsSi triangle by T. Hadlington and M. Driess.^[^ ^66^ ^]^ The observed Mo–As distances are with a mean value of 2.745 Å considerably elongated compared to the expected single bonds (∼2.59 Å),^[^ ^61^ ^]^ whereas the Mo–Si and As–Si bond lengths are ca. 0.1 Å shorter (mean values: 2.399 versus ∼2.54 Å and 2.264 versus ∼2.37 Å).^[^ ^61^ ^]^ All three single bonds within both MoAsSi triangles are of comparable sizes. The MoAsSi‐heterocycles in **2** are stabilized by the respective organometallic ligands and represent the first example of a diarsenic compound connecting two mixed transition‐metal/group 14 rings by the pnictogen atom. Compound **2** is virtually insoluble in common organic solvents such as THF, toluene and diethylether as well as hydrocarbons. Only in more polar solvents like chlorinated solvents (CHCl_3_, CH_2_Cl_2_) it dissolves with decomposition after few minutes (cf. Supporting Information). In the ^29^Si{^1^H} NMR spectrum, a singlet at 118.4 ppm for the Si atoms in **2** is observed.

When the heavier complex [{Cp‘Mo(CO)_2_}_2_(μ,η^2:2^‐Sb_2_)] (**C**) was reacted with the disilylene [L^Ph^Si]_2_, the distibine species **3**, which is isostructural to **2** was obtained as red, plate‐shaped crystals from a C_6_D_6_ solution (Scheme 2). However, the formation of byproducts, which could not be isolated was seen in the NMR spectra. SCXRD analysis unequivocally disclosed the molecular structure of the distibine **3** with two MoSbSi‐triangles connected to each other by their pnictogen atoms (Figure 4, right). Both Sb atoms of the non‐centrosymmetric distibine (Sb_2_) unit are coordinated by one Mo and one tetrel atom. The resulting *trans*‐bent orientation along the distibine is characteristic for heavier main‐group elements due to the presence of a lone pair of electrons at each of the Sb atoms.^[^ ^65^ ^]^ The Sb1–Sb2 bond distance of 2.8456(4) Å lies in the range of typical Sb–Sb single bond lengths found in distibines R_4_Sb_2_ (2.64–3.03 Å)^[^ ^67^ ^]^ and is in good agreement with the bis(stibahousene) by V. Lee et. al. having a nearly identical Sb–Sb bond distance of 2.8773(4) Å.^[^ ^68^ ^]^ The bond distances and angles within both MoSbSi heterocycles are comparable and show the same tendencies of elongated Mo–pnictogen (2.919 versus ∼2.78 Å)^[^ ^61^ ^]^ as well as shortened Sb–Si (2.469 versus ∼2.56 Å)^[^ ^61^ ^]^ and Mo–Si (2.417 versus ∼2.54 Å)^[^ ^61^ ^]^ distances as found in **2**. In contrast, the fold‐angles along the Sb_2_‐unit differ significantly by approximately 12° (Si1–Sb1–Sb2 versus Si2–Sb2–Sb1) and 14° (Mo1–Sb1–Sb2 versus Mo2–Sb2–Sb1). Compound **3** shows a similar insolubility in organic solvents like THF, toluene, and hydrocarbons as its lighter congener **2**. In CH_2_Cl_2_ crystals of **3** dissolve under decomposition within seconds accompanied by a change of color from dark orange to yellow, preventing any characterization by ^13^C{^1^H} and ^29^Si{^1^H} NMR spectroscopy. To the best of our knowledge, both complexes **2** and **3** represent the first isostructural diarsine and distibine compounds with interconnected inorganic three‐membered ring systems at both pnictogen atoms and the first mixed transition‐metal silastibine heterocycle reported up to date.

Vibrational spectroscopic investigations of the isostructural compounds **2** and **3** were conducted using Raman spectroscopy and accompanied by computational density functional theory (DFT) calculations (cf. Supporting Information, section IV). In these molecules the pnictogen–pnictogen bonds are of particular interest. For **2**, the theoretical local force constant value of the inter‐pnictogen bond amounts to 0.94 mdyn Å^−1^ and is thus in good agreement with a weak As–As single bond compared to that in yellow arsenic (As_4_, 1.674 mdyn Å^−1^).^[^ ^69^ ^]^ A comparable situation is found for the Sb–Sb bond in distibine **3**. A local mode force constant value of 0.779 mdyn Å^−1^ is found, which is also in agreement with a weak Sb–Sb single bond after comparison to the single bond in the theoretically isostructural antimony tetrahedrane (Sb_4_, 1.224 mdyn Å^−1^, for detailed discussion cf. Supporting Information, section IV).^[^ ^69^ ^]^ The occurrence of the five‐membered metallacycle in the case of phosphorus in contrast to the smaller three‐membered heterocycles observed in the heavier homologues arsenic and antimony can hardly be explained by a single reason, but the thermochemical results are in line with the experimental findings: The calculations confirm the thermodynamic stability of the experimental structures of **1** (68 kJ mol^−1^ more stable than the hypothetical three‐membered P ring structure) as well as of **3** (33 kJ mol^−1^ more stable than the analogous five‐membered ring compound). In contrast, the energy difference in the case of arsenic is quite small and theoretically determined to be 14 kJ mol^−1^ more favorable to the five‐membered ring compound, whereas the slightly less stable three‐membered ring with an As–As single bond is found experimentally in the solid state.

**Figure 5:**
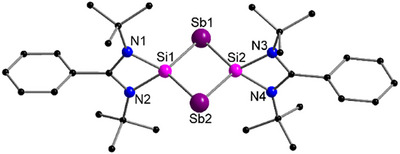
Molecular structure of **4** in the solid state. Hydrogen atoms are omitted for clarity. Selected bond distances [Å] and angles [°]: Sb1 − Si1 2.502(4), Sb1–Si2 2.496(4), Sb2–Si1 2.502(4), Sb2–Si2 2.503(3); Si2–Sb1–Si1 71.05(11), Si1–Sb2–Si2 70.95(11), Sb2–Si1–Sb1 108.90(13), Sb1–Si2–Sb2 109.08(13).

While upon the reaction of [{Cp‘Mo(CO)_2_}_2_(μ,η^2:2^‐P_2_)] (**A)** with [L^Ph^Si]_2_ compound [(L^Ph^Si)_2_P_2_] (**I‐P**, Figure 1)^[^ ^21^, ^22^ ^]^ was formed as side product, the isostructural four‐membered ring [(L^Ph^Si_2_)Sb_2_] (**4**) was isolated as a by‐product in small amounts within the same reaction leading to **3** (treatment of [{Cp‘Mo(CO)_2_}_2_(μ,η^2:2^‐Sb_2_)] (**C**) with [L^Ph^Si]_2_). Compound **4** was formed as red blocks alongside the red plates of **3** in a concentrated C_6_D_6_ solution (Scheme 2). SCXRD analysis revealed the structure of the previously unknown **4** as the silicon–antimony congener of cyclobutadiene. The formation of **4** was not surprising as the reaction of **A** with disilylene [L^Ph^Si]_2_ also verifiably led to the formation of small amounts of the four‐membered ring compound **I‐P** (Figure 1).^[^ ^21^, ^22^ ^]^ Compound **4** resembles a planar Si_2_Sb_2_ core structure with both di‐coordinated (‘naked’) antimony atoms adopting a trigonal‐planar geometry when the lone pairs of electrons are considered (Figure 5). The Si–Sb bond lengths with a mean value of 2.502 Å are in between expected Si–Sb double (2.40 Å)^[^ ^60^ ^]^ and Si–Sb single bonds (2.56 Å),^[^ ^61^ ^]^ strongly indicating a delocalization of electrons within the four‐membered ring, making compound **4** as 2,4‐disila‐1,3‐distibacyclobuta‐1,3‐diene the heaviest silicon‐pnictogen analogue of cyclobutadiene up to date. The Sb–Si–Sb bond angles, with an average value of 109.0° are clearly wider in comparison to its lighter phosphorus and arsenic analogue (**I‐P** ^[^ ^21^, ^22^ ^]^: 107.56°; **I‐As** ^[^ ^6^ ^]^: 108.61°). The interatomic Si⋅⋅⋅Si separation of 2.9045(1) Å clearly rules out any bonding interaction between both silicon atoms. Since **4** was only formed in very tiny amounts and co‐crystallization with complex **3** always occurred, full characterization via NMR, and IR spectroscopy was not possible.

To investigate the electronic properties within the Sb_2_Si_2_ ring of **4** calculations of the nucleus independent chemical shift (NICS)^[^ ^70^ ^]^ were derived by DFT calculations. To provide a better comparability, calculations were also performed for the isostructural compounds [(L^Ph^Si)_2_P_2_] (**I‐P**)^[^ ^21^, ^22^ ^]^ and [(L^Ph^Si)_2_As_2_] (**I‐As**).^[^ ^6^ ^]^ The results fit well with the reported NICS values (Table S3). For the heaviest cyclobutadiene analogue **4** negative values [NICS(1) = −2.34 and NICS(0) = −5.77] are obtained that are in a comparable range to the other two species **I‐P** and **I‐As**. Therefore, the formally expected antiaromatic behavior can be ruled out. Instead, the bonding situation in the Si_2_Sb_2_ four‐membered system within **4** is best described to be zwitterionic. This is in line with the Mulliken atomic charges of Si and the respective pnictogen atom (cf. Table S3). To evaluate the bond strength of the Si–pnictogen bond in the newly obtained compound **4** as well as its lighter homologues **I‐P** and **I‐As**, their local Si–pnictogen force constants were calculated and compared with the experimentally determined force constants of the compounds [E(SiH_3_)_3_] (for *E* = P: 1.85, As: 1.67, Sb: 1.40 mdyn Å^−1^).^[^ ^71^ ^]^ It turns out that for all three compounds the force constants expected for these strongly ionic Si–pnictogen bonds are only slightly enhanced compared to the bonds in [E(SiH_3_)_3_]. This nicely fits to the findings by H. Roesky et. al.^[^ ^21^ ^]^

### Reduction Chemistry with Ge(I) Compound [L^Ph^Ge]_2_

**Figure 6:**
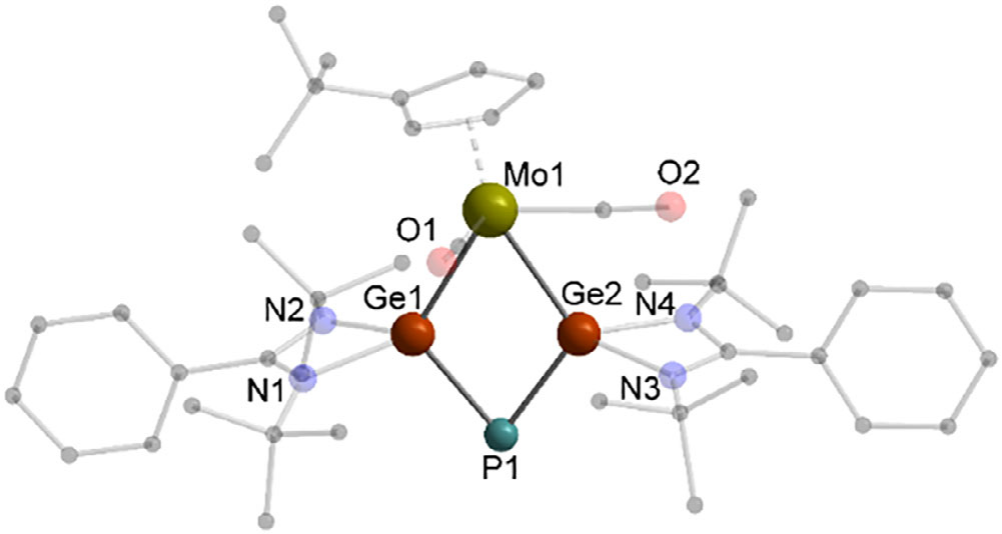
Molecular structure of **5** in the solid state. Hydrogen atoms and non‐coordinating solvent molecules are omitted for clarity. Selected bond distances [Å] and angles [°]: Ge1–Mo1 2.6132(10), Ge1–P1 2.2453(13), Ge2–Mo1 2.5954(8); Ge2–P1 2.2368(14); P1–Ge1–Mo1 110.97(4), P1–Ge2–Mo1 111.90(4), Ge2–Mo1–Ge1 62.71(2), Ge2–P1–Ge1 74.41(4).

Next, we tested the reactivity of the dipnictogen compounds **A**, **B**, and **C** towards the dimeric germylene [L^Ph^Ge]_2_ (Scheme 2, right). ^31^P{^1^H} NMR spectroscopic investigations of the reaction mixture of **A** and [L^Ph^Ge]_2_ show the slow formation of an intermediate **5′** with two doublets at 54.4 and −203.3 ppm having coupling constants of 198 Hz. However, a complete consumption of **A** by [L^Ph^Ge]_2_ was not achieved, regardless of the used equivalents of [L^Ph^Ge]_2_. Instead, the ^31^P{^1^H} signals of the initial species **5′** disappeared after some time and the formation of a new singlet at −44.0 ppm belonging to the final reaction product **5** arose with almost complete transformation at room temperature after nine days. Due to the preequilibrium to **5′** the final product was isolated in low yields. SCXRD analysis of the isolated orange crystals obtained from a concentrated C_6_D_6_ solution disclosed the formation of **5** containing a planar, diamond‐shaped MoGe_2_P‐ring as core motif (Figure 6). Both Ge atoms adopt a distorted tetrahedral geometry enclosing the ‘naked’ di‐coordinated P atom. The Ge–P bond lengths between 2.2453(13) and 2.2368(14) Å in **5** are slightly shorter than the respective distances in the *β*‐diketiminate stabilized dimer **II‐P**.^[^ ^34^ ^]^ As the Ge─P bond distances in **5** fall within the range of the respective double and single bonds,^[^ ^60^, ^61^ ^]^ a partial double bond character due to delocalization of electrons within the GePGe subunit is suggested. The molybdenum atom within the four‐membered heterocycle bridges both amidinato‐coordinated Ge units and adopts a distorted square‐pyramidal coordination environment. As the formation of **5** monitored by ^31^P{^1^H} NMR spectroscopy was unambiguously accompanied with the consumption of the intermediate **5′** the isolated compound **5** is most likely a decomposition product of **5′** by loss of one Mo unit and one phosphorus atom. Unfortunately, neither the intermediate **5′**, nor a possible decomposition species being determined in the ^31^P{^1^H} NMR spectrum could be isolated.

**Figure 7:**
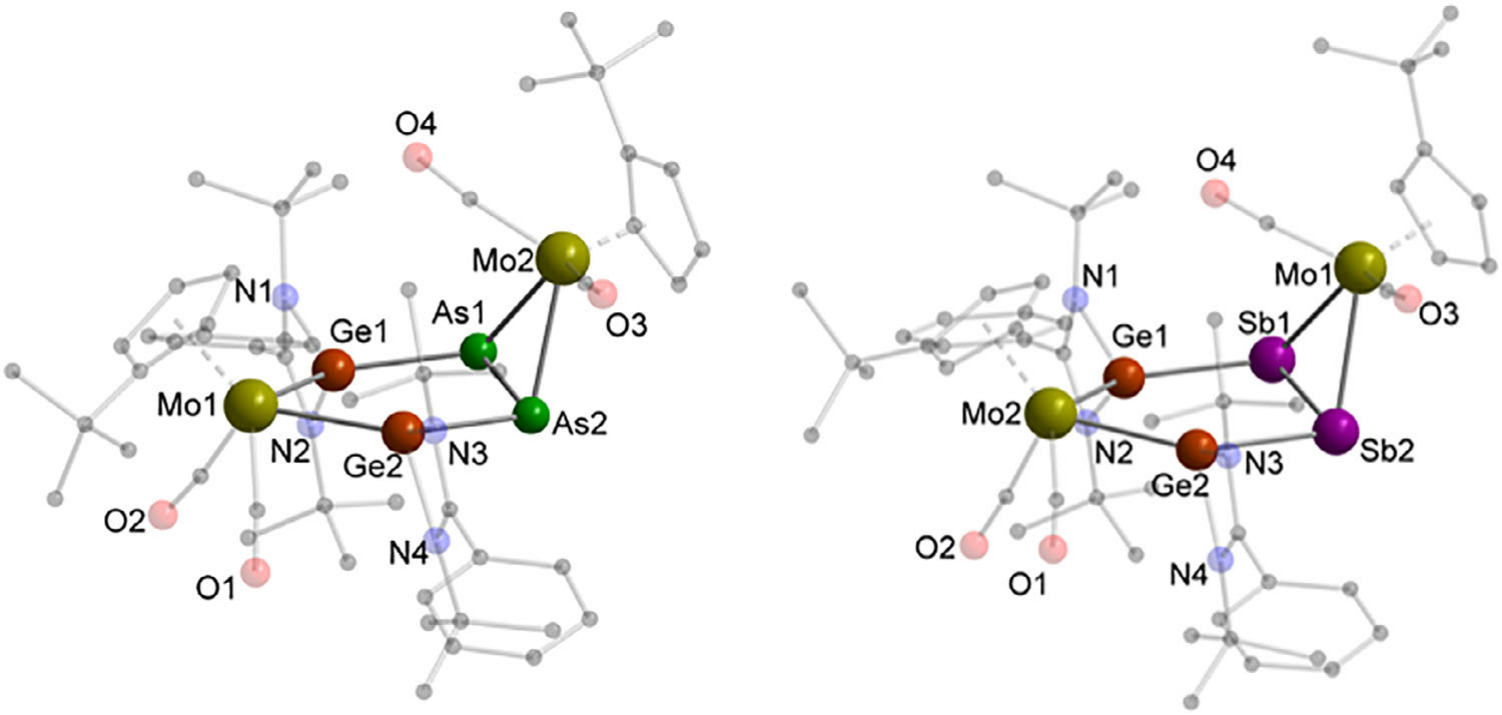
Molecular structure of **6** (left) and **7** (right) in the solid state. Hydrogen atoms are omitted for clarity. Selected bond distances [Å]: For **6**: As1–As2 2.3478(9), As1–Ge1 2.4350(9), As1–Mo2 2.6542(8), As2–Ge2 2.4752(9), As2–Mo2 2.6457(8), Ge1–Mo1 2.6078(8), Ge2–Mo1 2.6306(8); For **7**: Sb1–Sb2 2.7090(6), Sb1–Mo1 2.8265(6), Sb1–Ge1 2.6271(6), Sb2–Mo1 2.8495(6), Sb2–Ge2 2.6875(6), Mo2–Ge1 2.6242(6), Mo2–Ge2 2.6208(7).

The reaction of diarsenic complex **B** with digermylene [L^Ph^Ge]_2_ yielded red crystals of compound **6** (Scheme 2) with its molecular structure being determined by SCXRD analysis (Figure 7, left). NMR investigations show that other side products are formed, which could not be isolated. In this transformation, the Mo–Mo bond of **B** is cleaved, accompanied by the insertion of one amidinato‐substituted Ge fragment in each of the Mo–As single bonds. The core of **6** consists of two fused inorganic MoGe_2_As_2_ and MoAs_2_ rings which are sharing one As–As single bond forming a hetero‐bicyclo[3.1.0]hexane scaffold. The resulting bicyclic diarsine compound features an As–As single bond of 2.3478 Å being almost 0.15 Å shorter than the respective single bond in the linear diarsine **2**. The isosceles MoAs_2_ triangle is flipped out of plane of the five‐membered MoGe_2_As_2_ heterocycle with a dihedral angle of 69.85°, respectively and shows equal bond angles and single bond distances on both sides of the isosceles triangle. Within the inorganic MoGe_2_As_2_ ring system, the almost equal Ge–As and Ge–Mo distances match well with expected single bonds (∼2.42 and ∼2.59 Å)^[^ ^61^ ^]^ obtaining average values between 2.4551 and 2.619 Å, respectively. Considering the slightly shortened single bond between the pnictogen atoms, a delocalization of electrons within the GeAs_2_Ge unit can be clearly ruled out. To evaluate the As–As bond strength in **6** by means of theoretical methods, the local force constants and the Wiberg Bond Indices (WBI) from a Natural Bond Orbital (NBO) population analysis were determined and compared to that of As_4_ as well as of **2** (cf. Table S4). The As–As force constant in **6** is almost identical to that in As_4_, thus confirming its single‐bond character. However, the one in **2** is significantly weaker compared to a single bond. Values of the WBI confirm our findings.

Reacting the heavier complex [{Cp‘Mo(CO)_2_}_2_(μ,η^2:2^‐Sb_2_)] (**C**) with digermylene [L^Ph^Ge]_2_ lead to the formation of the hetero‐bicyclo[3.1.0]hexane distibine **7** (Scheme 2) in the form of red, rod‐shaped crystals obtained from a THF/*n*‐hexane mixture (Figure 7, right). SCXRD analysis disclosed the structure of **7**, which features an inorganic bicyclic structure and is isostructural to the diarsine **6** (Figure 7, left). The bicycle consists of a MoGe_2_Sb_2_ and a MoSb_2_ ring sharing a Sb–Sb bond with a length of 2.7090(6) Å, slightly elongated in comparison to expected single bonds distances (∼2.61 Å).^[^ ^61^ ^]^ As within the bicyclic diarsine **6**, the MoSb_2_ triangle adopts a dihedral angle of 71.975° relative to the MoGe_2_Sb_2_ ring system and has equal bond distances between the transition‐metal and the pnictogen atom with 2.838 Å in average. Considering the inorganic five‐membered MoGe_2_Sb_2_ ring, both Ge–Sb bond distances of 2.6573 Å are in good agreement with theoretical single bonds, excluding any electron delocalization within the GeSb_2_Ge unit. Additionally, the Ge–Mo bond lengths are in good agreement with the respective distances in the isostructural diarsine compound **6** having a mean value of 2.623 Å.

**Figure 8:**
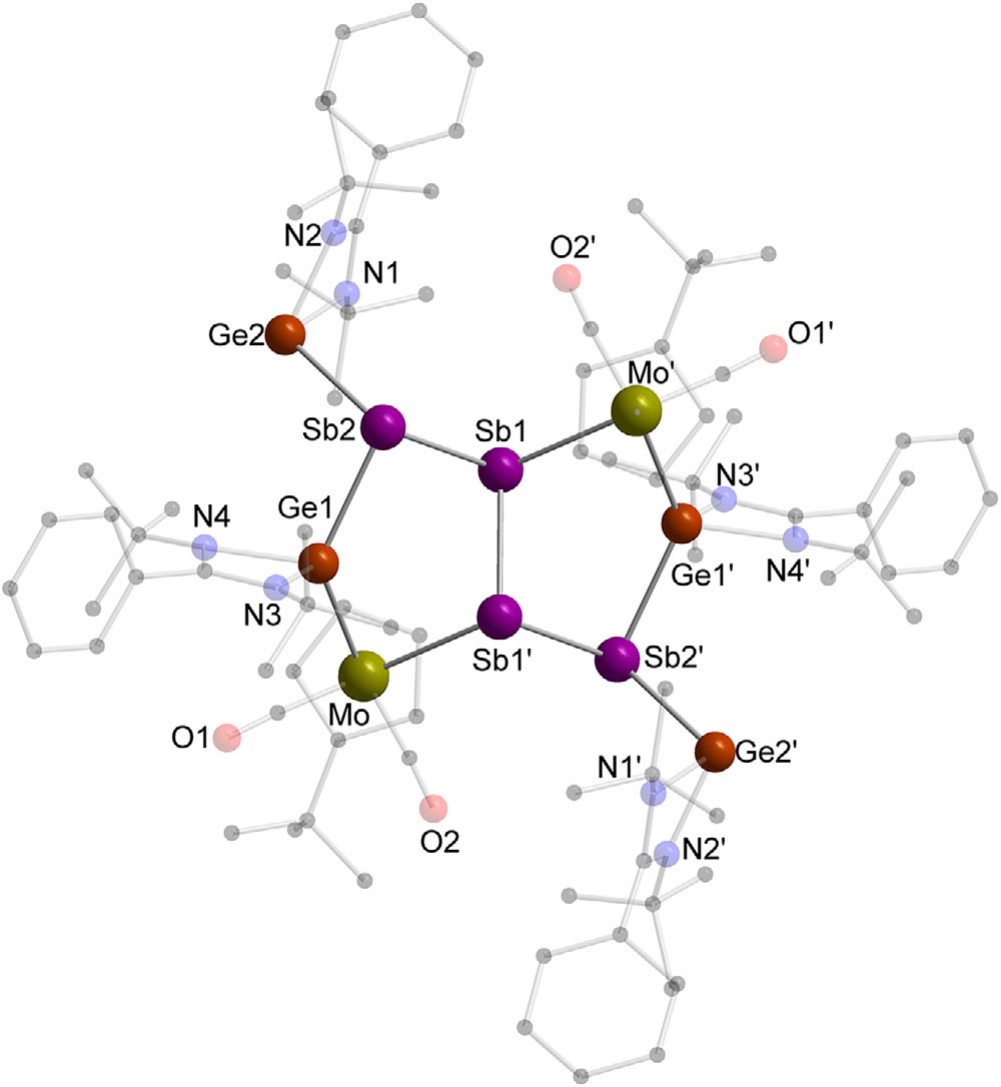
Molecular structure of **8** in the solid state. Hydrogen atoms and non‐coordinating solvent molecules are omitted for clarity. Selected bond distances [Å] and angles [°]: Sb1–Sb2 2.8252(7), Sb1–Sb1‘ 2.8196(14), Sb1‘–Mo 2.8773(10), Sb2–Ge2 2.7335(8), Sb2–Ge1 2.664(2), Mo–Ge1 2.5746(13), Ge1–N4 2.000(2), Ge1–N3 2.018(2), Ge2–N1 2.012(2), Ge2–N2 1.998(2); Sb1‘–Sb1–Sb2 88.42(3), Sb1–Sb1‘–Mo 98.65(4), Sb2–Sb1–Mo‘ 103.05(2), Ge1–Sb2–Sb1 88.31(3), Ge1–Sb2–Ge2 100.41(4), Ge2–Sb2–Sb1 106.62(2), Ge1–Mo–Sb1‘ 74.28(3), Mo–Ge1–Sb2 130.00(3).

Within the reaction of **C** with [L^Ph^Ge]_2_, red crystals of a second reaction product were obtained by slow evaporation of the solvent from a THF‐solution (Scheme 2). The molecular structure of the bicyclic pentalene‐type heteroatomic system **8** with two fused MoGeSb_3_ cycles (Figure 8) was deduced by SCXRD analysis. The main structural feature of **8** is a *trans*‐configurated Sb_4_‐chain with the inner Sb–Sb bond being shared by both five‐membered MoGeSb_3_ heterocycles. The almost identical Sb–Sb bond distances of 2.8196(14) Å (inner bond) and 2.8252(7) Å (outer bond) are in the usual Sb–Sb single bond range and comparable to compounds featuring a *cyclo*‐Sb_4_ core.^[^ ^72^ ^]^ Both Sb_4_‐chain ends are each bound towards two Ge moieties with one major difference: Ge1 inside the heterocycle adopts a tetravalent state, being connected to one Sb and one Mo atom closing the MoGeSb_3_ metallacycle. In contrast, the exocyclic Ge2 fragment is only coordinated by its amidinato ligand and the Sb2 atom, with the respective lone pair still being located at the germanium atom, thereby retaining its germylene character. The N–Ge2–Sb2 bond angles range from 100.26(7)° to 105.47(6)° and are clearly widened up compared to other germylenes. The Ge1–Sb2 bond length of 2.664(2) Å is similar to those in other digermanium substituted antimony compounds with the germanium atoms in a tetravalent state,^[^ ^73^ ^]^ while the distance between the exocyclic germylene atom and the Sb atom represents a slightly elongated single bond with 2.7335(8) Å.^[^ ^61^ ^]^ Germylene units being present in polyphosphorus systems have been reported by our groups in the past,^[^ ^40^ ^]^ but have not been observed for the heavier pnictogen homologue, making compound **8** the first bis(germylene)‐functionalized polystibine complex with the two fused MoGeSb_3_‐heterocycles resembling a distorted, heterocyclic version of a hetero‐bicyclo[3.3.0]octane‐like structure. By means of theoretical methods, the Sb–Sb bonds in **7** and **8** were compared with those in Sb_4_. As with the arsenic homologues, the local force constant in **7** is comparable to that of the single bond in Sb_4_. However, the force constant in **3** is significantly weaker. The strength of the Sb–Sb bonds in **8** represent a borderline case between **7** and **3** (cf. Table S4). Values of the WBI confirm our findings, although the local force constant values represent a much more sensitive indicator on the bond strength.

## Conclusion

**Scheme 3:**
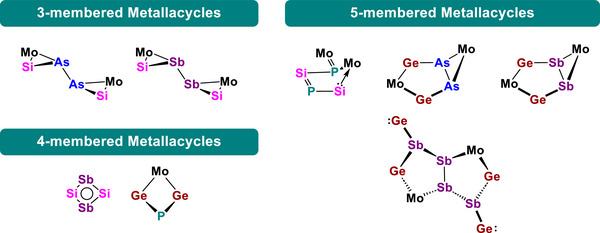
Overview of the obtained ring types of group 14/15 hetero metallacycles incorporating a transition‐metal unit. Stabilizing ligands are omitted for clarity.

In summary, we have investigated the reactivity of organometallic homodipnictogen complexes [{Cp‘Mo(CO)_2_}_2_(μ,η^2:2^‐E_2_)] (E ═ P, As, Sb) towards the low‐valent disilylene [L^Ph^Si]_2_ and digermylene [L^Ph^Ge]_2_. As result, unprecedented three‐, four‐ and five‐membered hetero‐metallacycles and a pentalene‐type hetero‐metallabicycle were obtained (Scheme 3). In contrast to most of the previous reported reactions of these compounds,^[^ ^57^, ^58^, ^59^ ^]^ the Mo–Mo‐bond is broken in all the reactions discussed here. This result is due to the high reactivity of the low‐valent group 14 compounds used as precursors. The results obtained demonstrate a substantial deviation in reactivity compared to the findings of previous investigations.

In detail, by usage of the Si(I) compound, the Mo_2_E_2_ tetrahedrane complexes were converted into a five‐membered MoP_2_Si_2_ heterocycle **1** and the isostructural siladiarsine **2** and siladistibine **3** with two MoAsSi‐/MoSbSi triangles connected by a pnictogen–pnictogen single bond, respectively. Additionally, the heaviest silicon‐pnictogen analogue of cyclobutadiene [(L^Ph^Si)_2_Sb_2_] **4** was isolated within this reaction series. With the heavier tetrel Ge, a novel four‐membered MoGe_2_P heterocycle **5** was obtained from **A**, while the reaction of the Ge(I) compound with the organometallic Mo_2_E_2_ tetrahedranes of arsenic **B** and antimony **C** gave the unprecedented isostructural hetero bicyclo[3.1.0]hexane products **6** and **7** with a three‐membered MoE_2_ ring fused to an MoGe_2_E_2_ heterocycle by shared pnictogen–pnictogen single bonds. Additionally, a novel bis(germylene)‐functionalized polystibine compound **8** with a centrosymmetric, *trans*‐arranged Sb_4_‐chain within a hetero‐bicyclo[3.3.0]octane‐like scaffold was isolated in the same reaction of **C** with [L^Ph^Ge]_2_. In conclusion, by the reaction of the disilylene and digermylene with the tetrahedral Mo_2_E_2_ (E ═ P, As, Sb) complexes an unusual Mo–Mo‐bond cleavage occurs, and the resulting transition‐metal fragments contribute to the formation and stabilization of the unique heavier group 14/15 heterocycles. All reaction products also prove the importance of the transition‐metal fragment as essential part to build unique examples of transition metal incorporated heavier group 14/15 heterocyclic compounds.

## Supporting Information

The authors have cited additional references within the Supporting Information.^[^ ^74^, ^75^, ^76^, ^77^, ^78^, ^79^, ^80^, ^81^, ^82^, ^83^, ^84^, ^85^, ^86^, ^87^, ^88^, ^89^, ^90^, ^91^, ^92^ ^]^

## Conflict of Interests

The authors declare no conflict of interest.

## Supporting information



Supporting information

Supporting information

## Data Availability

Data for this paper are available at radar4chem [https://radar.products.fiz‐karlsruhe.de/] at https://doi.org/10.22000/wgccmd26rdc3zsvj.^[^ ^93^ ^]^
