# Supplementary material for: Pure Molecular Inorganic Rings: Mixed Group 14/15 Metallacycles
Source: Angew Chem Int Ed Engl. 2025 Nov 22;65(4):e20366. doi: 10.1002/anie.202520366 (PMC12828447; doi:10.1002/anie.202520366)
Supplement: Supplementary file 1 — Supporting information [file ANIE-65-e20366-s001.pdf]

Pure Molecular Inorganic Rings: Mixed Group 14/15 Metallacycles

Stefanie Maier,[a] Xiaofei Sun,[a] Lisa Zimmermann,[b] Ralf Köppe,[a] Manfred Scheer,[b] Peter W. Roesky*[a, c]

[a] Institute for Inorganic Chemistry, Karlsruhe Institute of Technology (KIT), Kaiserstr. 12, 76131 Karlsruhe (Germany), E-mail: roesky@kit.edu

[b] Institute of Inorganic Chemistry, University of Regensburg, Universitätsstr. 31, 93040 Regensburg, (Germany).

[c] Institute of Nanotechnology, Karlsruhe Institute of Technology (KIT), Kaiserstr. 12, 76131 Karlsruhe (Germany).

Table of contents

I. Synthesis and characterization S3

I.1 General procedures S3

I.2 Synthesis of compound **1** S4

I.3 Synthesis of compound **2** S5

I.4 Synthesis of compound **3** and **4** S6

I.5 Synthesis of compound **5** S7

I.6 Synthesis of compound **6** S7

I.7 Synthesis of compound **7** S8

I.8 Synthesis of compound **8** S9

II. NMR spectra S10

II.1 NMR spectra of compound **1** S10

II.2 NMR spectra of compound **2** S15

II.3 NMR spectra of compound **3** S17

II.4 NMR spectra of compound **5** S18

II.5 NMR spectra of compound **6** S20

II.6 NMR spectrum of compound **7** S21

III. IR spectra S22

IV. Raman Microscopy S26

IV.1 General methods S26

IV.2 Discussion of the electronic situation in compound **1**, **2** and **3** S26

IV.3 Discussion of the stability of the different ring sizes in compound **1**, **2** and **3** S27

IV.4 Discussion of the electronic situation in compound **I-P**, **I-As** and **4** S28

IV.5 Discussion of the electronic situation in compounds **2** and **6** (As-As bonds) as well as **3**, **7**, and **8** (Sb-Sb bonds) S29

IV.6 Raman spectra S30

V. X-ray crystallography S32

V.1 General methods S32
V.2 Summary of crystal data S33
V.3 Crystal structures S35
V.3.1 Crystal structure of compound **1** S35
V.3.2 Crystal structure of compound **2** S36
V.3.3 Crystal structure of compound **3** S37
V.3.4 Crystal structure of compound **4** S38
V.3.5 Crystal structure of compound **5** S39
V.3.6 Crystal structure of compound **6** S40
V.3.7 Crystal structure of compound **7** S41
V.3.8 Crystal structure of compound **8** S42
VI. Quantum Chemical Calculations S43
VII. References S157

I. Synthesis and characterization

I.1 General procedures

All air- and moisture-sensitive manipulations were performed under dry N_2 or Ar atmosphere using standard Schlenk techniques or in an argon-filled *MBraun* glovebox, unless otherwise stated.

Toluene was dried using an *MBraun* solvent purification system (SPS-800) and degassed prior to use. *n*-Hexane was dried over potassium benzophenone ketyl and distilled under N_2 atomosphere. C_6D_6 and toluene-d_8 were dried over Na-K alloy and CD_2Cl_2 was dried over CaH_2 and degassed by freeze-pump-thaw cycles. All solvents were permanently stored over activated 4 Å molecular sieves.

$[\{Cp^{tBu}Mo(CO)_2\}_2(\mu,\eta^{2:2}\text{-}E_2)]$ (E = P (**A**)[1], As (**B**)[2,3], Sb (**C**)[3,4]; Cp^{tBu} = *t*Bu-C_5H_4), $[L^{Ph}Si]_2$[5] and $[L^{Ph}Ge]_2$[6] (L^{Ph} = PhC(*t*BuN)$_2$) were prepared according to the literature known procedures. All other chemicals were obtained from commercial sources and used without further purification.

NMR spectra were recorded on *Bruker* spectrometers (Avance Neo 400 MHz or Avance III 400 MHz). Chemical shifts are referenced using signals of the residual protio solvent (^1H) or the solvent ($^{13}C\{^1H\}$) and are reported relative to tetramethylsilane (^1H, $^{13}C\{^1H\}$, $^{29}Si\{^1H\}$) or 85 % phosphoric acid ($^{31}P\{^1H\}$). All NMR spectra were measured at 298 K, unless otherwise specified. The multiplicity of the signals is indicated as s = singlet, d = doublet, dd = doublet of doublets, t = triplet, q = quartet, m = multiplet and br = broad. Assignments were determined based on unambiguous chemical shifts, coupling patterns and ^{13}C-DEPT experiments or 2D correlations (^1H-^1H COSY, ^1H-^{13}C HMQC and ^1H-^{13}C HMBC).

Infrared (IR) spectra were recorded in the region 4000-400 cm^{-1} on a *Bruker* Tensor 37 FTIR spectrometer equipped with a room temperature DLaTGS detector and a diamond attenuated total reflection (ATR) unit in an Argon-filled glovebox. In terms of their absolute intensity, the signals were classified into different categories (vs = very strong, s = strong, m = medium, w = weak, and sh = shoulder).

Elemental analyses were carried out with an Elementar vario MICRO cube.

I.2 Synthesis of compound 1

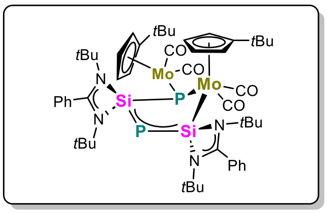

Toluene (ca. 8 mL) was condensed onto a mixture of [{Cp^{tBu}Mo(CO)$_2$}$_2$P$_2$] (60.0 mg, 0.0986 mmol, 1.0 eq.) and [L^{Ph}Si]$_2$ (56.3 mg, 0.109 mmol, 1.1 eq.) at −78 °C. The resulting deep violet solution was warmed up to room temperature and stirred overnight. The solution was concentrated and few yellow crystals of the side product [(L^{Ph}Si)$_2$P$_2$] were removed from the mother liquid by decantation. By very slow evaporation of the solvent, analytically pure violet crystals of **1** were obtained and dried under vaccuum for several hours.

Yield (based on crystals): 65% (72.0 mg, 0.0639 mmol).

Anal. Calcd. For [$C_{52}H_{72}Mo_2N_4O_4P_2Si_2$] (1127.19 g mol^{-1}): C: 55.41, H: 6.44, N: 4.97. Found: C: 55.74, H: 6.29, N: 5.02.

mp 212 °C (dec.)

^{1}H NMR (400.30 MHz, toluene-d_8, <u>298 K</u>): δ (ppm) = 8.19 (br. s, 1H, CH_{Ph}), 7.66 (br. s, 1H, CH_{Ph}), 7.23-7.18 (m, 2H, CH_{Ph}), 7.04-6.99 (m, 3H, CH_{Ph}), 6.96-6.90 (m, 3H, CH_{Ph}), 6.28 (br. s, 1H, Cp^{tBu}-CH_{Ar}), 6.22 (br. s, 1H, Cp^{tBu}-CH_{Ar}), 5.89 (br. s, 1H, Cp^{tBu}-CH_{Ar}), 5.79 (br. s, 1H, Cp^{tBu}-CH_{Ar}), 5.65 (br. s, 1H, Cp^{tBu}-CH_{Ar}), 5.12 (br. s, 1H, Cp^{tBu}-CH_{Ar}), 4.97 (br. s, 1H, Cp^{tBu}-CH_{Ar}), 4.87 (br. s, 1H, Cp^{tBu}-CH_{Ar}), 1.60-1.06 (m, 54H, Cp^{tBu}-C(CH_3)$_3$, NC(CH_3)$_3$).

^{1}H NMR (400.30 MHz, toluene-d_8, <u>273 K</u>): δ (ppm) = 8.22 (m, 1H, CH_{Ph}), 7.66 (m, 1H, CH_{Ph}), 7.16-7.12 m, 2H, CH_{Ph}), 6.97-6.85 (m, 6H, CH_{Ph}), 6.33 (m, 1H, Cp^{tBu}-CH_{Ar}), 6.28 (m, 1H, Cp^{tBu}-CH_{Ar}), 5.91 (br. s, 1H, Cp^{tBu}-CH_{Ar}), 5.74 (m, 1H, Cp^{tBu}-CH_{Ar}), 5.65 (m, 1H, Cp^{tBu}-CH_{Ar}), 5.15 (m, 1H, Cp^{tBu}-CH_{Ar}), 4.98 (m, 1H, Cp^{tBu}-CH_{Ar}), 4.89 (m, 1H, Cp^{tBu}-CH_{Ar}), 1.59 (s, 9H, Cp^{tBu}-C(CH_3)$_3$), 1.46 (s, 9H, NC(CH_3)$_3$), 1.42 (s, 9H, NC(CH_3)$_3$), 1.33 (s, 9H, NC(CH_3)$_3$), 1.24 (s, 9H, Cp^{tBu}-C(CH_3)$_3$), 1.11 (s, 9H, NC(CH_3)$_3$).

^{13}C{^{1}H} NMR (100.61 MHz, toluene-d_8, <u>298 K</u>): δ (ppm) = 252.2 (*C*O), 249.6 (*C*O), 245.5 (*C*O), 239.7 (*C*O), 175.6 (N*C*N), 166.1 (N*C*N), 133.2 ($C_{q,Ar}$), 132.5 ($C_{q,Ar}$), 130.4 (CH_{Ar}), 130.2 (CH_{Ar}), 129.8 (CH_{Ar}), 127.6 (CH_{Ar}), 127.5 (CH_{Ar}), 119.8 ($C_{q,Ar}$), 108.0 (CH_{Ar}), 98.6 (CH_{Ar}), 98.2 (CH_{Ar}), 92.8 (CH_{Ar}), 90.5 (CH_{Ar}), 90.1 (CH_{Ar}), 89.6 (CH_{Ar}), 84.8 (CH_{Ar}), 56.7 (N*C*(CH$_3$)$_3$), 56.1 (N*C*(CH$_3$)$_3$), 55.2 (N*C*(CH$_3$)$_3$), 54.3 (N*C*(CH$_3$)$_3$), 32.3 (Cp^{tBu}-C(*C*H$_3$)$_3$), 31.9 (NC(*C*H$_3$)$_3$), 31.8 (NC(*C*H$_3$)$_3$).

Despite of prolonged acquisition time (>130 000 scans), carbon signals in the ^{13}C{^{1}H} NMR spectra of **1** are not or only poorly resolved at room temperature (298 K). Therefore, assignment of protons as well as carbon atoms was only possible by measuring ^{13}C{^{1}H} and dept135 NMR spectra as well as 2D NMR spectra (^{1}H-^{13}C HMBC, ^{1}H-^{13}C HMQC) at lower temperatures (273 K).

^{13}C{^{1}H} NMR (100.61 MHz, toluene-d_8, <u>273 K</u>): δ (ppm) = 252.8 (*C*O), 249.7 (*C*O), 245.5 (*C*O), 239.2 (*C*O), 175.5 (N*C*N), 166.0 (N*C*N), 133.0 ($C_{q,Ar}$), 132.3 ($C_{q,Ar}$), 130.3 (CH_{Ar}), 130.2 (CH_{Ar}), 129.9 (CH_{Ar}), 129.7 (CH_{Ar}), 127.5 (CH_{Ar}), 127.4 (CH_{Ar}), 119.6 ($C_{q,Ar}$), 108.0 (CH_{Ar}), 98.9 (CH_{Ar}), 98.7 (CH_{Ar}), 92.5 (CH_{Ar}), 90.4 (CH_{Ar}), 90.0 (CH_{Ar}), 89.4 (CH_{Ar}), 84.7 (CH_{Ar}), 56.6 (N*C*(CH$_3$)$_3$), 56.0 (N*C*(CH$_3$)$_3$), 55.0 (N*C*(CH$_3$)$_3$), 54.2 (N*C*(CH$_3$)$_3$), 32.3 (Cp^{tBu}-*C*(CH$_3$)$_3$), 32.2 (Cp^{tBu}-C(*C*H$_3$)$_3$), 32.1 (Cp^{tBu}-C(*C*H$_3$)$_3$), 32.0 (Cp^{tBu}-*C*(CH$_3$)$_3$), 31.7 (NC(*C*H$_3$)$_3$), 31.6 (NC(*C*H$_3$)$_3$).

Due to overlap with the solvent toluene-d_8, four aromatic carbon atoms (C*H*) were assigned by dept135 experiment at 128.8 (CH_{Ar}), 128.1 (CH_{Ar}), 127.2 (CH_{Ar}), 127.1 (CH_{Ar}) and one Cp^{tBu}-C_q carbon atom was only assigned using ^{1}H-^{13}C HMBC experiments at 127.9 ppm.

^{31}P{^{1}H} NMR (162.04 MHz, toluene-d_8, 298 K): δ (ppm) = 555.3 (br. s, 1P, P_X), –148.7 (d, 1P, P_A, $^{2}J_{PP}$ = 56 Hz).

^{31}P{^{1}H} NMR (162.04 MHz, toluene-d_8, 273 K): δ (ppm) = 555.2 (br. s, 1P, P_X), –150.0 (d, 1P, P_A, $^{2}J_{PP}$ = 56 Hz).

^{29}Si{^{1}H} NMR (79.53 MHz, toluene-d_8, 298 K): δ (ppm) = 126.9 (d, 1Si, $^{1}J_{SiP}$ = 113 Hz, Si_B), 59.8-56.0 (m, 1Si, Si_A).

IR (ATR): $\tilde{\nu}$ (cm^{-1}) = 2968 (m), 2933 (sh), 2902 (w), 2867 (w), 1935 (s), 1891 (s), 1845 (s), 1813 (s), 1577 (vw), 1520 (vw), 1474 (w), 1445 (w), 1406 (sh), 1394 (m), 1362 (m), 1269 (m), 1200 (m), 1182 (sh), 1150 (vw), 1082 (w), 1021 (w), 930 (vw), 908 (vw), 892 (vw), 862 (vw), 818 (w), 787 (w), 755 (m), 728 (w), 709 (w), 634 (vw), 616 (sh), 573 (w), 551 (w), 531 (w), 495 (w), 478 (vw), 461 (w), 427 (vw).

I.3 Synthesis of compound 2

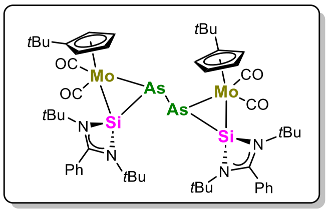

Toluene (ca. 8 mL) was condensed onto a mixture of [{Cp^{tBu}Mo(CO)$_2$}$_2$As$_2$] (50.0 mg, 0.0633 mmol, 1.0 eq.) and [L^{Ph}Si]$_2$ (36.1 mg, 0.0696 mmol, 1.1 eq.) at −78 °C. The resulting dark red solution was warmed up to room temperature and stirred overnight. The solution with a small amount of microcrystalline solid was further concentrated. After storing the concentrated solution at room temperature for several days, the mother liquid was removed. The obtained crystals of **2·(toluene)** were washed twice with small amounts of toluene and *n*-hexane and dried under vacuum for several hours.

Yield (based on crystals): 61% (50.8 mg, 0.0388 mmol).

Anal. Calcd. For [$C_{52}H_{72}As_2Mo_2N_4O_4Si_2$] (1215.09 g mol^{-1}): C: 51.40, H: 5.97, N: 4.61. Found: C: 51.12, H: 5.57, N: 4.52.

mp 178 °C (dec.).

^{1}H NMR (400.30 MHz, DCM-d_2, 298 K): δ (ppm) = 7.62-7.54 (m, 8H, CH_{Ar}), 7.42-7.37 (m, 2H, CH_{Ar}), 7.27-7.21 (m, 2H, toluene-CH_{Ar}), 7.20-7.11 (m, 3H, toluene-CH_{Ar}), 5.42-5.39 (m, 2H, CH_{Ar}), 5.32-5.30 (m, overlapping with residual solvent signal, 2H, CH_{Ar}), 5.11-5.08 (m, 2H, CH_{Ar}), 5.05-5.02 (m, 2H, CH_{Ar}), 2.34 (s, 3H, toluene-CH_3), 1.41 (s, 18H, NC(CH_3)$_3$), 1.35 (s, 18H, Cp^{tBu}-C(CH_3)$_3$), 1.01 (s, 18H, NC(CH_3)$_3$).

Due to decomposition of **2·(toluene)** in DCM-d_2, new signals at 1.08, 1.34 and 1.36 ppm in a ratio of 1:1:1 and four multiplets of the same intensity and similar shape like the Cp^{tBu} multiplets of **2** are observable.

^{13}C{^{1}H} NMR (100.61 MHz, DCM-d_2, 298 K): δ (ppm) = 236.4 (*C*O), 235.1 (*C*O), 171.5 (N*C*N), 138.4 (toluene-$C_{q,Ar}$), 132.2 ($C_{q,Ar}$), 131.2 (CH_{Ar}), 130.1 (CH_{Ar}), 129.4 (CH_{Ar}), 129.0 (CH_{Ar}), 128.60 (toluene-CH_{Ar}), 128.56 (CH_{Ar}), 125.6 (toluene-CH_{Ar}), 123.7 ($C_{q,Ar}$), 91.2 (CH_{Ar}), 90.6 (CH_{Ar}), 87.9 (CH_{Ar}), 87.4 (CH_{Ar}), 55.8 (N*C*(CH$_3$)$_3$), 55.4 (N*C*(CH$_3$)$_3$), 32.5 (NC(*C*H$_3$)$_3$)*, 32.3 (Cp^{tBu}-C(*C*H$_3$)$_3$)*, 32.1 (Cp^{tBu}-*C*(CH$_3$)$_3$), 31.1 (NC(*C*H$_3$)$_3$), 21.6 (toluene-*C*H$_3$).

No definite assignment of the aryl protons of **2**, its decomposition product and toluene could be made. Assignment of the signals marked with * is interchangeable due to signal overlapping in ^{1}H-^{13}C HMBC experiments.

$^{29}Si\{^1H\}$ NMR (79.53 MHz, DCM-d_2, 298 K): δ (ppm) = 118.4 (s, 1Si).

Due to decomposition of **2** in DCM-d_2, a signal of a second Si containing species was detected at 119.0 ppm.

IR (ATR): $\tilde{\nu}$ (cm^{-1}) = 2994 (w), 2960 (sh), 2931 (w), 2866 (w), 2167 (vw), 1885 (s), 1859 (s), 1816 (s), 1798 (s), 1576 (vw), 1518 (vw), 1497 (vw), 1467 (w), 1447 (sh), 1392 (m), 1363 (m), 1275 (w), 1226 (vw), 1197 (w), 1151 (w), 1087 (w), 1072 (vw), 1037 (w), 926 (vw), 905 (vw), 893 (vw), 839 (vw), 788 (w), 761 (m), 741 (w), 706 (w), 681 (vw), 649 (w), 608 (w), 562 (w), 532 (w), 481 (w), 466 (sh).

I.4 Synthesis of compound 3 and 4

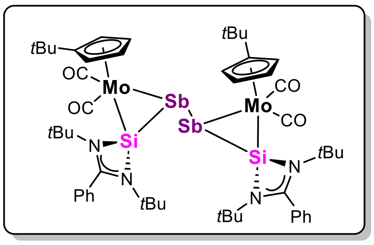

C_6D_6 (ca. 0.4 mL) was condensed onto a mixture of [{$Cp^{tBu}Mo(CO)_2$}$_2Sb_2$] (60.0 mg, 0.0760 mmol, 1.0 eq.) and [$L^{Ph}Si$]$_2$ (43.5 mg, 0.0838 mmol, 1.1 eq.) at −78 °C. The resulting dark red solution was warmed up to room temperature and the NMR tube was carefully shaken until all reactants had been dissolved. For monitoring the reaction, a ^1H NMR spectrum was recorded which confirmed the formation of a set of reaction products. The solution was carefully concentrated to ca. 0.1 mL after 30 min and kept at room temperature until incipient crystallization had started. After two days, single crystals of the title compound **3·(C_6D_6)** could be obtained.

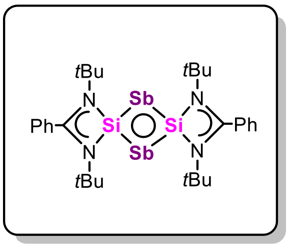

Alongside with compound **3·(C_6D_6)**, a few single crystals of compound **4** were obtained within the reaction of [{$Cp^{tBu}Mo(CO)_2$}$_2Sb_2$] and [$L^{Ph}Si$]$_2$ (see synthesis above). To obtain analytically pure complex **3**, the mother liquid was carefully decanted and the remaining solid was washed twice with small amounts of C_6D_6 and *n*-hexane to remove compound **4**, and the crystals were dried under vacuum for two hours.

Yield of **3·(C_6D_6)** (based on crystals): 12% (11.4 mg, 0.00871 mmol).

Anal. Calcd. For [$C_{52}H_{72}Mo_2N_4O_4Sb_2Si_2$] (1308.76 g mol^{-1}): C: 47.72, H: 5.55, N: 4.28. Found C: 47.71, H: 5.356, N: 3.14.

Compound **3** showed no solubility in deuterated organic solvents like C_6D_6 and THF-d_8. Instead, it was soluble in DCM-d_2 but showed rapid decomposition according to ^1H NMR measurements to a range of non-identifiable products preventing any kind of NMR spectroscopic characterization.

mp 160 °C (dec.).

IR (ATR): $\tilde{\nu}$ (cm^{-1}) = 2995 (vw), 2961 (sh), 2902 (w), 2867 (w), 2284 (vw), 2164 (vw), 1981 (vw), 1884 (s), 1867 (s), 1821 (s), 1807 (s), 1807 (s), 1577 (vw), 1517 (vw), 1481 (vw), 1468 (w), 1456 (w), 1445 (w), 1391 (m), 1363 (m),1331 (vw), 1275 (w), 1239 (vw), 1226 (vw), 1199 (w), 1152 (vw), 1088 (vw), 1072 (vw), 1040 (w), 1023 (vw), 927 (vw), 904 (vw), 893 (vw), 845 (vw), 809 (vw), 789 (w), 769 (w), 760 (m), 708 (m), 678 (vw), 644 (w), 609 (w), 561 (w), 531 (w), 493 (w), 468 (w).

Due to the different solubility, **3** and **4** could be separated easily, but the low amounts of crystals of **4** prevented any further analysis.

I.5 Synthesis of compound 5

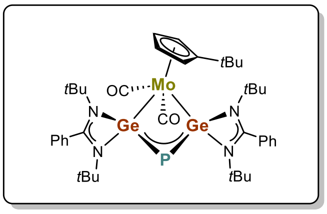

C_6D_6 (ca. 0.4 mL) was condensed onto a mixture of [{Cp^{tBu}Mo(CO)$_2$}$_2$P$_2$] (60.0 mg, 0.09864 mmol, 1.0 eq.) and [L^{Ph}Ge]$_2$ (66.0 mg, 0.109 mmol, 1.1 eq.) at −78 °C. The resulting red solution was warmed up to room temperature and the NMR tube was carefully shaken until all reactants had been dissolved. For monitoring the reaction, a ^{1}H and ^{31}P{^{1}H} NMR spectrum was recorded which confirmed the formation of a set of reaction products. The solution was carefully concentrated to ca. 0.1 mL after 9 days and kept at room temperature until incipient crystallization had started. After two days, single crystals of the title compound **5·(C_6D_6)** could be obtained. The mother liquid was carefully decanted and the remaining solid was washed twice with small amounts of C_6D_6 and *n*-hexane and dried under vacuum for two hours.

Yield (based on crystals): 13% (12.4 mg, 0.0986 mmol).

Anal. Calcd. For [$C_{41}H_{59}Ge_2MoN_4O_2P$] (912.13 g mol^{-1}): no satisfactory analysis could be obtained.

mp 203 °C (dec.).

^{1}H NMR (400.3 MHz, C_6D_6): δ (ppm) = 7.63-7.57 (m, 2H, CH_{Ar}), 7.50-7.46 (m, 2H, CH_{Ar}), 7.03-6.97 (m, 6H, CH_{Ar}), 5.26-5.24 (m, 2H, CH_{Ar}), 5.22-5.20 (m, 1H, CH_{Ar}), 5.17-5.15 (m, 1H, CH_{Ar}), 1.38 (s, 9H, NC(CH_3)$_3$), 1.37-1.36 (m, 12H, Cp^{tBu}-C(CH_3)$_3$, NC(CH_3)$_3$), 1.29 (s, 18H, NC(CH_3)$_3$).

^{13}C{^{1}H} NMR (100.61 MHz, C_6D_6, 298 K): δ (ppm) = 239.3 (*C*O), 170.2 (N*C*N), 150.4 (N*C*N), 134.9 ($C_{q,Ar}$), 131.0 (CH_{Ar}), 130.5 (*C*H_{Ar}), 129.7 (*C*H_{Ar}), 127.5 (*C*H_{Ar}), 127.4 (*C*H_{Ar}), 125.2 ($C_{q,Ar}$), 121.9 ($C_{q,Ar}$), 88.7 (*C*H_{Ar}), 88.5 (*C*H_{Ar}), 88.3 (*C*H_{Ar}), 85.4 (*C*H_{Ar}), 55.4 (N*C*(CH$_3$)$_3$), 54.8 (N*C*(CH$_3$)$_3$), 32.8 (NC(*C*H$_3$)$_3$), 32.5 (Cp^{tBu}-C(*C*H$_3$)$_3$), 32.3 (Cp^{tBu}-*C*(*C*H$_3$)$_3$), 31.5 (NC(*C*H$_3$)$_3$).

^{31}P{^{1}H} NMR (162.04 MHz, C_6D_6): -44.0 (s).

IR (ATR): $\tilde{\nu}$ (cm^{-1}) = 2960 (s), 2960 (s), 2903 (m), 2866 (w), 1982 (m), 1952 (s), 1889 (vs), 1843 (s), 1813 (s), 1644 (w), 1516 (vw), 1475 (m), 1459 (m), 1444 (m), 1415 (m), 1391 (m), 1360 (m), 1269 (w), 1250 (m), 1200 (m), 1148 (w), 1064 (w), 1039 (vw), 1019 (w), 926 (vw), 907 (vw), 889 (vw), 790 (m), 739 (w), 706 (m), 576 (w), 553 (w), 532 (vw), 518 (vw), 498 (vw), 449 (w).

I.6 Synthesis of compound 6

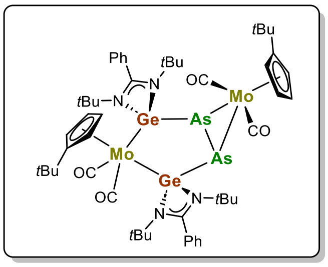

C_6D_6 (ca. 0.4 mL) was condensed onto a mixture of [{Cp^{tBu}Mo(CO)$_2$}$_2$As$_2$] (60.0 mg, 0.0760 mmol, 1.0 eq.) and [L^{Ph}Ge]$_2$ (57.6 mg, 0.0947 mmol, 1.1 eq.) at −78 °C. The resulting red solution was warmed up to room temperature and the NMR tube was carefully shaken until all reactants had been dissolved. For monitoring the reaction, a ^{1}H NMR spectrum was recorded which confirmed the formation of a set of reaction products. The

solution was carefully concentrated to ca. 0.1 mL and kept at room temperature until incipient crystallization had started. After one day, single crystals of the title compound **6** could be obtained. The mother liquid was carefully decanted and the remaining solid was washed twice with small amounts of C_6D_6 and *n*-hexane and dried under vacuum for two hours.

Yield (based on crystals): 19% (24.3 mg, 0.0161 mmol).

Anal. Calcd. For [$C_{52}H_{72}As_2Ge_2Mo_2N_4O_4$] (1304.18 g mol^{-1}) C: 47.89; H: 5.56; N: 4.30. Found: C 46.96; H 5.22; N 4.32.

mp 180 °C (dec.).

^{1}H NMR (400.30 MHz, C_6D_6): δ (ppm) = 7.55-7.50 (m, 2H, CH_{Ar}), 7.50-7.46 (m, 2H, CH_{Ar}), 7.03-6.96 (m, 6H, CH_{Ar}), 5.22-5.20 (m, 2H, CH_{Ar}), 5.19-5.15 (m, 6H, CH_{Ar}), 1.38 (s, 9H, NC(CH_3)$_3$), 1.36 (s, 9H, Cp^{tBu}-C(CH_3)$_3$), 1.32 (s, 9H, Cp^{tBu}-C(CH_3)$_3$), 1.30 (s, 18H, NC(CH_3)$_3$).

^{13}C{^{1}H} NMR (100.61 MHz, C_6D_6): δ (ppm) = 245.6 (*C*O), 239.7 (*C*O), 168.9 (N*C*N), 134.9 (N*C*N), 130.8 (*C*H_{Ar}), 130.6 (*C*H_{Ar}), 129.7 (*C*H_{Ar}), 128.7 (*C*$_{q,Ar}$), 127.5 (*C*H_{Ar}), 127.4 (*C*$_{q,Ar}$), 125.1 (*C*$_{q,Ar}$), 121.5 (Cp^{tBu}-*C*(CH$_3$)$_3$), 88.8 (*C*H_{Ar}), 87.6 (*C*H_{Ar}), 85.9 (*C*H_{Ar}), 85.5 (*C*H_{Ar}), 55.5 (N*C*(CH$_3$)$_3$), 54.7 (N*C*(CH$_3$)$_3$), 32.9 (NC(*C*H$_3$)$_3$), 32.6 (NC(*C*H$_3$)$_3$), 32.3 (Cp^{tBu}-*C*(CH$_3$)$_3$), 32.0 (NC(*C*H$_3$)$_3$), 31.8 (Cp^{tBu}-*C*(CH$_3$)$_3$), 31.5 (Cp^{tBu}-C(*C*H$_3$)$_3$).

IR (ATR): $\tilde{\nu}$ (cm^{-1}) = 2959 (m), 2902 (w), 2866 (w), 2166 (vw), 1893 (m), 1835 (m), 1794 (m), 1759 (m), 1642 (m), 1477 (w), 1442 (m), 1424 (m), 1392 (w), 1359 (m), 1270 (w), 1254 (w), 1200 (w), 1147 (vw), 1068 (w), 1037 (vw), 1020 (w), 908 (vw), 788 (w), 741 (w), 705 (w), 558 (w), 522 (vw), 503 (vw), 481 (w).

I.7 Synthesis of compound 7

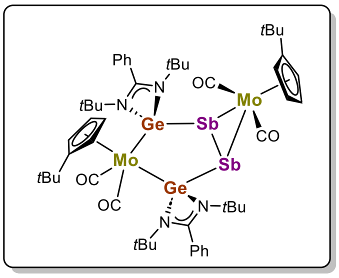

THF (ca. 10 mL) was condensed onto a mixture of [{Cp^{tBu}Mo(CO)$_2$}$_2$Sb$_2$] (50.0 mg, 0.0633 mmol, 1.0 eq.) and [L^{Ph}Ge]$_2$ (42.3 mg, 0.0696 mmol, 1.1 eq.) at −78 °C. The resulting dark orange solution was warmed up to room temperature and stirred for four days. The solution was concentrated and layered with *n*-hexane. After one week, red crystals of 7**·1.5(thf)** were obtained from the THF/*n*-hexane solution, the mother liquor was carefully removed and the crystals dried under vacuum for several hours.

Yield (based on crystals): 49% (47.1 mg, 0.0313 mmol).

Anal. Calcd. For [$C_{52}H_{72}Ge_2Mo_2N_4O_4Sb_2$](1397.85 g mol^{-1}): C: 44.68; H: 5.19; N: 4.01. Found: C: 44.74; H: 4.36; N: 4.22.

mp 177 °C (dec.).

^{1}H NMR (400.30 MHz, C_6D_6): δ (ppm) = 7.51-7.44 (m, 4H, CH_{Ar}), 7.04-6.96 (m, 6H, CH_{Ar}), 5.16 (dd, $^3J_{HH}$ = 2.2 Hz, $^4J_{HH}$ = 2.2 Hz, 2H, CH_{Ar}), 5.11 (dd, $^3J_{HH}$ = 2.2 Hz, $^4J_{HH}$ = 2.2 Hz, 2H, CH_{Ar}), 5.05 (dd, $^3J_{HH}$ = 2.2 Hz, $^4J_{HH}$ = 2.2 Hz, 2H, CH_{Ar}), 5.00 (dd, $^3J_{HH}$ = 2.2 Hz, $^4J_{HH}$ = 2.2 Hz, 2H, CH_{Ar}), 1.37 (s, 18H, NC(CH_3)$_3$), 1.33 (s, 9H, Cp^{tBu}-C(CH_3)$_3$), 1.30 (s, 18H, NC(CH_3)$_3$), 1.27 (s, 9H, Cp^{tBu}-C(CH_3)$_3$).

$^{13}C\{^{1}H\}$ NMR (100.61 MHz, C_6D_6): δ (ppm) = 243.9 (*C*O), 240.5 (*C*O), 166.7 (N*C*N), 135.0 (N*C*N), 130.9 (*C*H_{Ar}), 130.4 (*C*H_{Ar}), 129.7 (*C*H_{Ar}), 128.7 (*C*$_{q,Ar}$), 127.5 (*C*H_{Ar}), 127.4 (*C*H_{Ar}), 124.7 ($C_{q,Ar}$), 120.8 ($C_{q,Ar}$), 88.9 (*C*H_{Ar}), 85.6 (*C*H_{Ar}), 85.1 (*C*H_{Ar}), 83.2 (*C*H_{Ar}), 55.6 (N*C*$(CH_3)_3$), 54.6 (N*C*$(CH_3)_3$), 33.0 (NC(*C*$H_3)_3$), 32.7 (Cp^{tBu}-C(*C*$H_3)_3$), 32.3 (NC(*C*$H_3)_3$), 32.1 (Cp^{tBu}-*C*$(CH_3)_3$), 31.8 (Cp^{tBu}-*C*$(CH_3)_3$), 31.5 (Cp^{tBu}-C(*C*$H_3)_3$).

IR (ATR): $\tilde{\nu}$ (cm^{-1}) = 2960 (m), 2932 (w), 2904 (w), 2868 (w), 2166 (vw), 1928 (m), 1855 (s), 1785 (w), 1609 (w), 1460 (w), 1445 (w), 1419 (m), 1394 (w), 1362 (w), 1256 (vw), 1197 (w), 1148 (vw), 1072 (vw), 1037 (vw), 1021 (vw), 788 (w), 744 (w), 706 (w), 585 (vw), 555 (w), 478 (vw).

I.8 Synthesis of compound 8

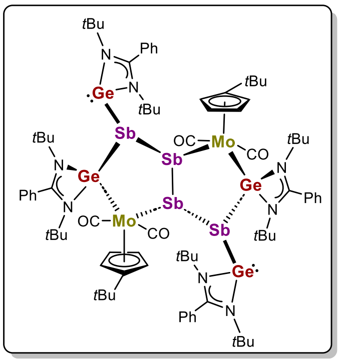

THF (ca. 10 mL) was condensed onto a mixture of [$\{Cp^{tBu}Mo(CO)_2\}_2Sb_2$] (50.0 mg, 0.0633 mmol, 1.0 eq.) and [$L^{Ph}Ge]_2$ (42.3 mg, 0.0696 mmol, 1.1 eq.) at −78 °C. The resulting dark orange solution was warmed up to room temperature and stirred for four days. The solution was concentrated. By slow evaporation of the solvent, analytically pure red crystals of **8·5(thf)** were obtained, washed with small amounts of THF and *n*-hexane and dried under vaccuum for several hours.

Yield (based on crystals): 11% (17.0 mg, 0.00689 mmol).

Anal. Calcd. For [$C_{82}H_{118}Ge_4Mo_2N_8O_4Sb_4$] (2249.36 g mol^{-1}): C: 43.79; H: 5.29; N: 4.98. Found: C: 43.37; H: 5.021; N: 4.58. Repeated elemental analysis of **8·5(thf)** indicated the complete loss of all molecules of thf during the measurement.

Compound **8** showed a very low solubility in deuterated organic solvents like C_6D_6, THF-d_8 and chlorinated solvents like DCM-d_2 and $CDCl_3$. Instead, only signals of multiple decomposition products accompanied with darkening of the previously clear colourless solution above the non-dissolved crystals of compound **8** was obtained, preventing any kind of NMR spectroscopic characterization.

mp 186 °C (dec.).

IR (ATR): $\tilde{\nu}$ (cm^{-1}) = 3432 (vw), 3062 (vs), 2959 (m), 2865 (vs), 2166 (vs), 1884, (s), 1822 (m), 1643 (s), 1610 (vs), 1579 (vw), 1475 (w), 1428 (m), 1388 (w), 1358 (s), 1290 (vw), 1251 (s), 1198 (s), 1142 (vw), 1067 (s), 1032(vw), 1019 (vs), 928 (vs), 790 (s), 739 (s), 705 (s), 579 (vs), 558 (w), 512 (w), 485 (vw), 466 (vw).

II. NMR spectra

II.1 NMR spectra of compound 1

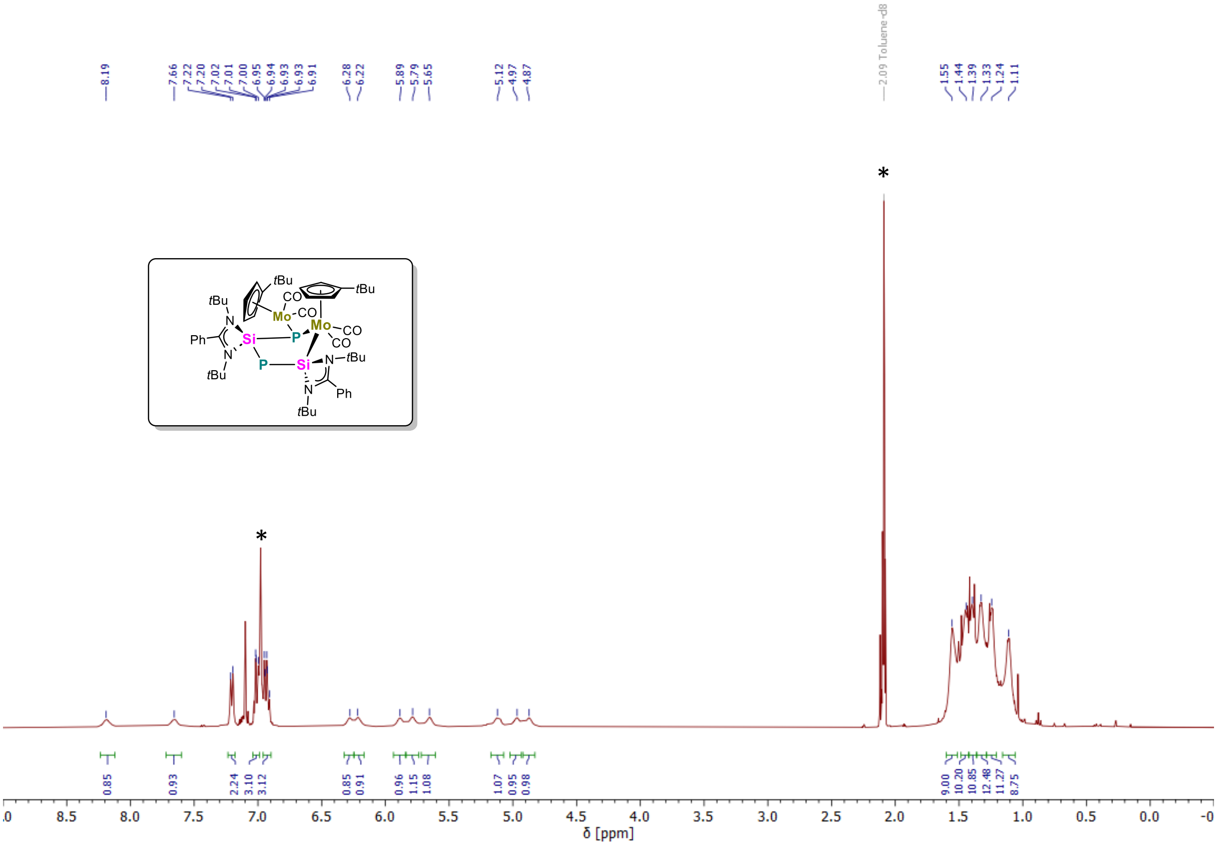

Figure S1. ^{1}H NMR spectrum of **1** in toluene-d_8 at 298 K; * residual protio solvent signal.

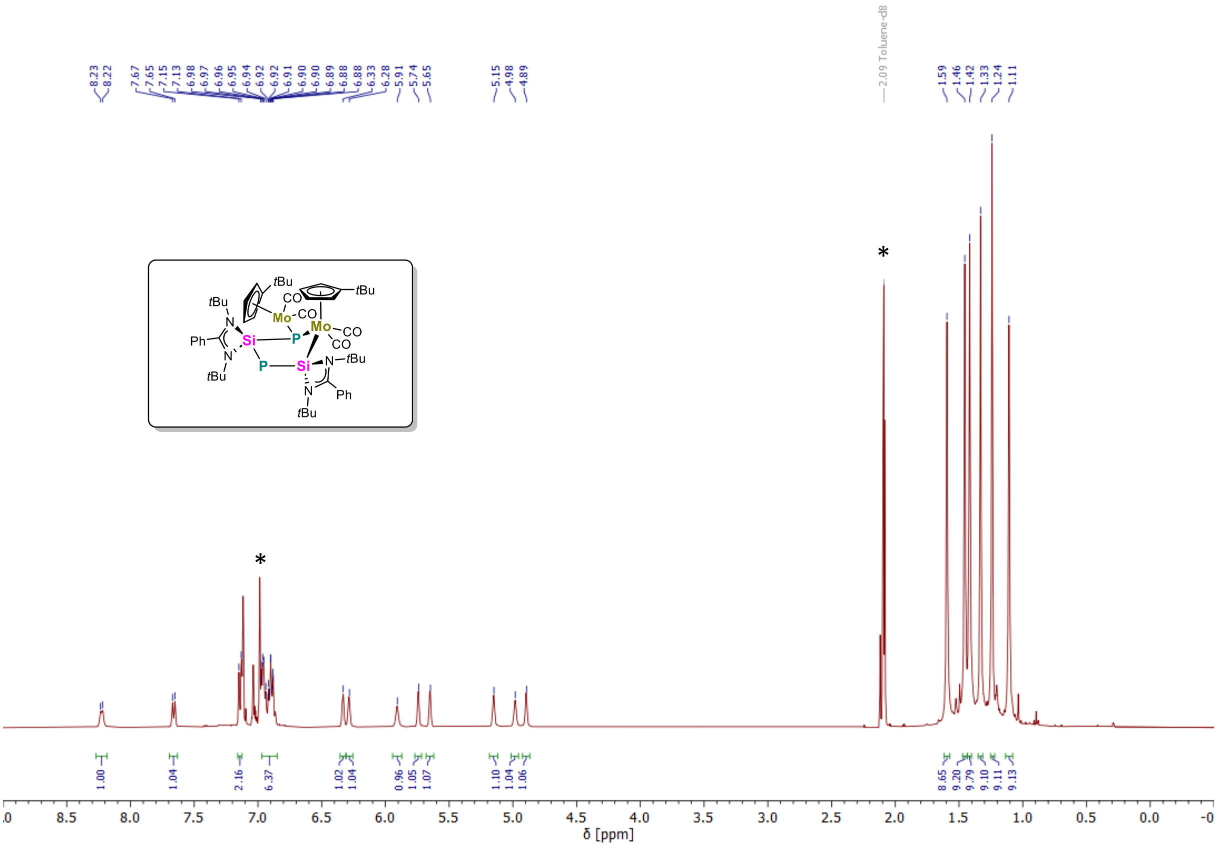

Figure S2.^{1}H NMR spectrum of **1** in toluene-d_8 at 273 K; * residual protio solvent signal.

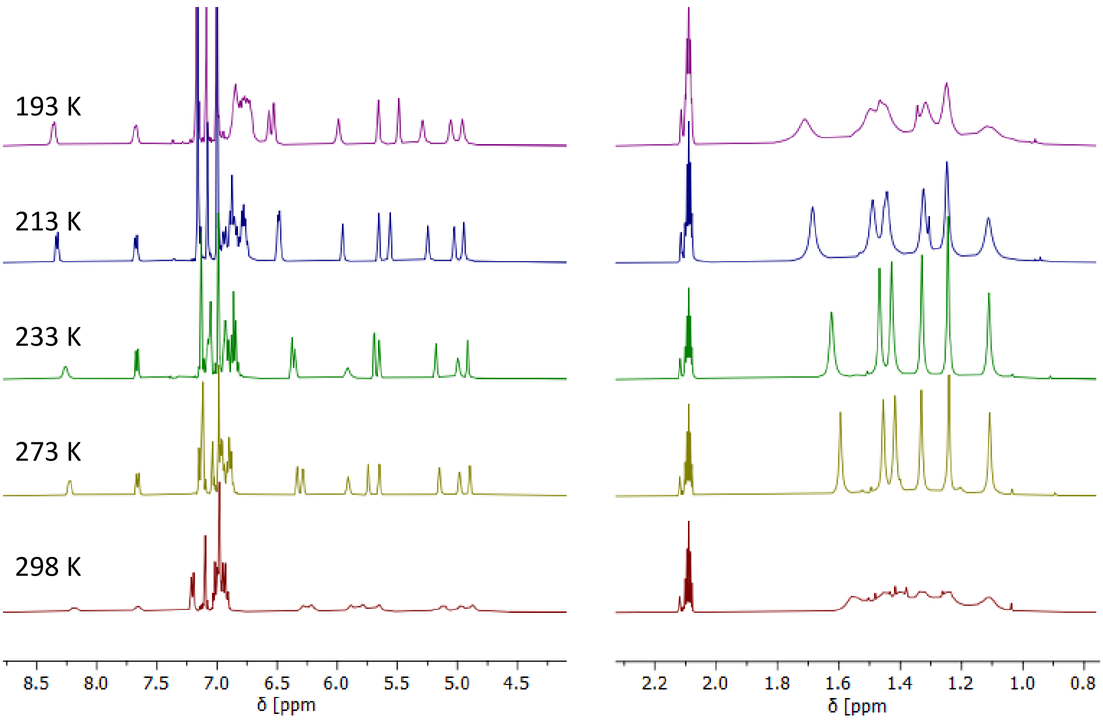

Figure S3. ^{1}H-VT-NMR studies of a solution of **1** in toluene-d_8 at various temperatures.

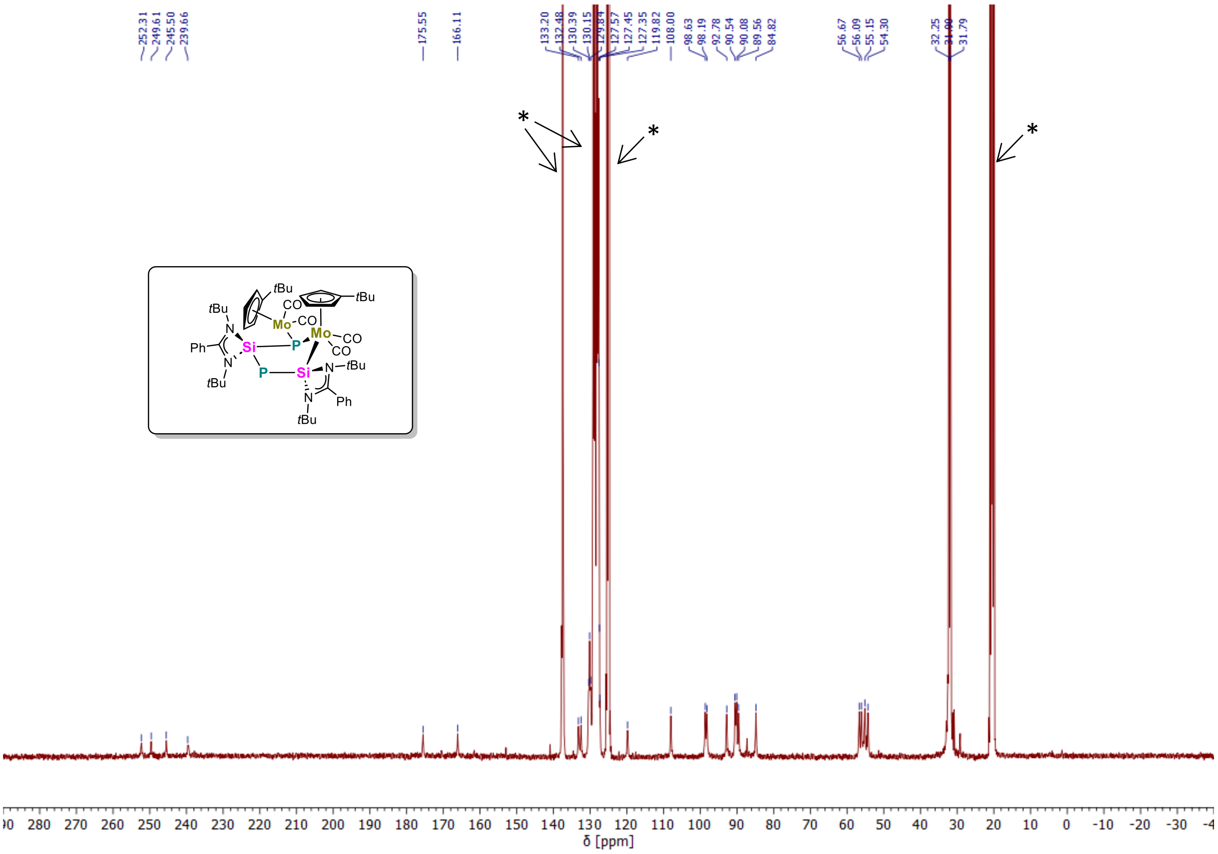

Figure S4.^{13}C{^{1}H} NMR spectrum of **1** in toluene-*d*8 <u>at 298 K</u>; * toluene-*d*8.

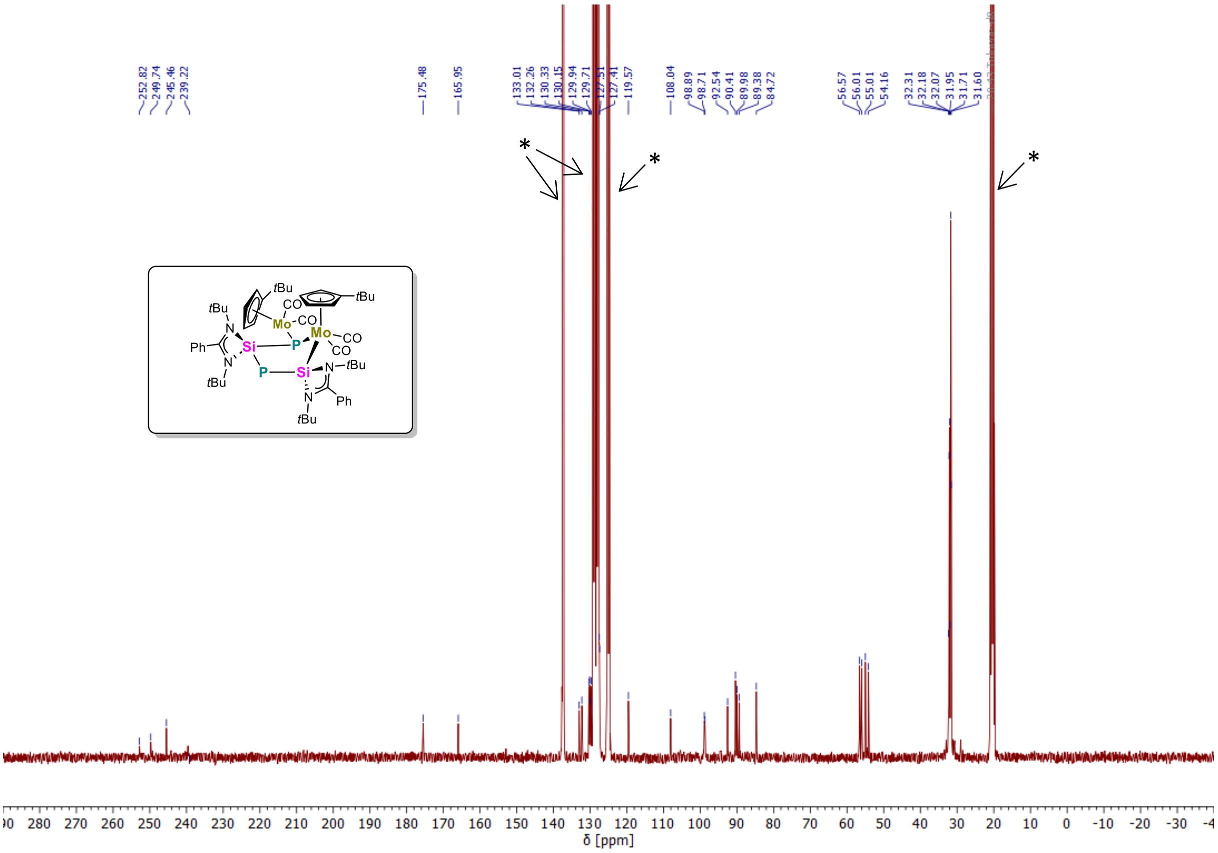

Figure S5. ^{13}C{^{1}H} NMR spectrum of **1** in toluene-*d*8 <u>at 273 K; </u>* toluene-*d*8.

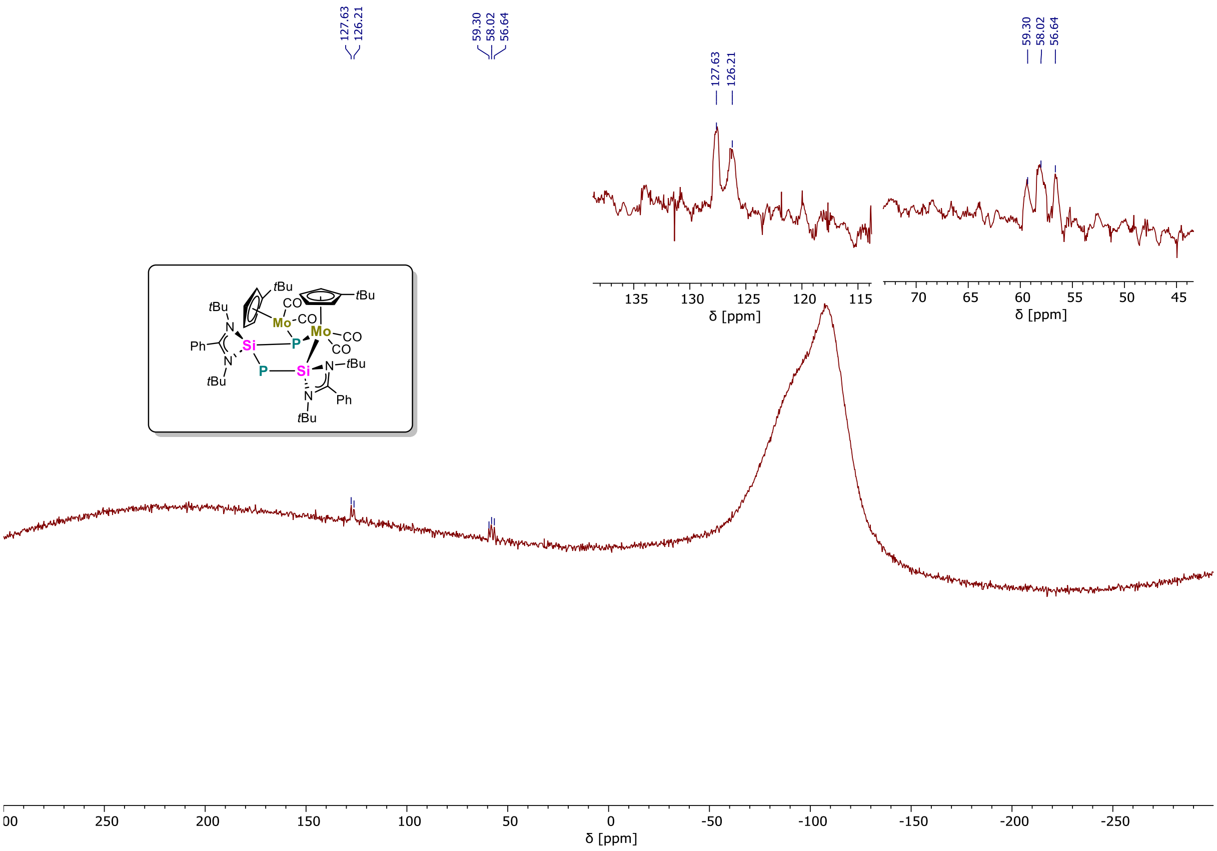

Figure S6. $^{29}Si\{^{1}H\}$ NMR spectrum of **1** in toluene-d_8 <u>at 298 K</u>.

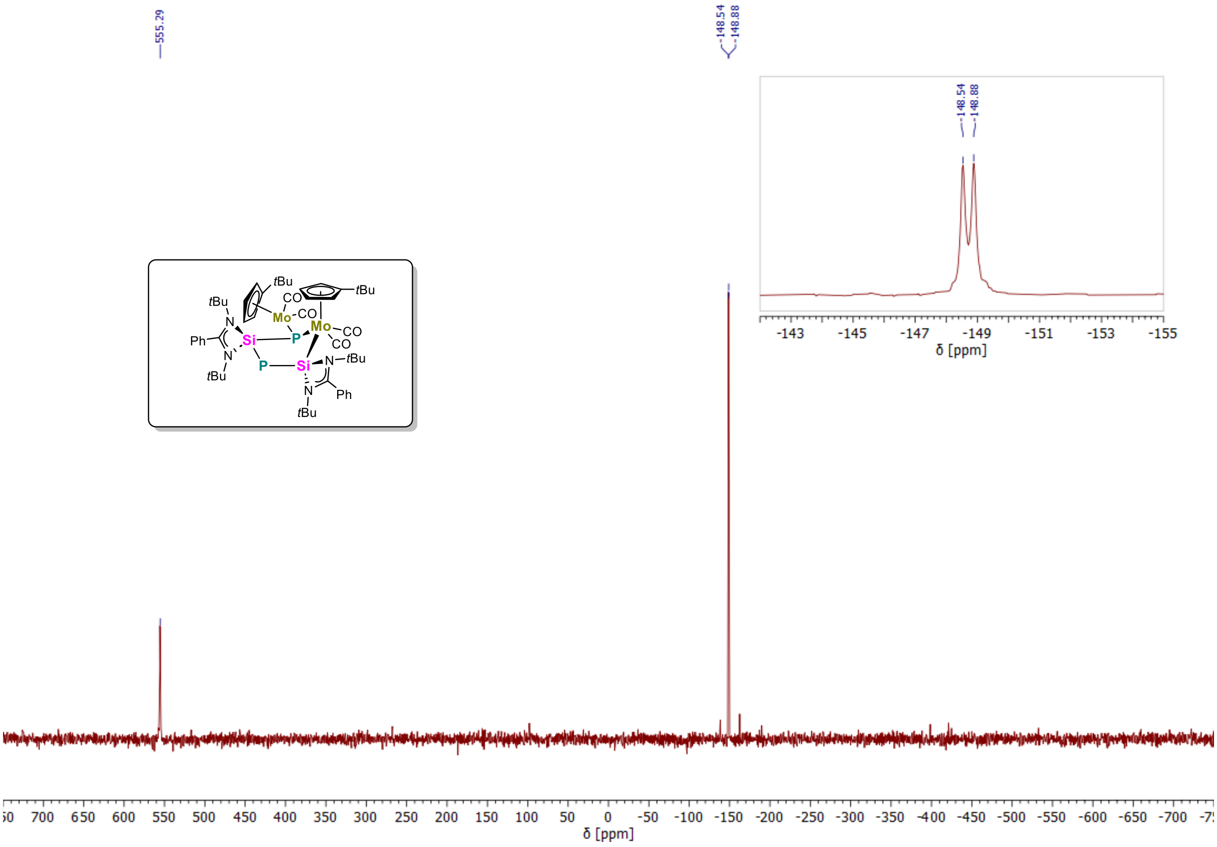

Figure S7. $^{31}P\{^{1}H\}$ NMR spectrum of **1** in toluene-d_8 <u>at 298 K</u>.

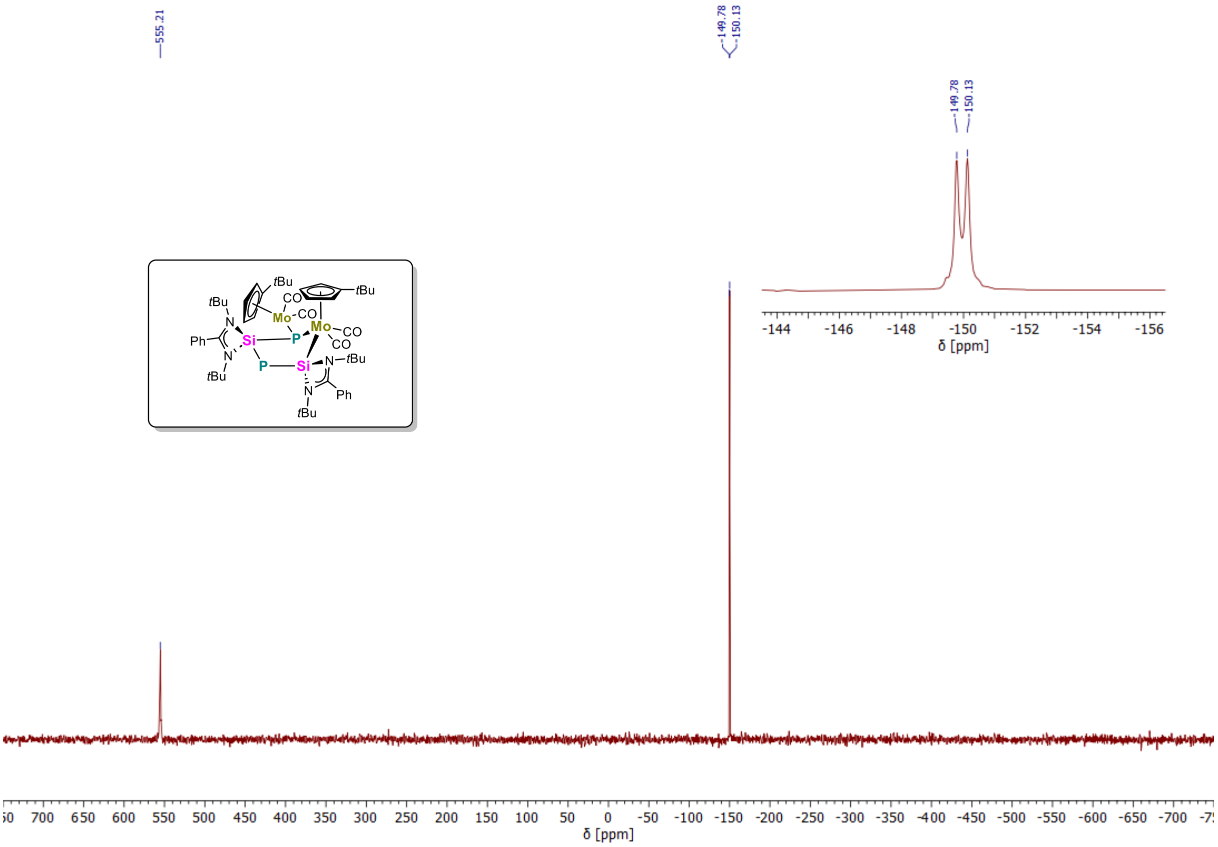

Figure S8. $^{31}P\{^{1}H\}$ NMR spectrum of **1** in toluene-d_8 at 273 K.

II.2 NMR spectra of compound 2

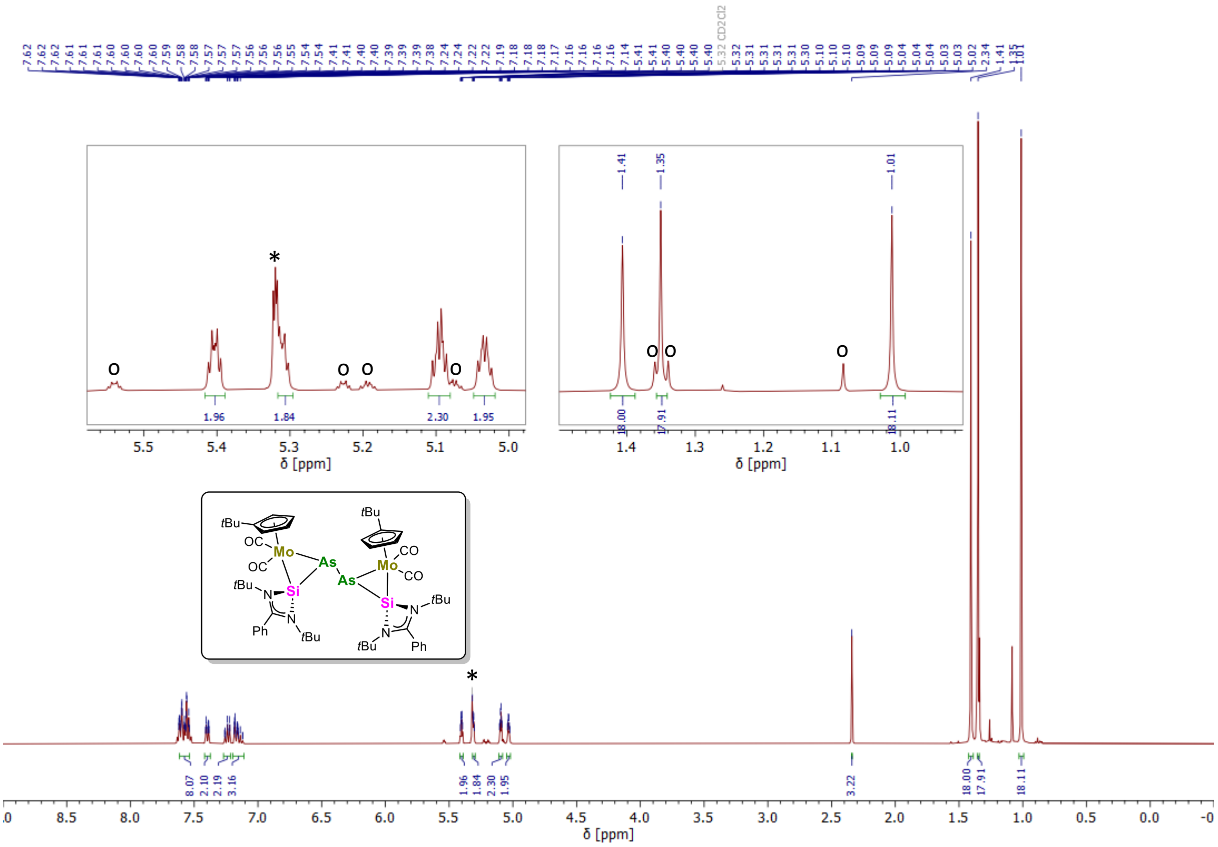

Figure S9. ^{1}H NMR spectrum of **2** in DCM-d_2; * residual protio solvent signal; o signals of decomposition present after ca. 10 min in a DCM-d_2 solution.

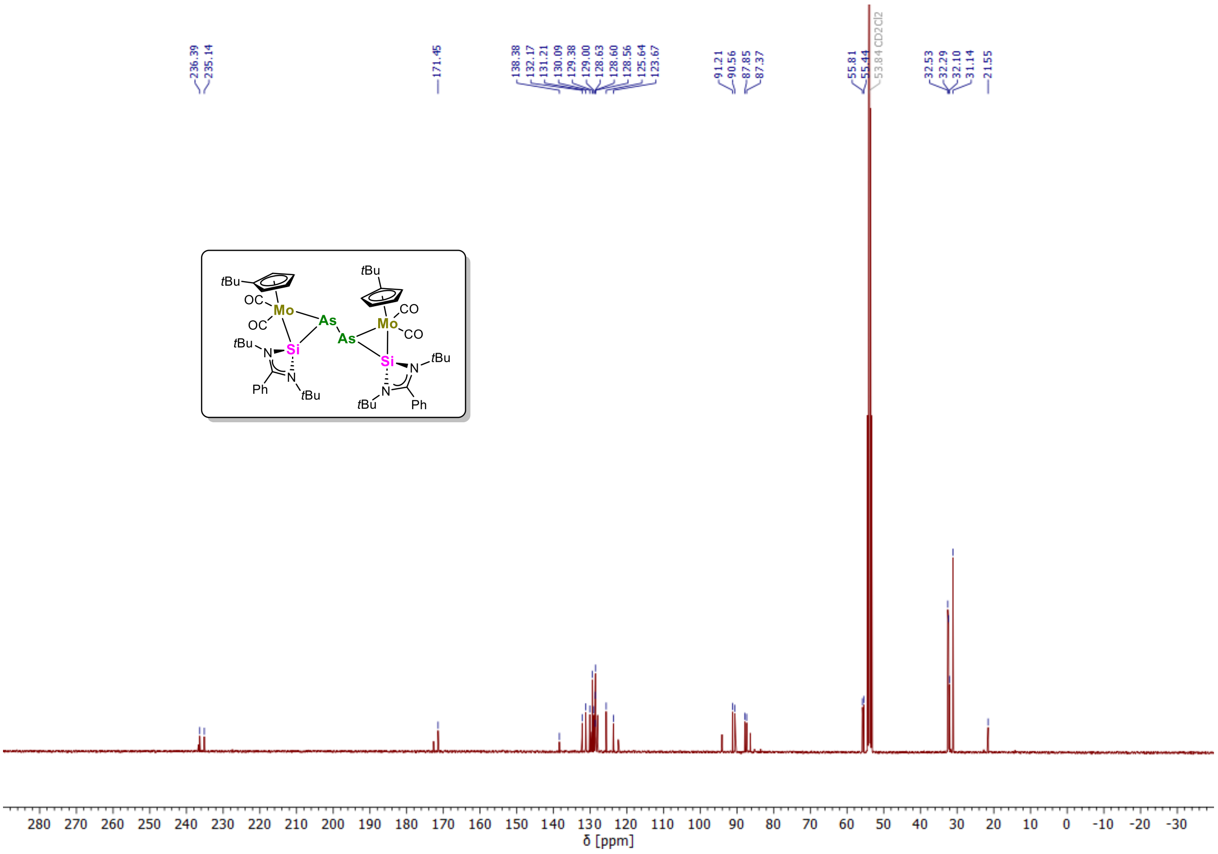

Figure S10. $^{13}C\{^{1}H\}$ NMR spectrum of **2** in DCM-d_2. * DCM-d_2.

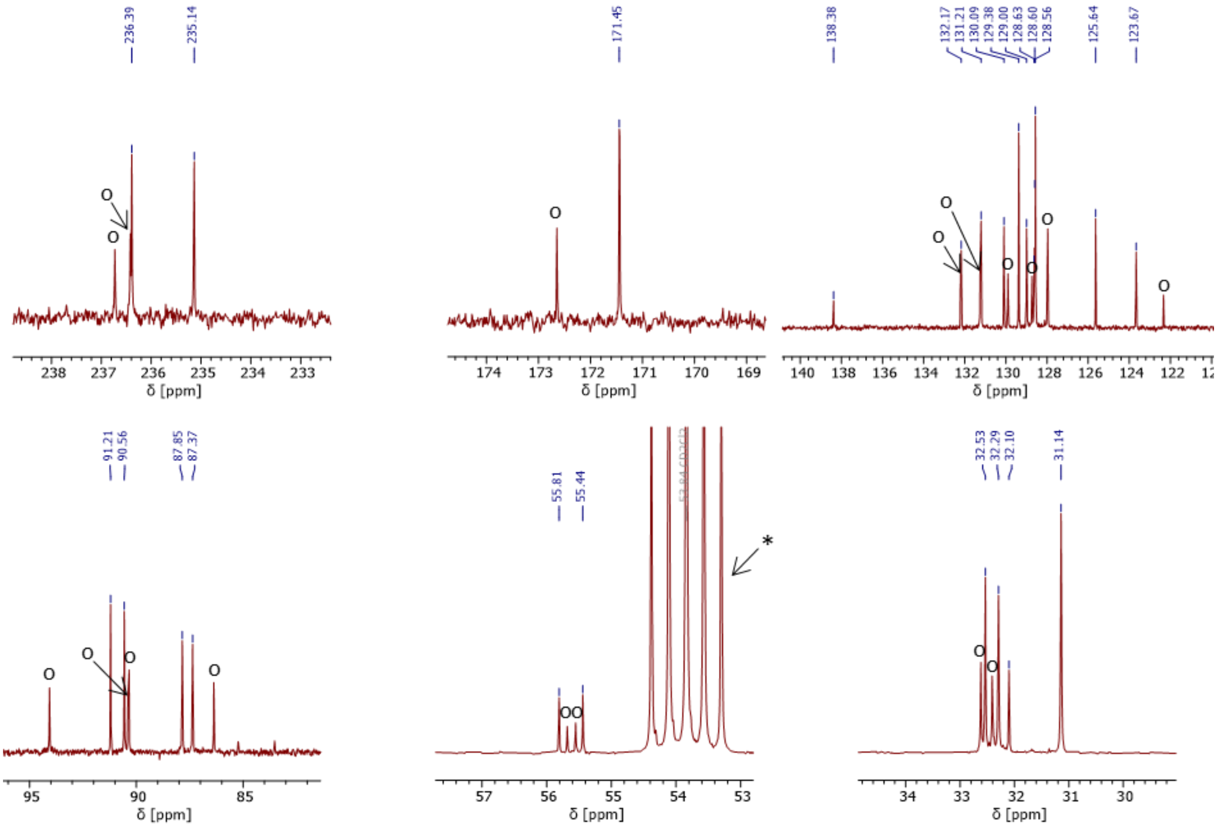

Figure S11. Expansion of the $^{13}C\{^1H\}$ NMR spectrum of **2** in DCM-d_2. * DCM-d_2, o signals of decomposition present in a DCM-d_2 solution.

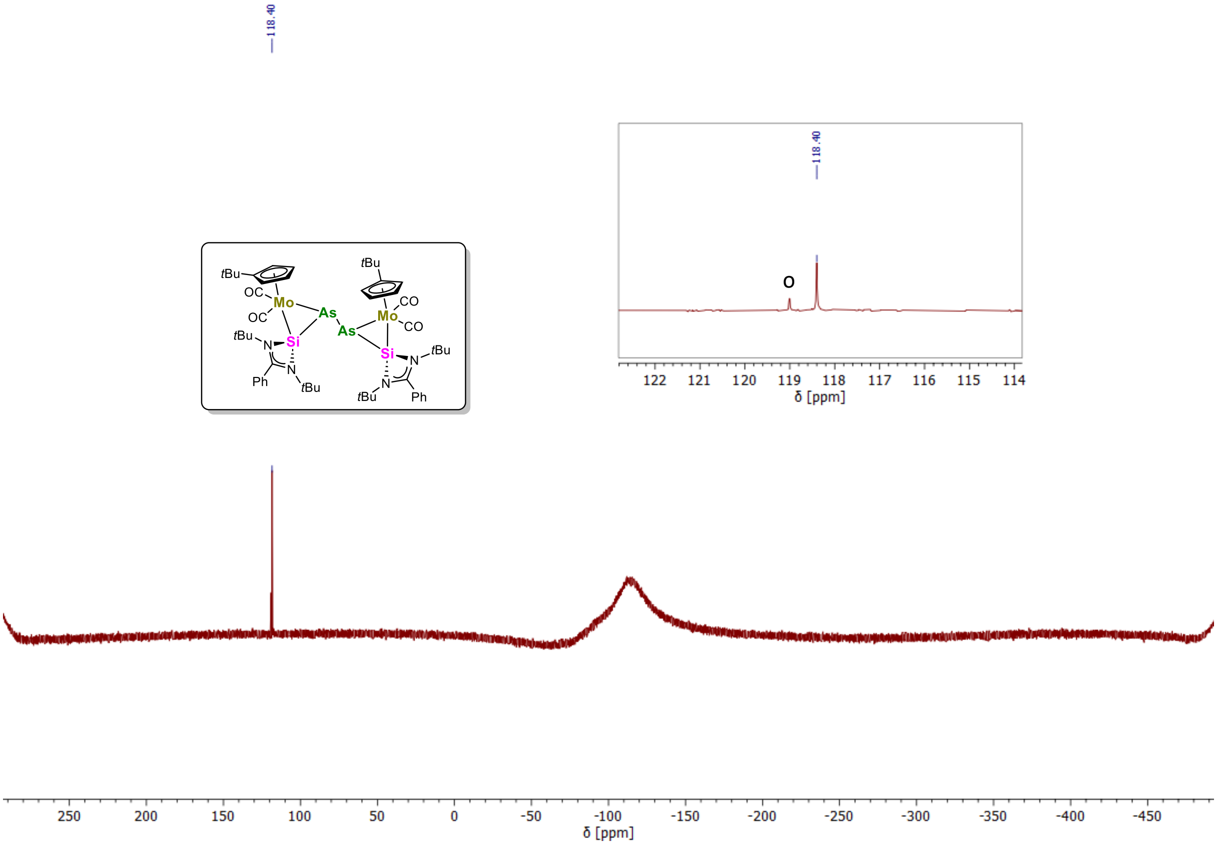

Figure S12. $^{29}Si\{^1H\}$ NMR spectrum of **2** in DCM-d_2; o signal of decomposition present in a DCM-d_2 solution.

II.3 NMR spectra of compound 3

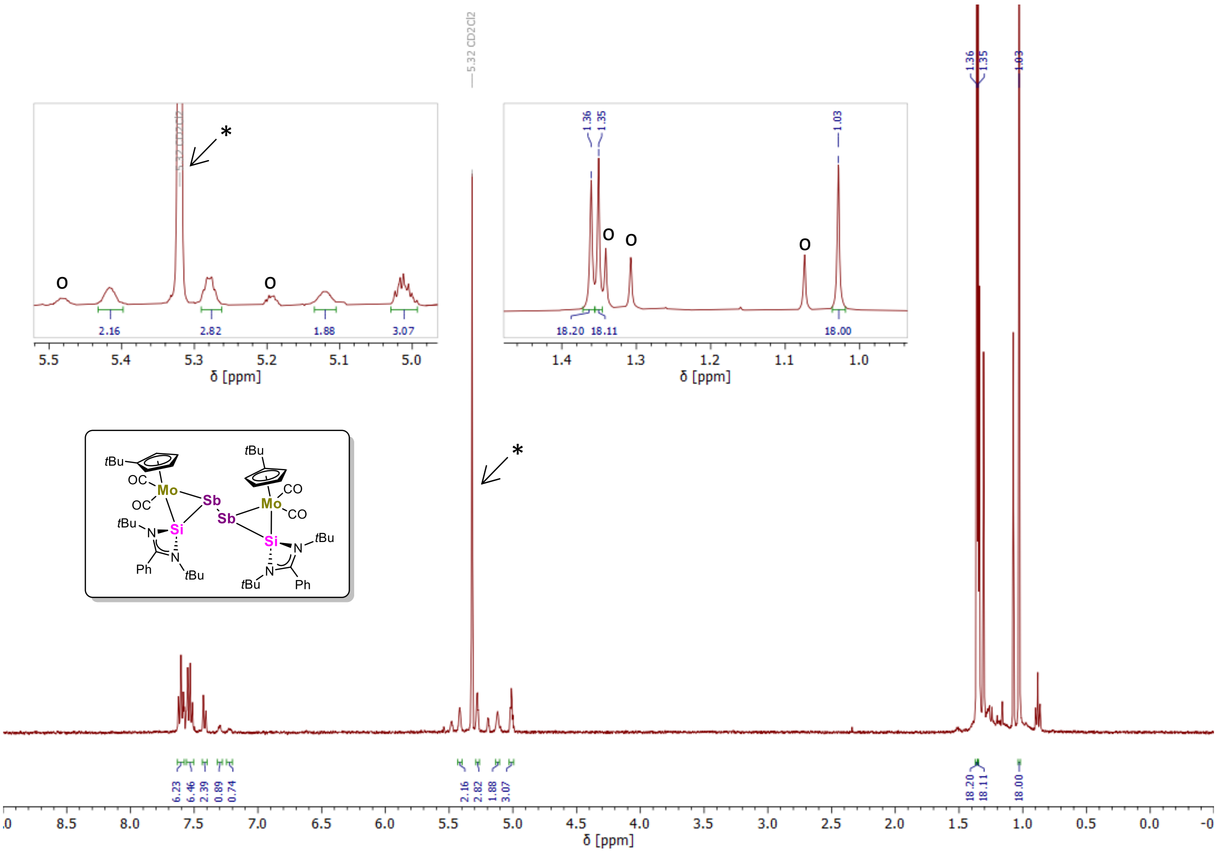

Figure S13. ^{1}H NMR spectrum of **3** in DCM-d_2; * residual protio solvent signal; o signals of decomposition present after ca. 10 min in a DCM-d_2 solution.

II.4 NMR spectra of compound 5

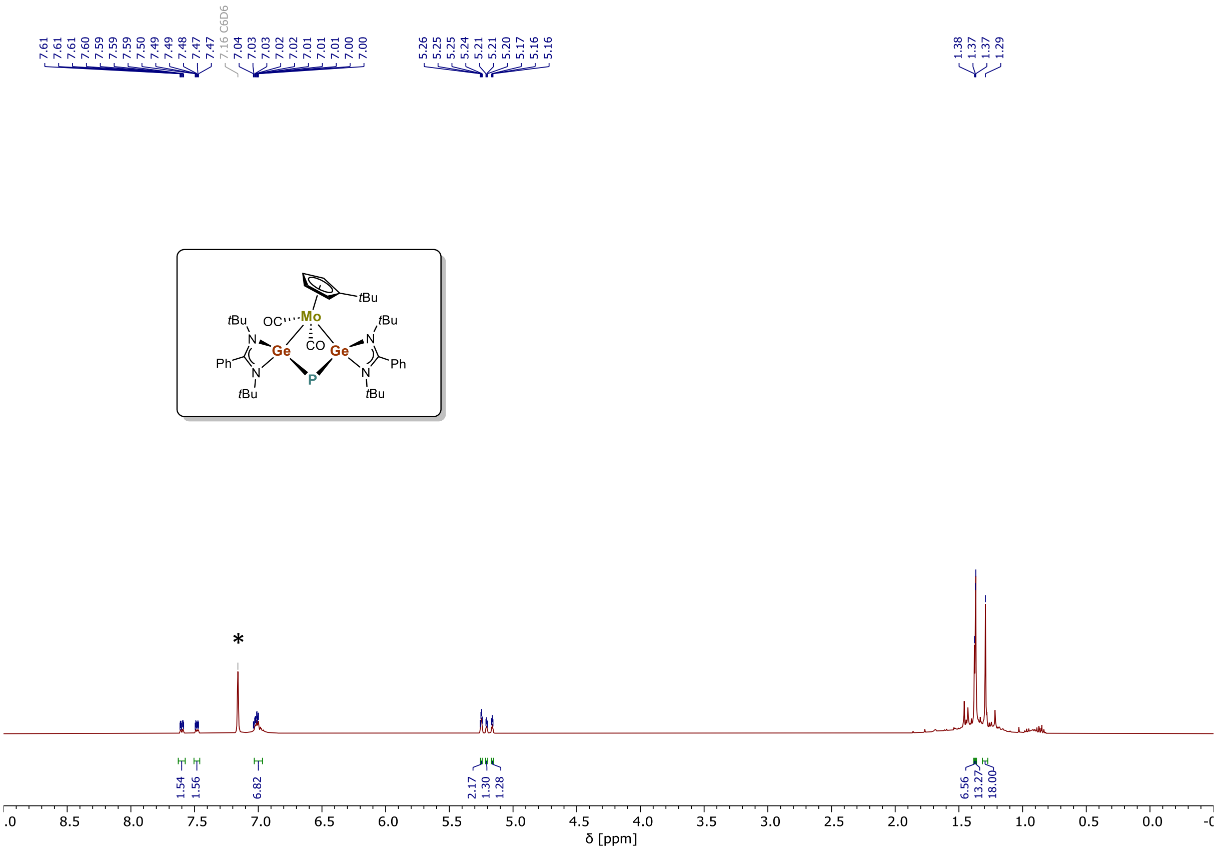

Figure S14. ^{1}H NMR spectrum of **5** in C_6D_6; * residual protio solvent signal.

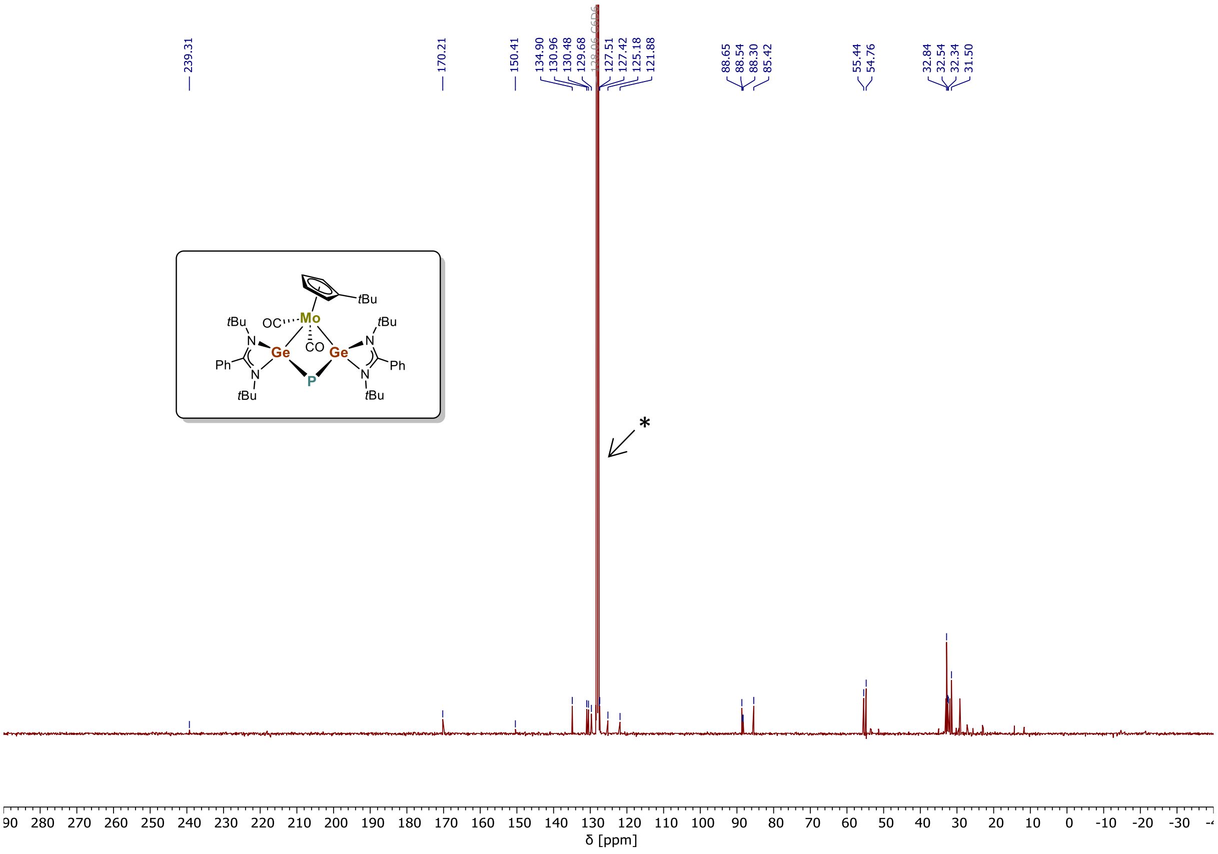

Figure S15. $^{13}C\{^{1}H\}$ NMR spectrum of **5** in C_6D_6; * C_6D_6.

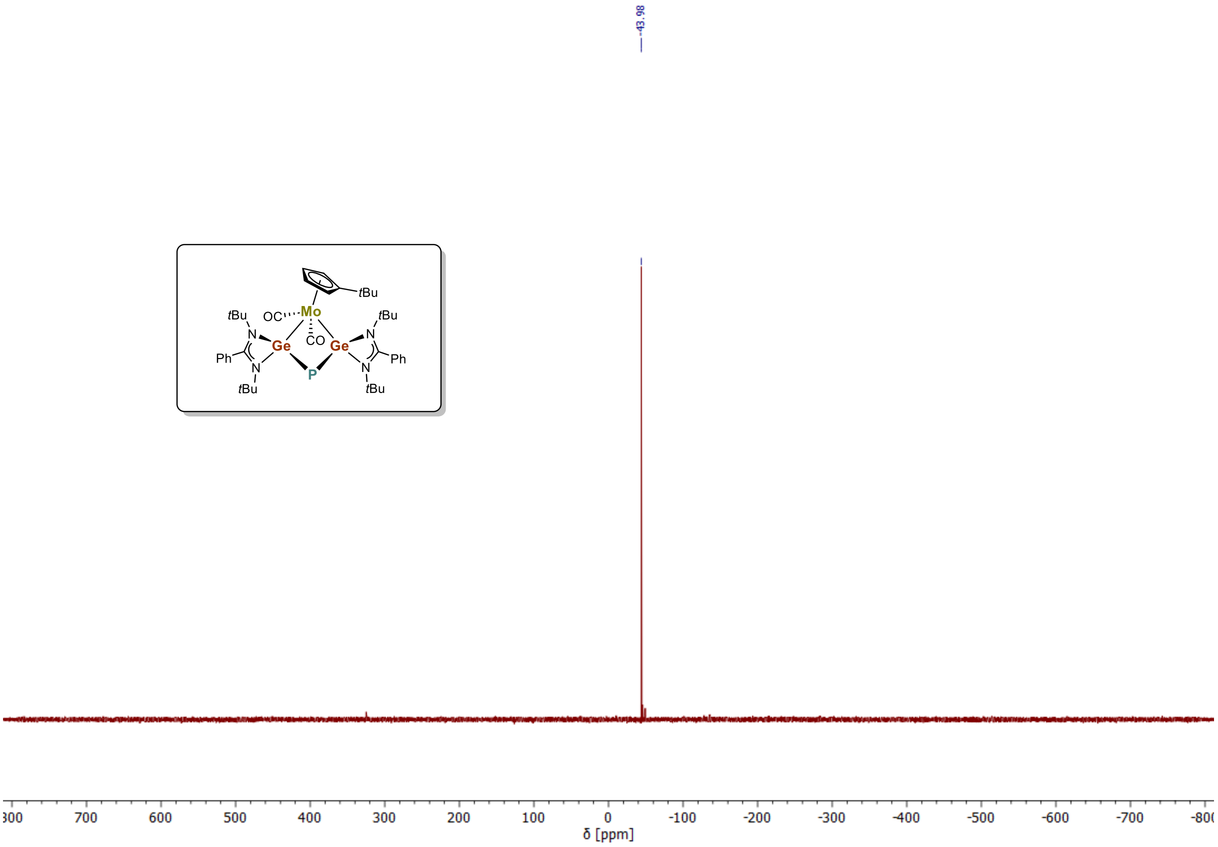

Figure S16. $^{31}P\{^{1}H\}$ NMR spectrum of **5** in C_6D_6.

II.5 NMR spectra of compound 6

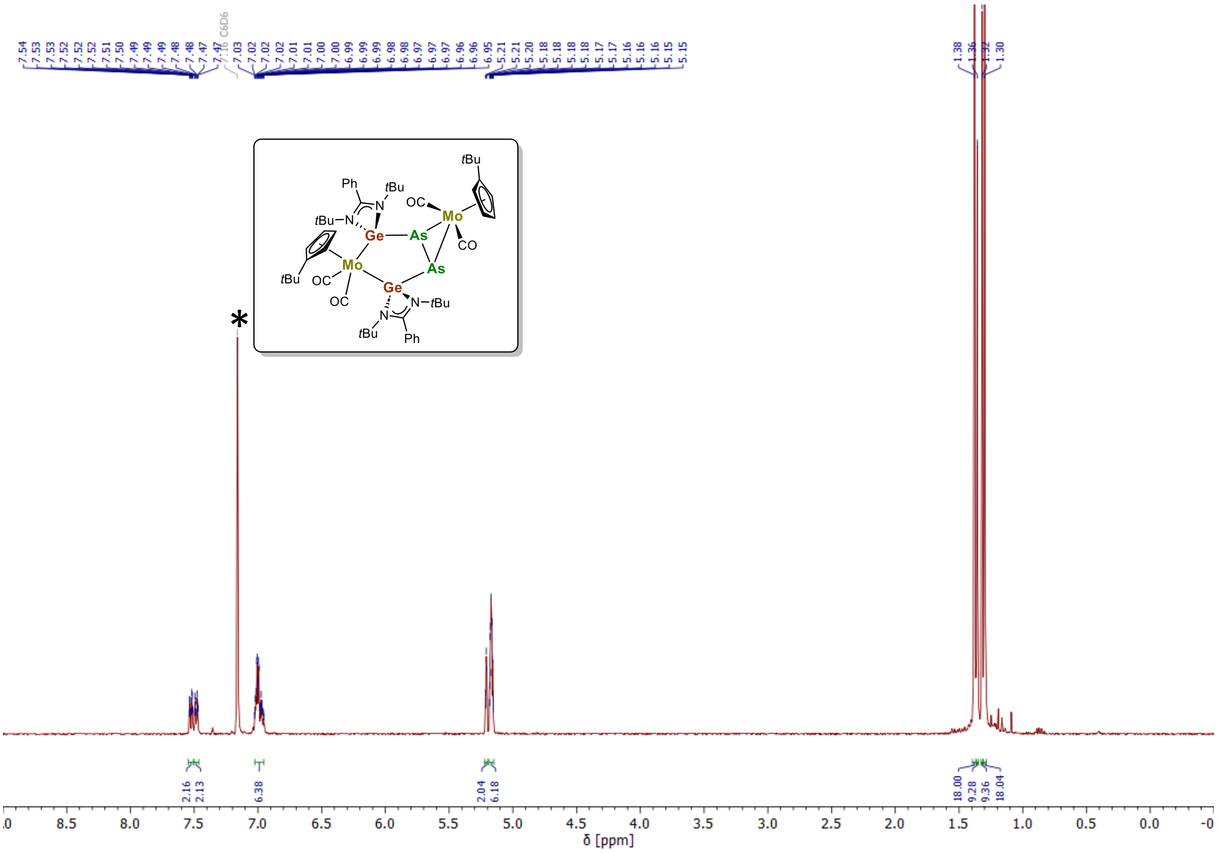

Figure S17. ^{1}H NMR spectrum of **6** in C_6D_6; * residual protio solvent signal.

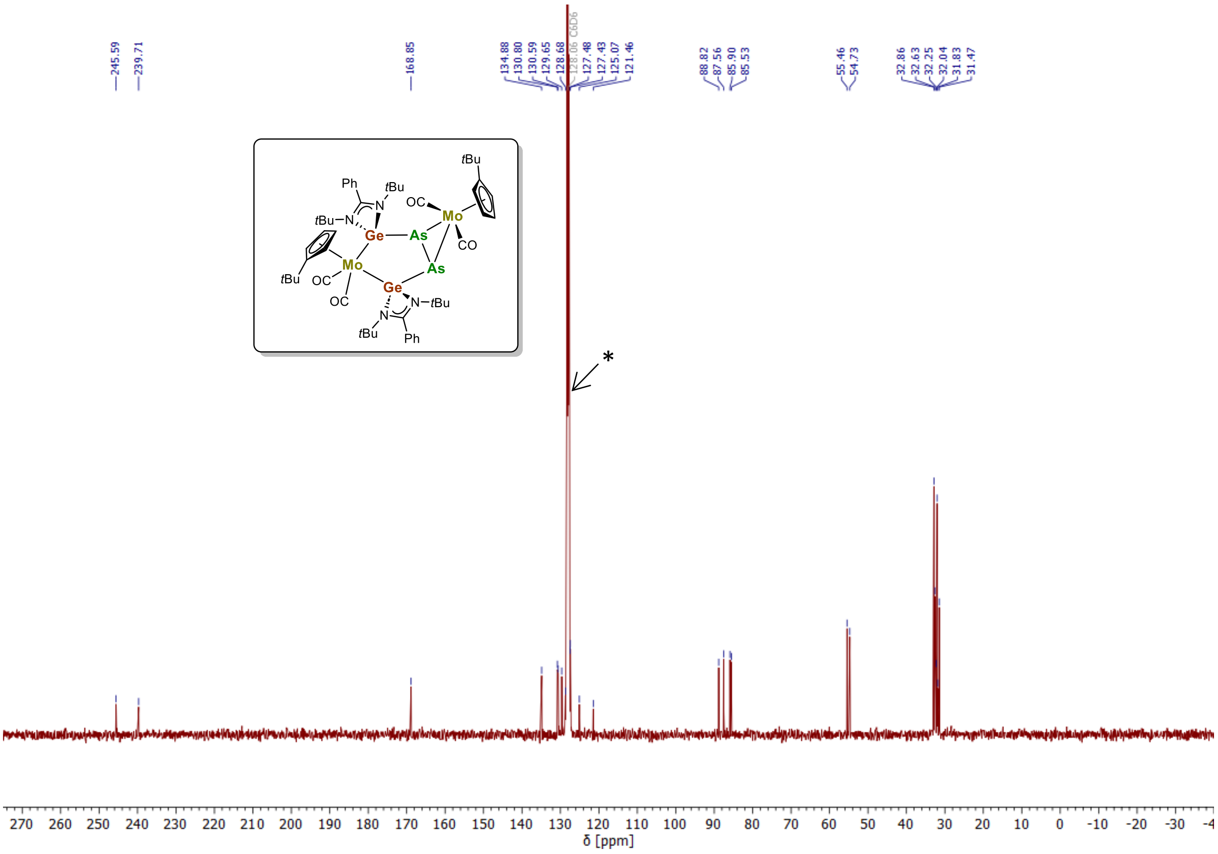

Figure S18. $^{13}C\{^{1}H\}$ NMR spectrum of **6** in C_6D_6; * C_6D_6.

II.6 NMR spectrum of compound 7

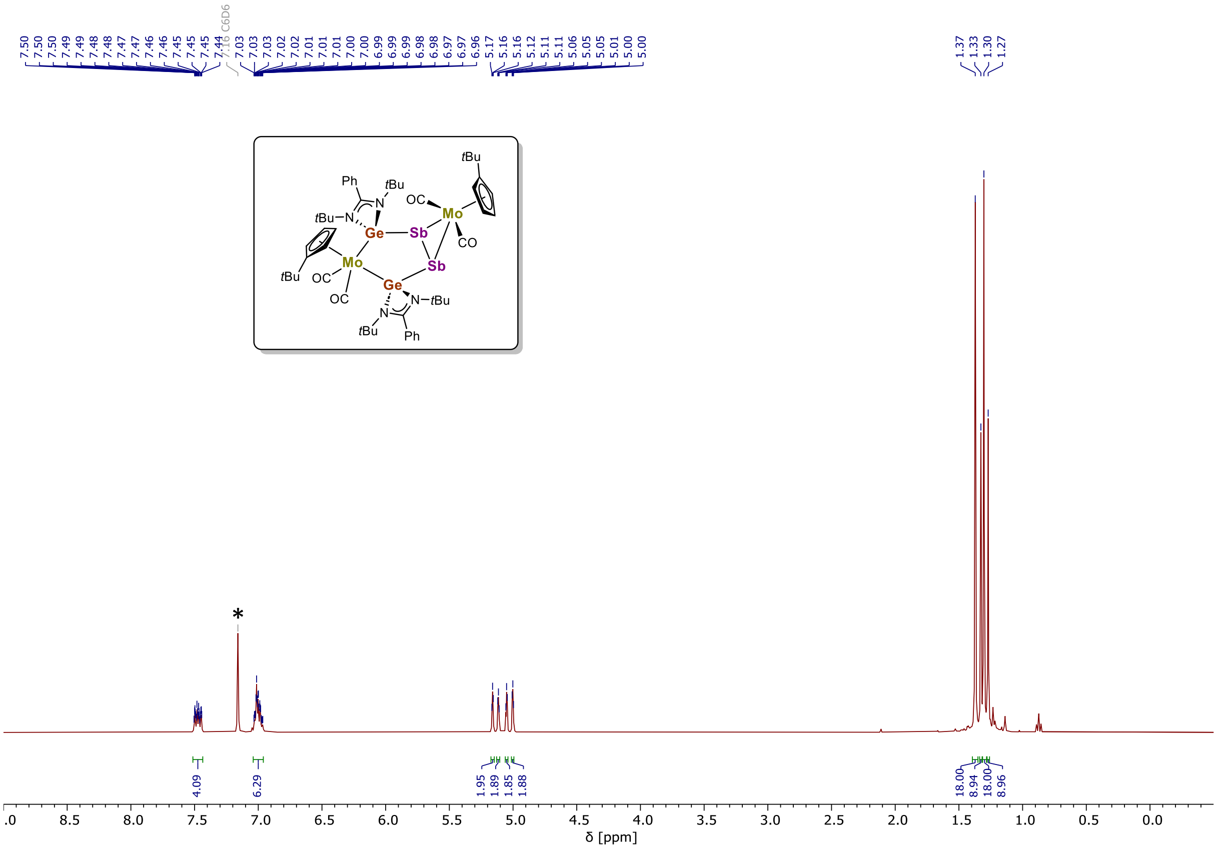

Figure S19. ^{1}H NMR spectrum of **7** in C_6D_6; * residual protio solvent signal.

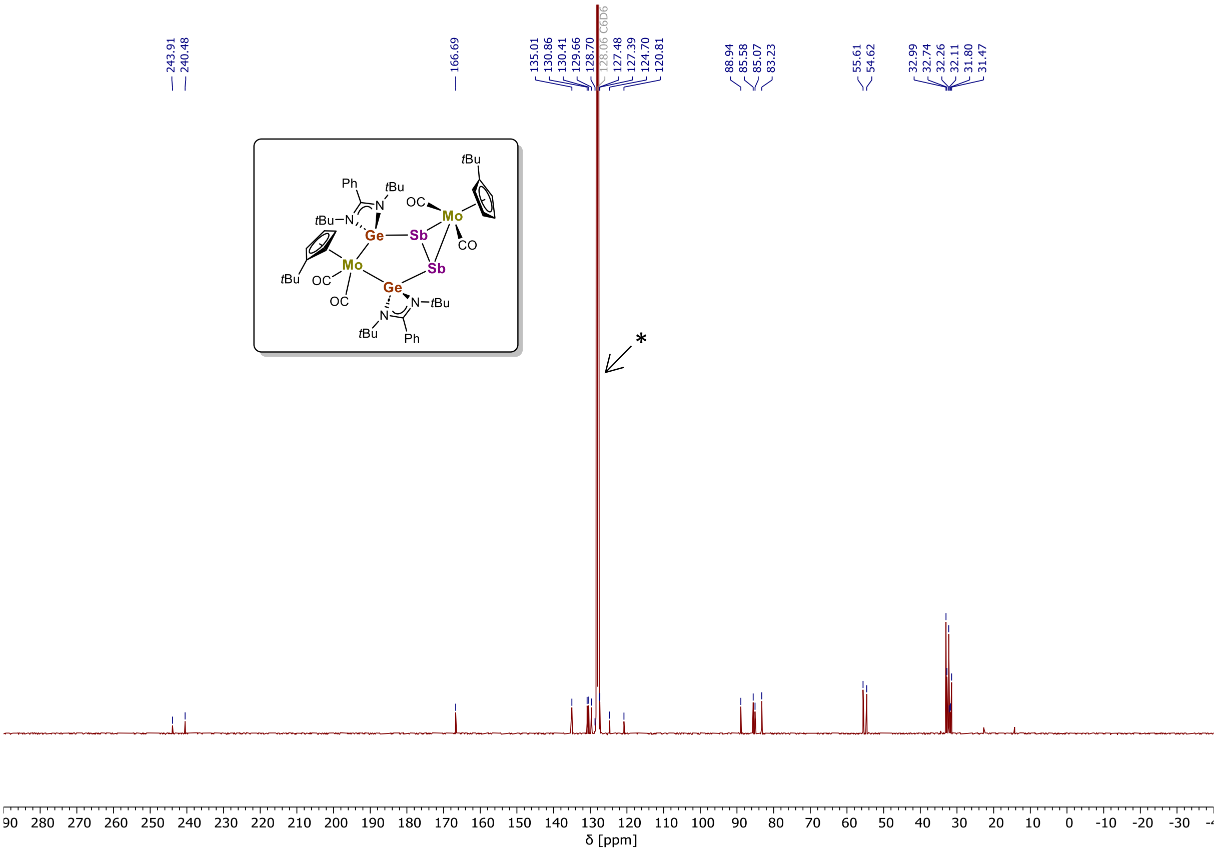

Figure S20. $^{13}C\{^{1}H\}$ NMR spectrum of **7** in C_6D_6; * C_6D_6.

III. IR spectra

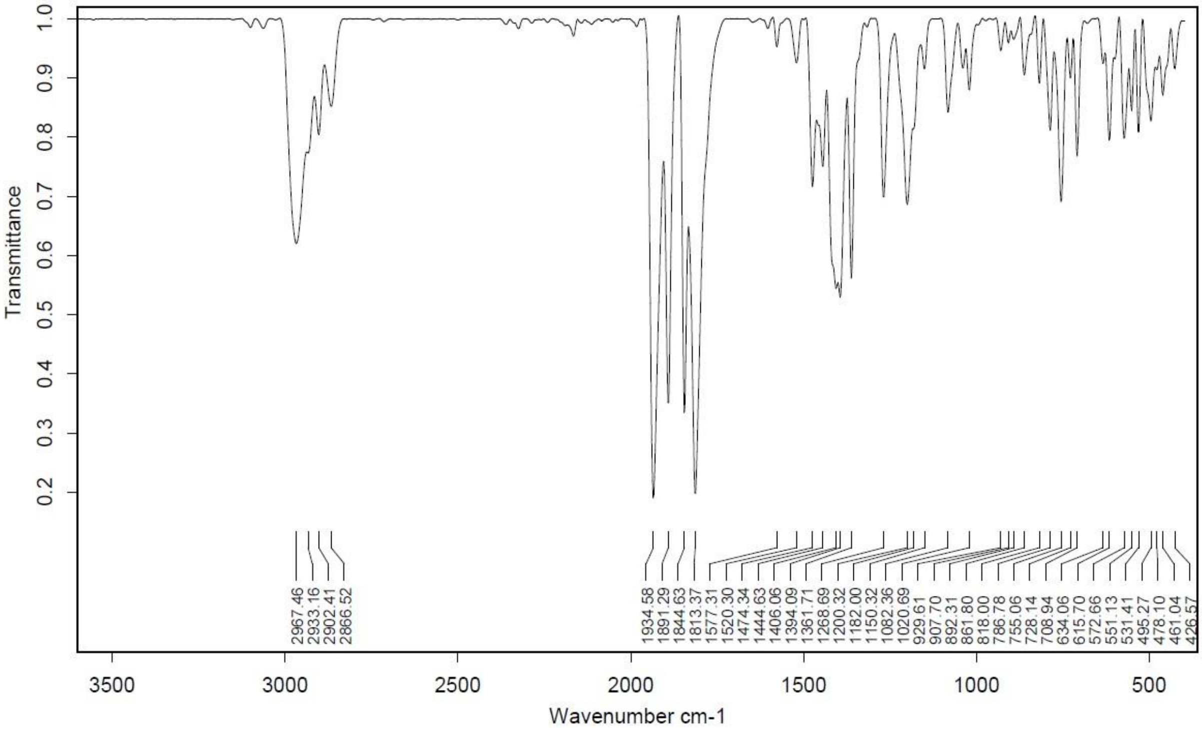

Figure S21: IR spectrum of compound **1**.

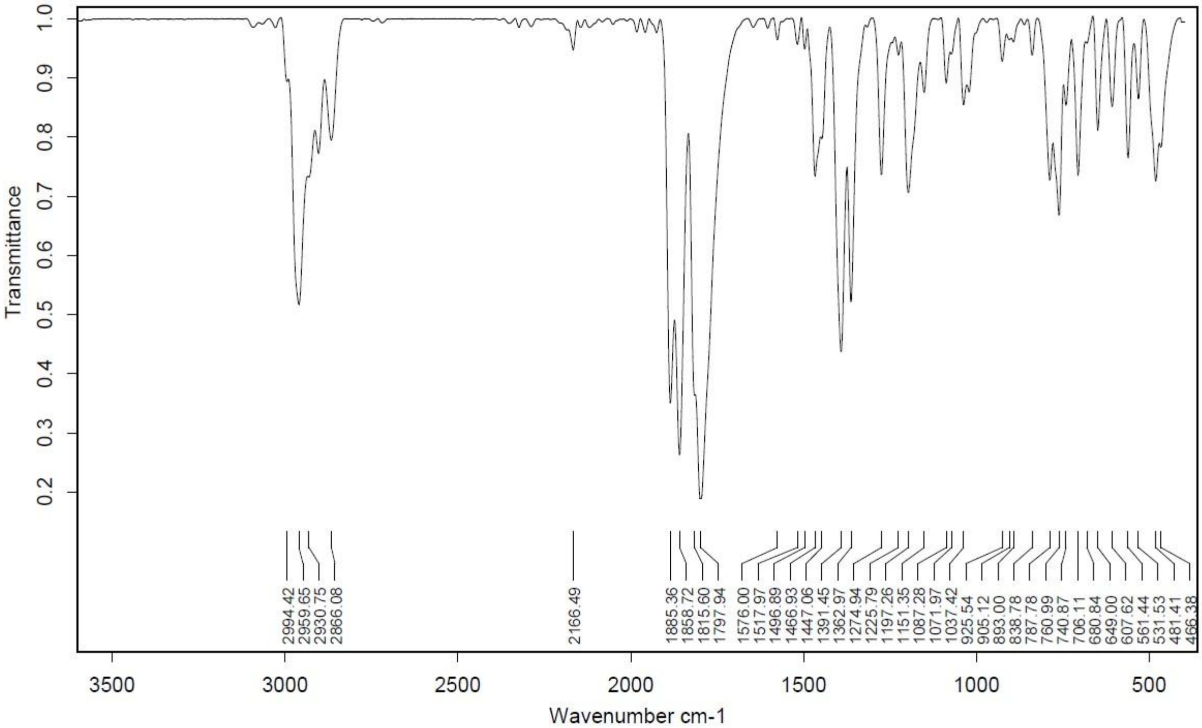

Figure S22: IR spectrum of compound **2**.

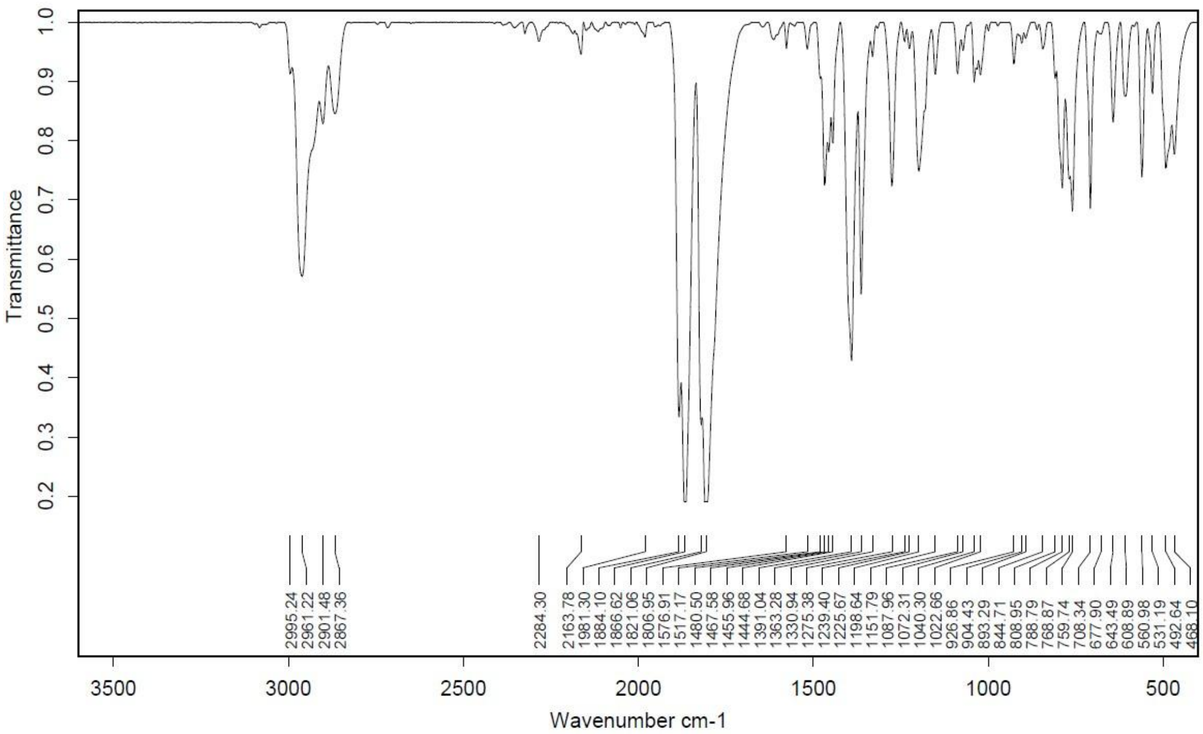

Figure S23: IR spectrum of compound **3**.

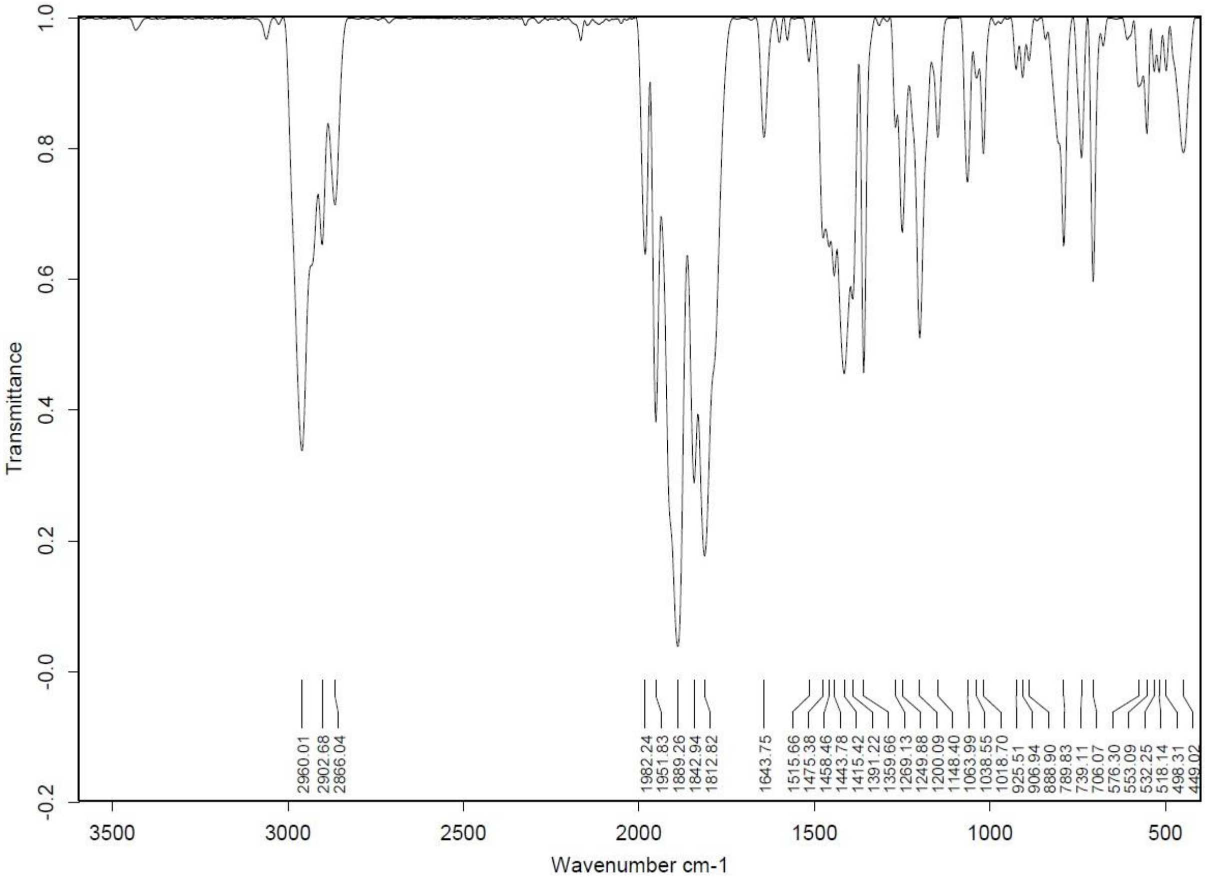

Figure S24. IR spectrum of compound **5**.

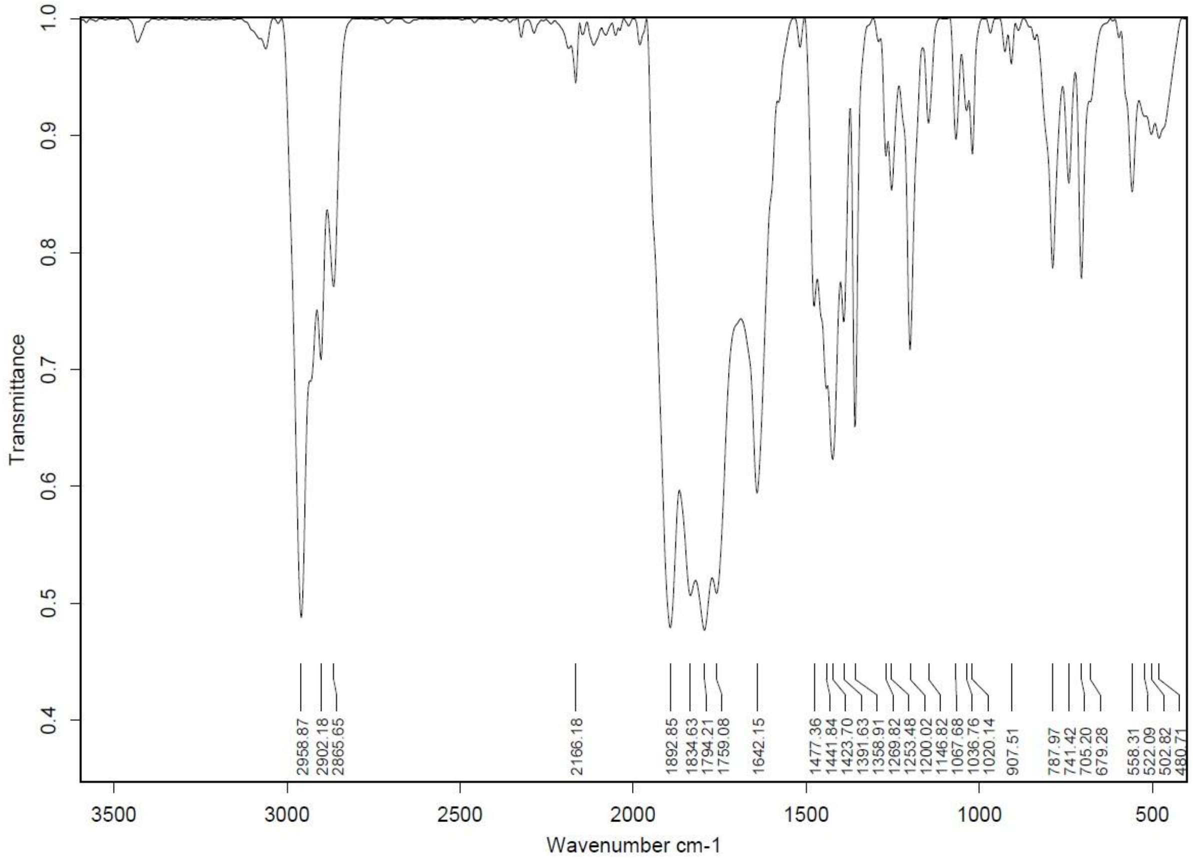

Figure S25. IR spectrum of compound **6**.

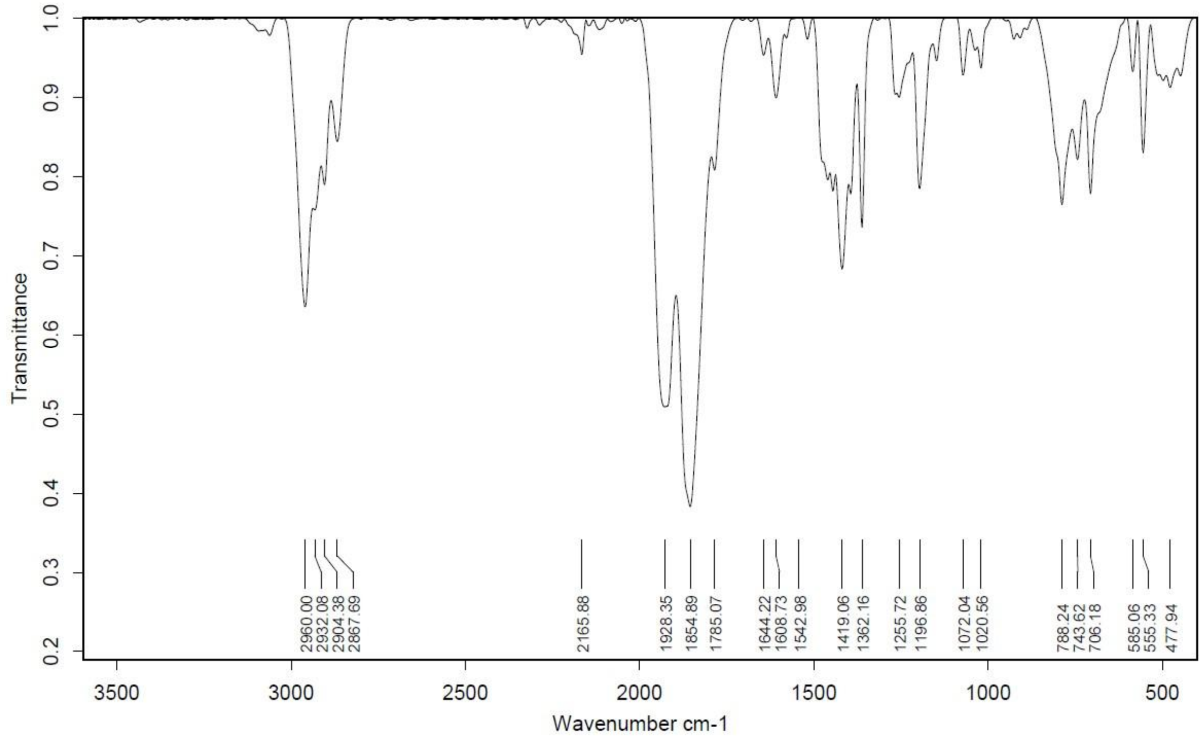

Figure S26. IR spectrum of compound **7**.

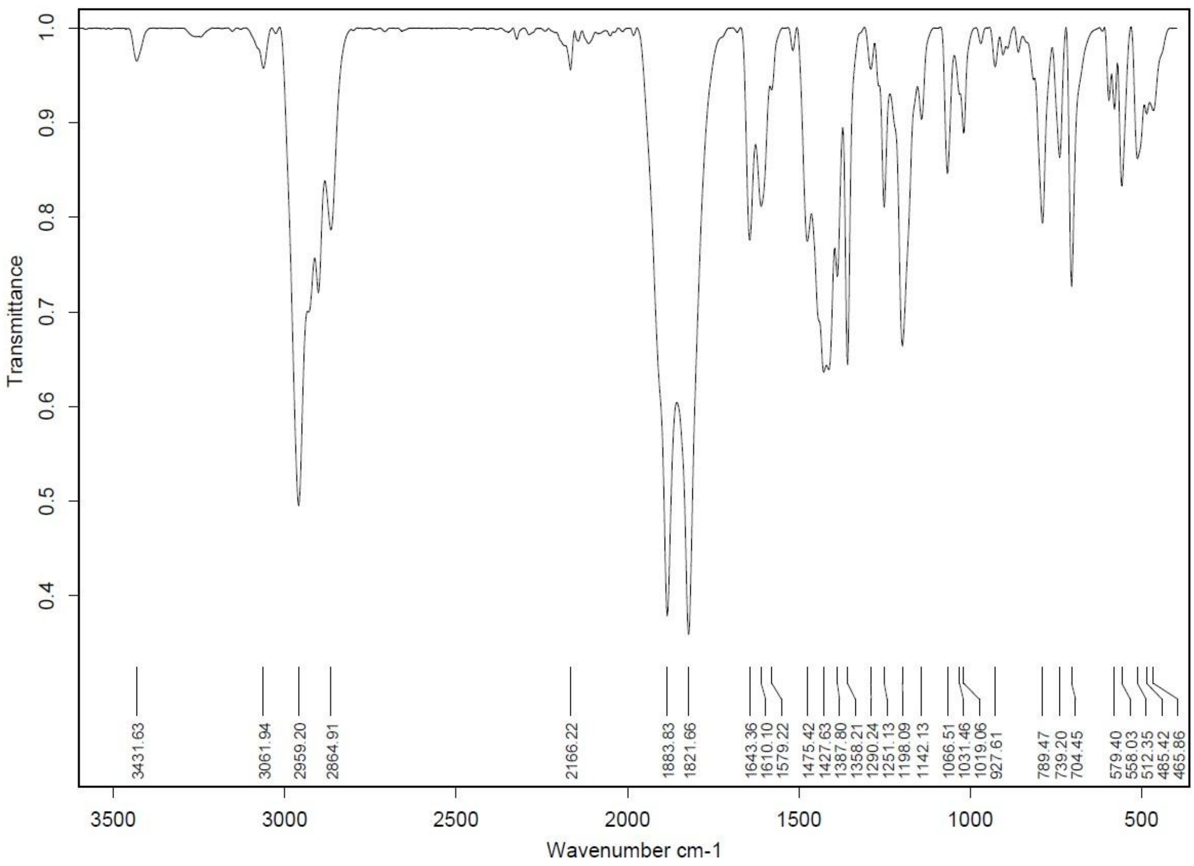

Figure S27. IR spectrum of compound **8**.

IV. Raman Microscopy

IV.1 General methods

Raman spectra were recorded on a *Bruker* Raman microscope Senterra II. A 532 nm laser with a power of 2 mW (compounds **1** and **2**) as well as 12 mW (compound **3**) was used for excitation and a detector unit with a resolution of 4 cm^{-1}. The samples consisted of selected single crystals that were previously prepared in a glove box. The samples reacted very sensitively to laser excitation; compound **4** could not even be measured.

IV.2 Discussion of the electronic situation in compound 1, 2 and 3

To evaluate the bonding properties within the compounds **1**, **2** and **3**, vibrational spectroscopic investigations using Raman spectroscopy were carried out. The spectroscopic measurements were accompanied by density functional theory (DFT) calculations. The thereby obtained local force constants nicely describe the situation of the potential curve close to the equilibrium distance between two atoms and thus are particularly suitable for estimating molecular bond strengths.[7] For additional validation, the results were compared with those obtained by quantum chemical calculations. Since, to our knowledge, no Mo–P force constants are known in the literature, the value in [$Mo(CO)_5PMe_3$] as an example of a typical Mo–P single bond was calculated for comparison (Table S1).

Although the measured Raman spectra (Figure S28-Figure S30) are of only average quality due to the sensitivity of the crystals, they are in good agreement with the calculated one (see Table S1 as well as Figure S28-Figure S30). The attribution of the bands to the motions succeeds by means of the investigations of the eigenvectors obtained from the frequency calculations.

Table S1. Assignments of the Raman bands of **1**, **2** and **3**.

	**1**		**2**		**3**	
	exp.	calc.	exp.	calc.	exp.	calc.
v(SiN_2)			759-790	766, 763	758-806	761, 763
v(SiN_2)			601	642, 637	601	633, 629
v(MoC)	505	511-612	508	504	500 (sh)	498-504
v(E-E)	-	-	283	225	144	161.0
v(Mo(ex)-E)	417	432	-	-	-	-
v(Mo-Si-E)			488	486		
v(E-Si)			428	404		
v(SiN_2) + v_s(SiE_2)	600	620				
v_{as}(SiE_2) + v_s(Si_2E)	576	570				
v_s(SiE_2)	464	466				
v_{as}(Si_2E) + v(Mo-Si)	ca. 513 (sh)	499				

In compound **1**, the terminal Mo–P stretching motion outside of the ring system is found at 417 cm^{-1}. Its energy is thus significantly increased compared to typical Mo–P single bonds, such as in [$Mo(CO)_5PR_3$] (205 (R = Me), 221 (Et), 171 cm^{-1} (Ph))[8]. This finding is supported by their rather different bond lengths (d(P1–Mo1) 2.5412(12) and d(Mo2–P1) 2.324(2) Å). In the range between about 408 and about 600 cm^{-1} vibrations are detected, which can be assigned to the Mo–C bonds of the Mo-carbonyl systems (505 cm^{-1}), the SiN_2 (600 cm^{-1}), as well as of the $MoSi_2P_2$ ring system (464, 513, 576 cm^{-1}). However, assignments are complicated due to the coupling of the vibrational motions of these molecular groups, as the comparison of the experimental with the theoretical spectrum shows. On the basis of theoretical calculations, a local force constant of 1.194 mdyn $Å^{-1}$ for the Mo1–P1 bond within

the heterocycle and a value of 2.189 mdyn Å^{-1} for the *exo*-Mo2–P1 bond are determined. Within the reference molecule [Mo(CO)$_5$PMe$_3$], the respective experimental vibrational frequency was determined to be 205 cm^{-1} [8], the theoretical value was calculated to be 188 cm^{-1}, its theoretical local force constant is 1.155 mdyn Å^{-1}. From these findings we conclude that the bond between Mo2 and P1 is best described as double bond, which is also supported by the rather divergent bond lengths of P1 towards both Mo atoms (P1–Mo1 2.5412(12) Å and P1–Mo2 2.324(2) Å). Significant differences are also observed within the derived local force constant values of the three Si–P bonds: The smallest value is 1.53 (Si1–P1), followed by 1.84 (Si2–P2) and 2.42 mdyn Å^{-1} (Si1–P2) with the latter local force constant being in line with a double bond for Si1–P2. Additionally, the isosurface plots of the localized molecular orbitals (MOs) describing the Si–P and Mo–P π-bonds as well as of the Si–Mo dative bond are depicted in Figure 3.

In compound **2**, the most intense band in the Raman spectrum (exp. 283, calc. 225 cm^{-1}) can be assigned to the As–As valence vibration. At 428 and 488 cm^{-1} (calc. 404-398 or 486 cm^{-1}) the signals of the MoSiAs three-membered rings are found, which are predominantly localized at silicon due to the lowest atomic mass of this atom. Bands are found at 759-790 cm^{-1} (broad and weak) and 601 cm^{-1} (sharp), which are assigned to the SiN$_2$ motion and are coupled with deformation motions of the phenyl groups (calc. 766, 763 and 642, 637 cm^{-1}). An intense signal at about 508 cm^{-1} (calc. 498-505 cm^{-1}) can be assigned to the Mo–C stretching vibrations of the Mo-carbonyl groups. For **2**, the theoretical local force constant value of the inter-pnictogen bond amounts to 0.94 mdyn Å^{-1} and is thus in good agreement with a weak As–As single bond compared to that in yellow arsenic (As$_4$, 1.674 mdyn Å^{-1}).[9]

In compound **3**, the most intense Raman band is observed at 144 cm^{-1} (calc. 161 cm^{-1}). According to expected results, it can be clearly assigned to the Sb–Sb stretching mode. The signal at 106 cm^{-1} is probably consistent with deformation or lattice vibrations. The other signals are only weak and broad. In agreement with **1** and **2**, broad and weak signals at 760 cm^{-1} (calc. 761, 763 cm^{-1}) and about 601 cm^{-1} (very weak) (calc. 629, 633 cm^{-1}) are attributed to SiN$_2$ vibrations and another one at about 500 cm^{-1} (calc. 498-504 cm^{-1}) to Mo–C stretching modes of the Mo carbonyl groups. Relative to its isostructural diarsine compound **2**, a comparable situation is found for the Sb-Sb bond in **3**. A local mode force constant value of 0.779 mdyn Å^{-1} is found, which is also in agreement with a weak Sb–Sb single bond after comparison to the single bond in the theoretically isostructural antimony tetrahedra (Sb$_4$, 1.224 mdyn Å^{-1}).[9]

Table S2. Local mode force constant values (given in mdyn Å^{-1}) of selected bonds in **1**, **2** and **3**.

Local mode force constant f	**1** (E = P)	**2** (E = As)	**3** (E = Sb)
E-E	-	0.940	0.779
Si-E	1.841/2.421/1.529	1.945	1.414
Mo-E	1.194	0.647	0.595
Mo(exo)-E	2.189		
Si-Mo	1.366	1.840	1.644

IV.3 Discussion of the stability of the different ring sizes in compound 1, 2 and 3

As we questioned, why for phosphorous the five-membered metallacycle is obtained compared to the smaller three-membered heterocycles for the heavier homologue arsenic and antimony, the energy differences between the five-membered-metallacycle (as realized in **1**) and the structural motifs with two three-membered rings connected by pnictogen–pnictogen single

bonds (as realized in **2** and **3**) were calculated by means of DFT calculations. The calculations confirm the thermodynamic stability of the experimental structures of **1** (68 kJ mol^{-1} more stable than the hypothetical three-membered P ring structure) as well as of **3** (33 kJ mol^{-1} more stable than the analogous five-membered ring compound). In contrast, the energy difference in the case of arsenic is quite small and theoretically determined to be 14 kJ mol^{-1} more favourable to the five-membered ring compound, whereas the slightly less stable three-membered ring with an As–As single bond is found experimentally in the solid state.

IV.4 Discussion of the electronic situation in compound I-P, I-As and 4

Unfortunately, we were not able to detect the Raman spectrum of **4** due to its high sensitivity even to low laser irradiation. Therefore, we focused on the results of the theoretical calculations.

Table S3. Local mode force constant values f (given in mdyn $Å^{-1}$) of selected bonds in **4** and its phosphorus and arsenic homologues as well as Mulliken atomic charges q of Si and Pn (given in e) and NICS values.

		**I-P** (Si_2P_2)	**I-As** (Si_2As_2)	**4** (Si_2Sb_2)
Local mode force constant values	f(Si–E)	2.01	1.77	1.34
	f(Si–N)	1.67	1.67	1.64
Mulliken atomic charges	q(Si)	+0.41	+0.39	+0.30
	q(E)	−0.39	−0.37	−0.31
NICS values	NICS(0)	−7.51	−6.85	−5.77
	NICS(1)	−3.02	−2.74	−2.34
	NICS(2)	0.31	0.53	0.93

IV.5 Discussion of the electronic situation in compounds 2 and 6 (As-As bonds) as well as 3, 7, and 8 (Sb-Sb bonds)

Table S4. Theoretical results: Wiberg Bond Index (WBI) (Natural Bond Order (NBO) analysis), local force constant values and distances of the molecules containing As–As or Sb–Sb bonds.

		WBI	local force constant (given in mdyn Å^{-1})	d(Pn–Pn) (given in Å)
As_4	As–As		1.674*	
**2**	As–As	0.880	0.940	2.491
**6**	As–As	1.049	1.710	2.379
Sb_4	Sb–Sb		1.224*	
**3**	Sb–Sb	0.917	0.779	2.846
**7**	Sb–Sb	1.034	1.364	2.709
**8**	Sb2–Sb1	0.890	0.959	2.825
	Sb1–Sb1‘	0.880	0.951	2.820

* The experimental force constant values of As_4 and Sb_4 are given as references (see text).

IV.6 Raman spectra

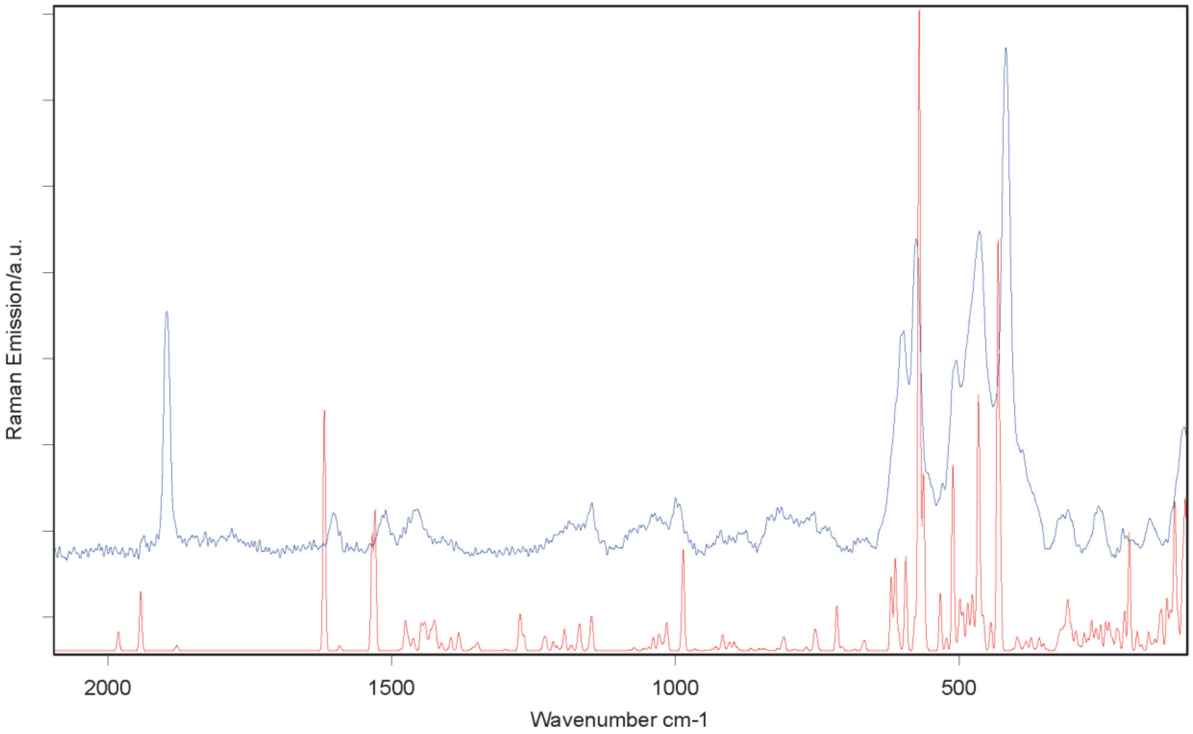

Figure S28. Raman spectrum of 1 at an excitation wavelength of 532 nm.

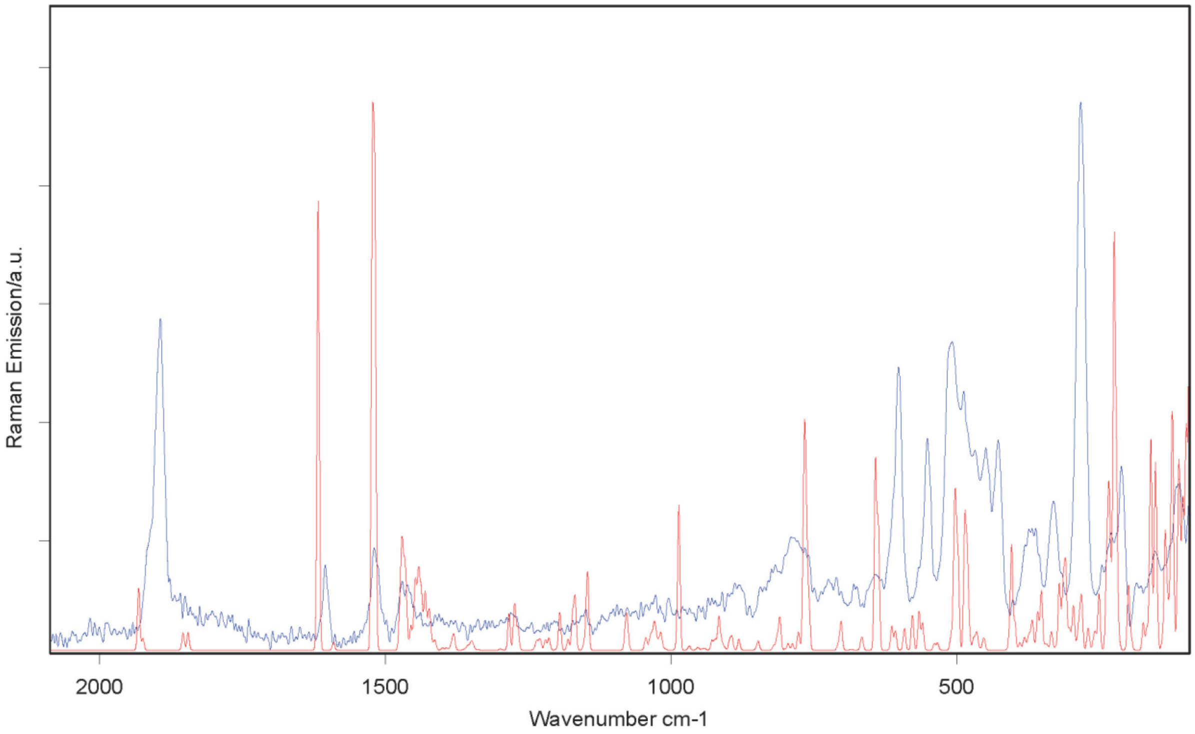

Figure S29. Raman spectrum of **2** at an excitation wavelength of 532 nm.

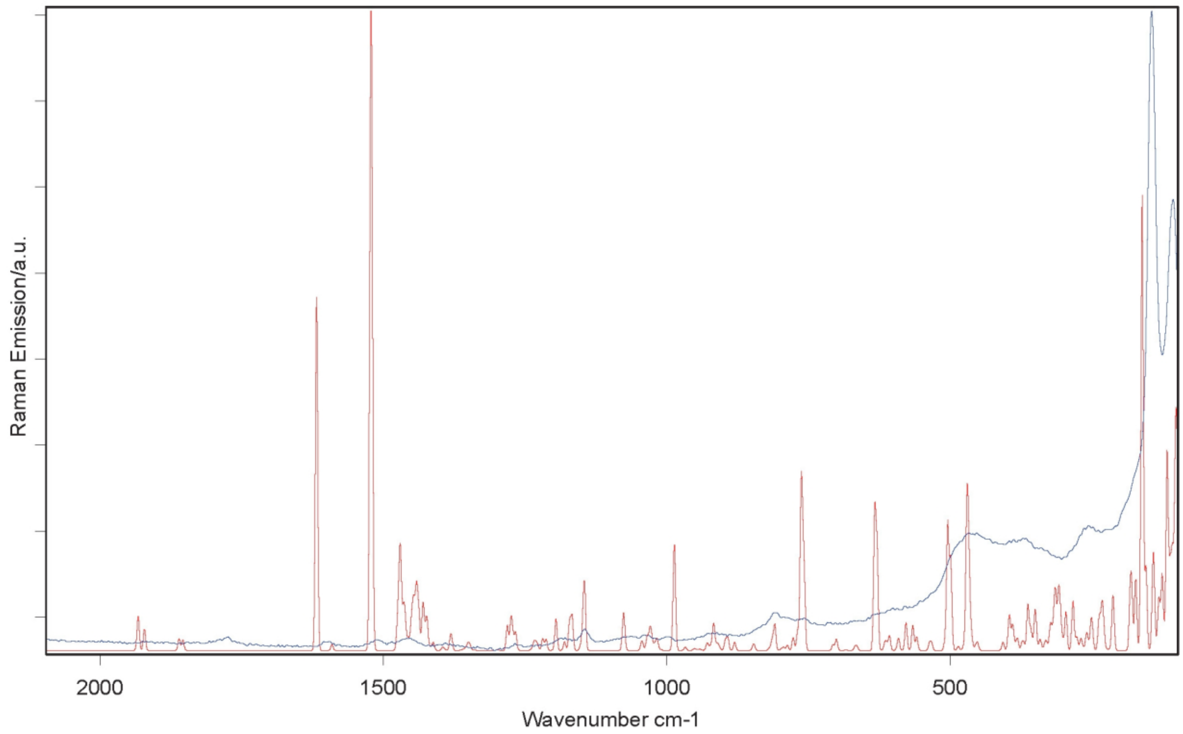

Figure S30. Raman spectrum of **3** at an excitation wavelength of 532 nm.

V. X-ray crystallography

V.1 General methods

Suitable crystals for the X-ray analysis of all compounds were obtained as described above. A suitable crystal was covered in mineral oil (Aldrich) and mounted on a glass fibre. The crystal was transferred directly to the cold stream of a STOE StadiVari (100 K, 110 K or 120 K) diffractometer. All structures were solved by using the program SHELXS/T[10,11] and Olex2.[12] The remaining non-hydrogen atoms were located from successive difference Fourier map calculations. The refinements were carried out by using full-matrix least-squares techniques on F_o^2 by using the program SHELXL.[10,11] The H-atoms were introduced into the geometrically calculated positions (SHELXL procedures) unless otherwise stated and refined riding on the corresponding parent atoms. In each case, the locations of the largest peaks in the final difference Fourier map calculations, as well as the magnitude of the residual electron densities, were of no chemical significance. Specific comments for each data set are given below. Summary of the crystal data, data collection and refinement for compounds are given in Table S5 and Table S6.

Crystallographic data for the structures reported in this paper have been deposited with the Cambridge Crystallographic Data Centre as a supplementary publication no. CCDC 2488338-2488344 and 2488347. Copies of the data can be obtained free of charge on application to CCDC, 12 Union Road, Cambridge CB21EZ, UK (fax: (+(44)1223-336-033; email: deposit@ccdc.cam.ac.uk).

The following special comments were applied to the models of the structures:

In the crystal structure of **2**, one co-crystallized toluene molecule (C53-C59) is disordered over two positions with an occupancy ratio of 0.5/0.5.

In the crystal structure of **4**, one of the *t*Bu groups (C13–C15) is disordered over two positions with an occupancy ratio of 0.35/0.65.

In the crystal structure of **5**, three of the *t*Bu groups (C16–C19, C27–C30 and C36–C39) are disordered over two positions with an occupancy ratio of 0.30/0.70, 0.64/0.36 and 0.40/0.60.

V.2 Summary of crystal data

Table S5. Crystal data, data collection and refinement for compounds 1-4.

**Compound**	**1**	**2·(toluene)**	**3·(C_6D_6)**	**4**
Formula	$C_{52}H_{72}Mo_2N_4O_4P_2Si_2$	$C_{52}H_{72}As_2Mo_2N_4O_4Si_2$ ·($C_6H_5CH_3$)	$C_{52}H_{72}Mo_2N_4O_4Sb_2Si_2$ ·(C_6D_6)	$C_{30}H_{46}N_4Sb_2Si_2$
$D_{calc.}$/ g cm^{-3}	1.397	1.457	1.558	1.484
μ/mm^{-1}	0.619	1.611	1.400	1.678
Formula Weight	1127.13	1307.17	1392.84	762.39
Colour	violet	orange	red	red
Shape	plate-shaped	fragment-shaped	plate-shaped	block-shaped
Size/mm^3	0.11×0.07×0.03	0.20×0.13×0.06	0.16×0.09×0.04	0.20×0.14×0.06
T/K	100	100	100	110
Crystal System	triclinic	triclinic	triclinic	orthorhombic
Flack Parameter	-	-	-	0.51(6)
Hooft Parameter	-	-	-	-0.019(10)
Space Group	$P\bar{1}$	$P\bar{1}$	$P\bar{1}$	$Pca2_1$
a/Å	13.381(9)	12.3927(5)	12.6121(10)	17.2948(4)
b/Å	13.747(5)	12.7317(6)	12.7124(14)	9.2045(2)
c/Å	16.990(9)	20.4434(8)	20.358(2)	21.4327(7)
α/°	78.34(4)	72.769(3)	74.858(8)	
β/°	80.78(5)	79.298(3)	72.104(7)	
γ/°	61.42(4)	77.211(3)	77.893(8)	
V/Å^3	2680(3)	2979.3(2)	2968.3(5)	3411.87(16)
Z	2	2	2	4
Z'	1	1	1	1
Wavelength/Å	0.71073	0.71073	0.71073	0.71073
Radiation type	Mo K_a	Mo K_a	Mo K_a	Mo K_a
Θ_{min}/°	1.944	1.746	1.763	1.900
Θ_{max}/°	25.998	27.000	30.113	28.000
Measured Refl's.	34265	32127	38883	22792
Indep't Refl's	10507	12787	14531	7858
Refl's I≥2 σ(I)	7451	10026	11139	6730
R_{int}	0.0472	0.0273	0.0265	0.0451
Parameters	613	717	667	387
Restraints	0	237	0	64
Largest Peak	0.469	0.363	0.572	1.847
Deepest Hole	-0.602	-0.463	-0.894	-1.622
GooF	0.969	0.988	0.958	1.029
wR_2 (all data)	0.0805	0.0620	0.0600	0.1781
wR_2	0.0714	0.0595	0.0562	0.1672
R_1 (all data)	0.0682	0.0408	0.0452	0.0794
R_1	0.0358	0.0266	0.0270	0.0677

Table S6. Crystal data, data collection and refinement for compounds 5-8.

Compound	5·(C_6D_6)	6	7·1.5(THF)	8·5(THF)
Formula	$C_{41}H_{59}Ge_2MoN_4O_2P$ ·(C_6D_6)	$C_{52}H_{72}As_2Ge_2Mo_2N_4O_4$	$C_{52}H_{72}As_2Ge_2Mo_2N_4O_4$ ·1.5(C_4H_8O)	$C_{82}H_{118}Ge_4Mo_2N_8O_4Sb_4$ ·5(C_4H_8O)
$D_{calc.}$/ g cm^{-3}	1.384	1.611	1.612	1.566
μ/mm^{-1}	1.581	2.831	2.255	2.303
Formula Weight	996.15	1304.03	1505.85	2609.59
Colour	orange	red	red	red
Shape	block-shaped	plate-shaped	rod-shaped	fragment-shaped
Size/mm^3	0.15×0.10×0.07	0.14×0.10×0.05	0.28×0.10×0.10	0.35×0.21×0.07
T/K	120	110	110	110
Crystal System	triclinic	monoclinic	triclinic	triclinic
Flack Parameter	-	-	-	-
Hooft Parameter	-	-	-	-
Space Group	$P\bar{1}$	$P2_1/c$	$P\bar{1}$	$P\bar{1}$
a/Å	13.030(3)	13.4250(5)	12.1886(6)	13.000(3)
b/Å	13.853(3)	33.9100(9)	16.4043(9)	15.880(3)
c/Å	16.350(3)	11.8193(5)	16.5887(9)	15.960(3)
α/°	86.03(3)		73.331(4)	110.70(3)
β/°	67.70(3)	92.271(3)	77.730(4)	112.40(3)
γ/°	62.03(3)		88.912(4)	94.10(3)
V/Å^3	2390.2(11)	5376.4(3)	3101.7(3)	2767.4(12)
Z	2	4	2	1
Z'	1	1	1	0.5
Wavelength/Å	0.71073	0.71073	0.71073	0.71073
Radiation type	Mo K_a	Mo K_a	Mo K_a	Mo K_a
Θ_{min}/°	1.851	1.826	1.919	1.743
Θ_{max}/°	30.123	25.998	25.999	28.969
Measured Refl's.	30920	28440	29556	30918
Indep't Refl's	11728	10407	12146	12643
Refl's I≥2 σ(I)	8375	6322	9826	9993
R_{int}	0.0282	0.0529	0.0619	0.0219
Parameters	680	613	749	574
Restraints	540	12	364	0
Largest Peak	1.761	2.377	1.688	0.666
Deepest Hole	-1.861	-1.006	-2.404	-0.731
GooF	1.057	0.940	1.012	0.967
wR_2 (all data)	0.1607	0.1086	0.1581	0.0522
wR_2	0.1448	0.0980	0.1537	0.0509
R_1 (all data)	0.0832	0.0982	0.0714	0.0337
R_1	0.0541	0.0490	0.0627	0.0234

V.3 Crystal structures

V.3.1 Crystal structure of compound 1

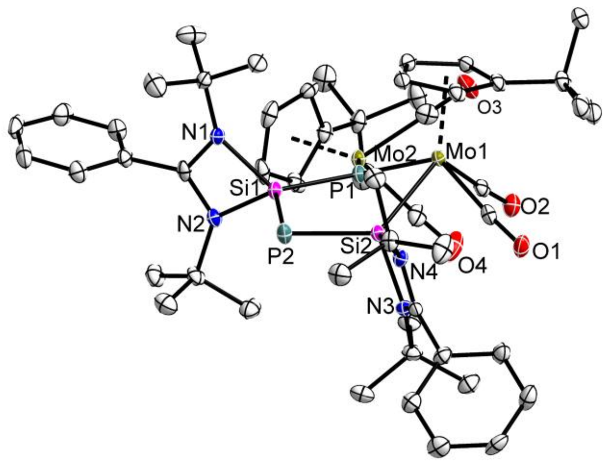

Figure S31: Molecular structure of the compound **1** in the solid state with thermal ellipsoids at 50% level. Hydrogen atoms are omitted for clarity. Selected bond distances [Å] and angles [°]: Mo1–P1 2.5412(12), Mo1–Si2 2.519(2), Mo2–P1 2.324(2), P1–Si1 2.283(2), P2–Si1 2.123(2), P2–Si2 2.199(2); Si2–Mo1–P1 76.84(5), Mo2–P1–Mo1 129.63(5), Si1–P1–Mo1 110.26(6), Si1–P1–Mo2 119.19(6), Si1–P2–Si2 92.53(7), P2–Si1–P1 113.78(7), P2–Si2–Mo1 126.58(6).

V.3.2 Crystal structure of compound 2

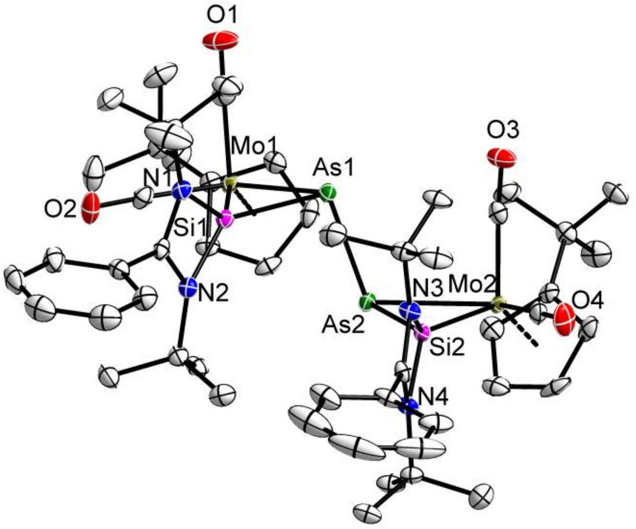

Figure S32: Molecular structure of the compound **2** in the solid state with thermal ellipsoids at 50% level. Hydrogen atoms and non-coordinating solvent molecules are omitted for clarity. Selected bond distances [Å] and angles [°]: Mo1–As1 2.7528(3), Mo1–Si1 2.4039(6), Mo2–As2 2.7351(3), Mo2–Si2 2.3943(6), As1–As2 2.4905(3), As1–Si1 2.2677(6), As2–Si2 2.2602(6); Si1–Mo1–As1 51.63(2), Si2–Mo2–As2 51.768(15), As2–As1–Mo1 100.331(11), Si1–As1–Mo1 56.22(2), Si1–As1–As2 97.56(2), As1–As2–Mo2 109.838(11), Si2–As2–Mo2 56.31(2), Si2–As2–As1 105.73(2), As1–Si1–Mo1 72.14(2), As2–Si2–Mo2 71.91(2).

V.3.3 Crystal structure of compound 3

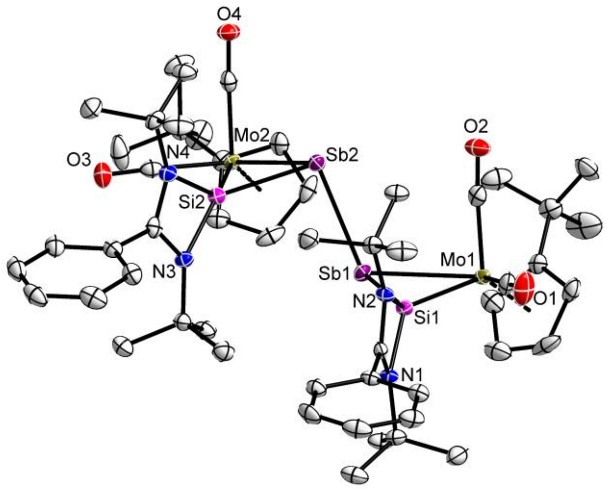

Figure S33. Molecular structure of the compound **3** in the solid state with thermal ellipsoids at 50% level. Hydrogen atoms and non-coordinating solvent molecules are omitted for clarity. Selected bond distances [Å] and angles [°]: Mo1–Sb1 2.9077(4), Mo1–Si1 2.4138(7), Mo2–Sb2 2.9306(4), Mo2–Si2 2.4195(7), N1–Si1 1.830(2), N2–Si1 1.822(2), N3–Si2 1.823(2), N4–Si2 1.822(2), Sb1–Sb2 2.8456(4), Sb1–Si1 2.4621(7), Sb2–Si2 2.4763(7); Si1–Mo1–Sb1 54.16(2), Si2–Mo2–Sb2 54.12(2), Sb2–Sb1–Mo1 110.286(12), Si1–Sb1–Mo1 52.63(2), Si1–Sb1–Sb2 106.36(2), Sb1–Sb2–Mo2 96.670(12), Si2–Sb2–Mo2 52.35(2), Si2–Sb2–Sb1 94.83(2), Mo1–Si1–Sb1 73.21(2), Mo2–Si2–Sb2 73.53(2).

V.3.4 Crystal structure of compound 4

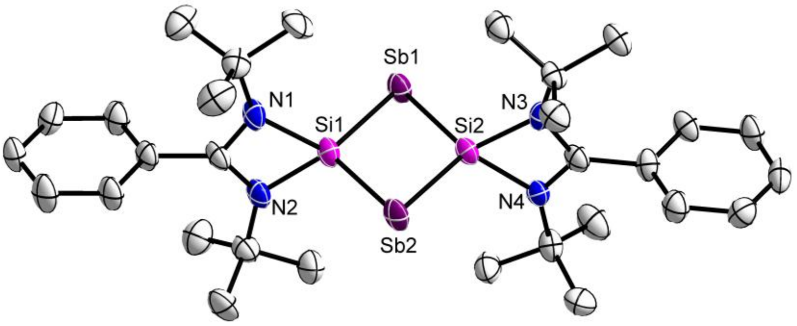

Figure S34. Molecular structure of the compound **4** in the solid state with thermal ellipsoids at 50% level. Hydrogen atoms are omitted for clarity. Selected bond distances [Å] and angles [°]: Sb1–Si1 2.502(4), Sb1–Si2 2.496(4), Sb2–Si1 2.502(4), Sb2–Si2 2.503(3); Si2–Sb1–Si1 71.05(11), Si1–Sb2–Si2 70.95(11), Sb2–Si1–Sb1 108.90(13), Sb1–Si2–Sb2 109.08(13).

V.3.5 Crystal structure of compound 5

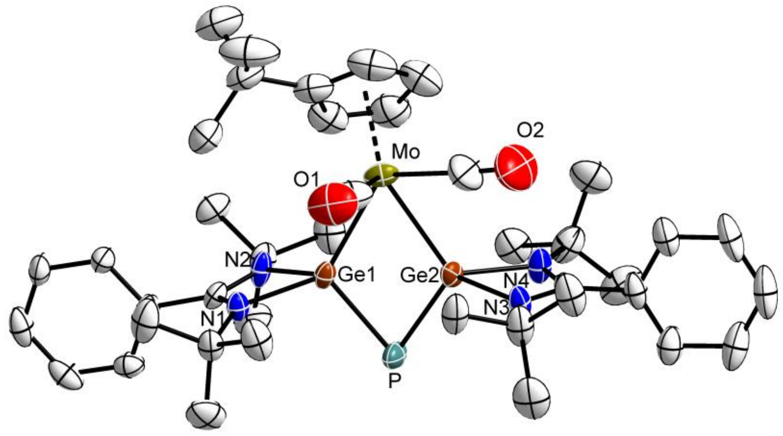

Figure S35. Molecular structure of the compound **5** in the solid state with thermal ellipsoids at 50% level. Hydrogen atoms and non-coordinating solvent molecules are omitted for clarity. Selected bond distances [Å] and angles [°]: Ge1–Mo1 2.6132(10), Ge1–P1 2.2453(13), Ge2–Mo1 2.5954(8); Ge2–P1 2.2368(14); P1–Ge1–Mo1 110.97(4), P1–Ge2–Mo1 111.90(4), Ge2–Mo1–Ge1 62.71(2), Ge2–P1–Ge1 74.41(4).

V.3.6 Crystal structure of compound 6

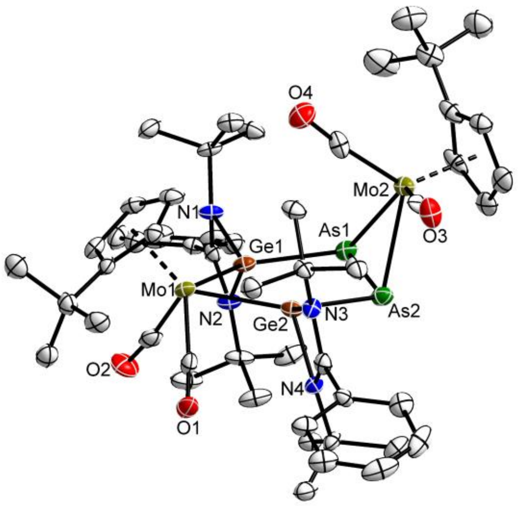

Figure S36. Molecular structure of the compound **6** in the solid state with thermal ellipsoids at 50% level. Hydrogen atoms are omitted for clarity. Selected bond distances [Å] and angles [°]: As1–As2 2.3478(9), As1–Ge1 2.4350(9), As1–Mo2 2.6542(8), As2–Ge2 2.4752(9), As2–Mo2 2.6457(8), Ge1–Mo1 2.6078(8), Ge1–N1 2.005(4), Ge1–N2 2.006(4), Ge2–Mo1 2.6306(8), Ge2–N3 2.014(4), Ge2–N4 2.014(4); As2–As1–Ge1 100.36(3), As2–As1–Mo2 63.52(3), Ge1–As1–Mo2 119.21(3), As1–As2–Ge2 99.01(3), As1–As2–Mo2 63.89(2), Ge2–As2–Mo2 109.10(3), As1–Ge1–Mo1 131.73(3), As2–Ge2–Mo1 131.40(3), Ge1–Mo1–Ge2 75.45(2), As2–Mo2–As1 52.59(2).

V.3.7 Crystal structure of compound 7

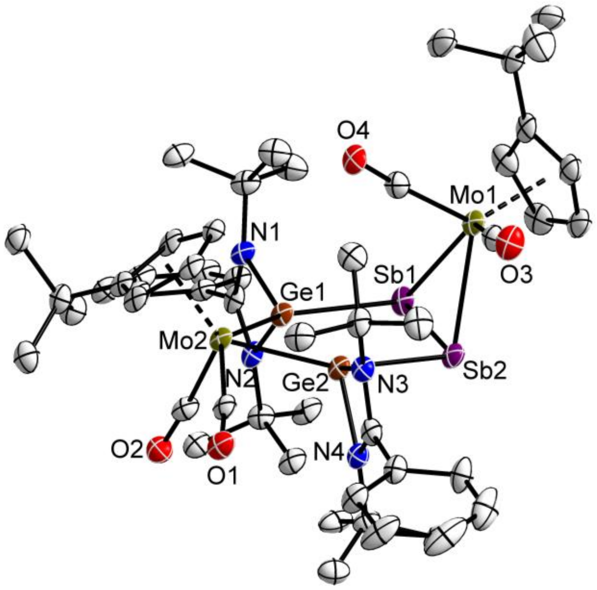

Figure S37. Molecular structure of the compound **7** in the solid state with thermal ellipsoids at 50% level. Hydrogen atoms and non-coordinating solvent molecules are omitted for clarity. Selected bond distances [Å] and angles [°]: Sb1–Sb2 2.7090(6), Sb1–Mo1 2.8265(6), Sb1–Ge1 2.6271(6), Sb2–Mo1 2.8495(6), Sb2–Ge2 2.6875(6), Mo2–Ge1 2.6242(6), Mo2–Ge2 2.6208(7); Sb2–Sb1–Mo1 61.922(15), Ge1–Sb1–Sb2 97.60(2), Ge1–Sb1–Mo1 114.73(2), Sb1–Sb2–Mo1 61.065(15), Ge2–Sb2–Sb1 93.85(2), Ge2–Sb2–Mo1 107.42(2), Sb1–Mo1–Sb2 57.013(15), Ge2–Mo2–Ge1 76.59(2), Mo2–Ge1–Sb1 134.13(3), Mo2–Ge2–Sb2 136.01(2).

V.3.8 Crystal structure of compound 8

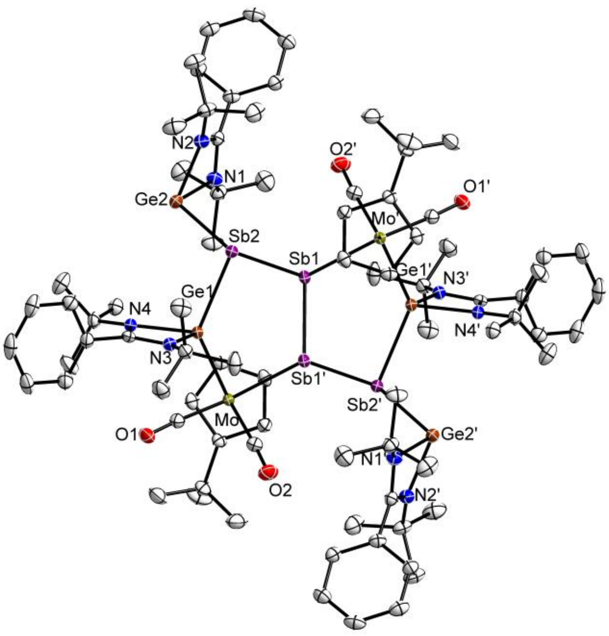

Figure S38. Molecular structure of the compound **8** in the solid state with thermal ellipsoids at 50% level. Hydrogen atoms and non-coordinating solvent molecules are omitted for clarity. Selected bond distances [Å] and angles [°]: Sb1–Sb2 2.8252(7), Sb1–Sb1' 2.8196(14), Sb1'–Mo 2.8773(10), Sb2–Ge2 2.7335(8), Sb2–Ge1 2.664(2), Mo–Ge1 2.5746(13), Ge1–N4 2.000(2), Ge1–N3 2.018(2), Ge2–N1 2.012(2), Ge2–N2 1.998(2); Sb1'–Sb1–Sb2 88.42(3), Sb1–Sb1'–Mo 98.65(4), Sb2–Sb1–Mo' 103.05(2), Ge1–Sb2–Sb1 88.31(3), Ge1–Sb2–Ge2 100.41(4), Ge2–Sb2–Sb1 106.62(2), Ge1–Mo–Sb1' 74.28(3), Mo–Ge1–Sb2 130.00(3).

VI. Quantum Chemical Calculations

Quantum chemical calculations of the resolution of identity standard density functional theory (RI-DFT) of the compounds **1** to **3** as well as **6** to **8** (no symmetry constraints), as well as the model compounds [Mo(CO)$_5$PMe$_3$] (C_s symmetry), **4**, **I-P** and **I-As** (D_2 symmetry) were performed by means of the program system TURBOMOLE[13] using the RI-BP86 functional[14-16] and the Grimme D3 dispersion correction.[17] The basis sets for each atom were of def2-SV(P) quality[18] as taken from the TURBOMOLE basis-set library. For Mo ans Sb effective core potentials (ecp) containing 28 electrons each were chosen. The vibrational spectra (frequencies, Raman intensities) were calculated using the modules aoforce[19] and egrad.[20] Imaginary frequencies were not found for any of the molecules.

The local force constant values were determined exploiting the vibrational Hessian by means of the routine LModeA-nano plugin[21,22] within the pymol program. We are aware of the difference of force constant values obtained from Wilson's FG method[23] and the local mode approach (see i.e. [24,25]) but with regard to diagonal force constants these differences are usually within the uncertainty of experimentally determined values.

Natural bond orbital (NBO) population analyses to determine the Wiberg Bond Indices (WBI) of the as-As and Sb-Sb bonds were performed using the program multiwfn.[26,27]

Cartesian coordinates, vibrational frequencies and raman intensities (given in a.u., cm^{-1} and percent, resp.) of the molecules under discussion:

1

5.82092925626815	0.99644535388932	-2.25241156871665	mo
1.79854043047616	-0.17821539716472	-1.00614739404703	p
6.77691619141364	3.45768116141305	-5.82726109175622	c
8.65652226572420	4.52164818115533	-2.04541947279270	c
6.15895231605707	5.56983239727735	-2.04337252145451	c
7.57494280952780	-0.72315912123008	0.54908549021800	c
6.31945639342477	-2.22031524147961	-3.91546769031013	c
4.98260422854636	4.89625841741575	-4.37478074270943	c
9.10017440183828	3.25928599654786	-4.41176702933844	c
-0.42143498757966	-4.42666705698516	-1.20123345949539	mo
-0.99351310009591	2.99955542393786	-0.28376840242777	si
6.46055222760212	2.72201248184975	-7.73984061927015	h
10.03736234149599	4.72854635769089	-0.50872140030322	h
5.27902194413984	6.63089415207564	-0.50046291919339	h
8.70567347530788	-1.66101394523718	2.20696621887397	o
6.70745930770302	-4.13034609536925	-5.01095874331820	o
3.04735951486627	5.37166154290316	-4.94232445738963	h
11.63068870292646	2.23595791506088	-5.35064570695986	c
-3.11398366625127	-1.97192056897339	1.86159647083035	si
-3.13156530016957	-3.72981240715861	-4.75856748903102	c
-3.38807936994284	-6.29713177014046	-3.92401208698263	c
2.93243393087103	-5.32705900543461	0.11434435764460	c
-0.59574105764380	-3.42166251589300	-5.65646858947562	c
-1.23316457674131	-6.61115663095492	1.64433333547383	c
-1.01131847758736	-7.59161438557864	-4.28491047048276	c
0.70583294404966	-5.76282493648539	-5.35017856666185	c
-4.46002008906799	2.01318490524279	1.59912352766828	p
0.42163854394809	6.11620116381329	0.88194921974148	n
-1.30478330713851	5.55527583693936	-2.79227155156154	n
11.27642061250719	0.64546198541987	-7.76861226643542	c
12.92834020010303	0.59339738506986	-3.31576499438627	c
13.32768600647173	4.54794806654959	-5.96176480532042	c

-6.00882393608443	-3.61152027607235	3.23405050897887	n
-2.62998039717068	-2.71751843651332	5.36687667145488	n
-4.62356492474282	-2.28872257130061	-4.77055288800385	h
-5.13362657221894	-7.16129370035597	-3.21873020350331	h
4.89010629214340	-6.01492309425064	0.87909365459456	o
0.24293527016457	-1.68211254844601	-6.40878556722426	h
-1.67331561726429	-8.06115595857270	3.28855247808444	o
-0.53426392043963	-10.43078124508159	-4.07045735787357	c
2.69294138019271	-6.08552930939026	-5.85125418847015	h
1.22533207276477	7.07480234607960	3.38581781555460	c
-0.35986147434314	7.31436330484363	-1.21611184019241	c
-2.69389490313039	5.73427825436841	-5.20438203874950	c
9.97461870984310	-0.96803474574701	-7.43452286054753	h
10.51271595615894	1.80004326256409	-9.35160484952599	h
13.12659533626238	-0.13195660238739	-8.39672264827295	h
14.84012037346624	0.00153572975490	-3.96577956967956	h
13.16562744039391	1.63337622138615	-1.50464580736656	h
11.79253543070571	-1.12664304986649	-2.91737841299843	h
12.41928728843224	5.78902210841792	-7.39657772688287	h
13.67940939169158	5.70400125137227	-4.24050266244450	h
15.18959876405004	3.92299466117586	-6.71937207135495	h
-8.38902658663070	-4.67512730298790	2.25157906219676	c
-4.91183470820476	-3.80239811041377	5.52521304398502	c
-0.68859709714737	-2.18318911511737	7.29635507483827	c
2.02102582397807	-10.98983763281827	-2.77665920123772	c
-0.48950065491734	-11.48741329287114	-6.80597273944079	c
-2.67046040945891	-11.73795790678934	-2.57738795934116	c
2.80101425034222	4.94675114357530	4.59816386293114	c
2.83338053454175	9.50364503529283	3.23156873165940	c
-1.16091486679111	7.59801459765698	4.99139829501281	c
-0.26039328312950	10.08117614151430	-1.70057922600920	c
-5.51648243945155	6.22574405191515	-4.62929774991420	c
-1.65649970037169	7.81196447619919	-6.96718017287208	c
-2.37528670057326	3.13571586039552	-6.48230007086966	c

-10.63091262321110	-4.14778316523901	4.04611439069198	c
-8.90352304337609	-3.34681273176792	-0.29120466370079	c
-8.09293390393065	-7.54955286149672	1.82538119246018	c
-6.03521074888042	-4.99808452124824	7.81041632765716	c
-1.34067922179361	0.36861087206599	8.56025171705894	c
-0.48964635280154	-4.28826068102838	9.30774686962800	c
1.87162936779831	-2.01960421724869	5.91330118491149	c
3.60544023700480	-9.95003095055193	-3.68285978984906	h
1.97243491819034	-10.43100253201079	-0.75469911309604	h
2.45346980547437	-13.04600346456919	-2.88338866508523	h
-0.23451518460627	-13.57650174419268	-6.78698155880615	h
-2.28571235945771	-11.05399742522895	-7.81077300113888	h
1.08848661058693	-10.64761465671736	-7.91244329020556	h
-2.84425776525029	-10.94450121336237	-0.64132348246821	h
-4.52836316820075	-11.54526688023134	-3.54320354903344	h
-2.26802679060256	-13.79383328763491	-2.39689624875330	h
4.49982153356966	4.49375771328198	3.44883224496115	h
3.40452436646198	5.49606372546818	6.53324549324667	h
1.66829351398029	3.19083671480689	4.75132899992126	h
4.47533351758370	9.28028621037611	1.94251208684286	h
1.72253904725073	11.16039684156195	2.58881750769872	h
3.57817564642393	9.93666160380489	5.14761704044771	h
-0.62220421537522	8.17205821431545	6.94098735278708	h
-2.29195563662825	9.15086113577184	4.13661016226589	h
-2.37242282063139	5.88432099688280	5.09038781859909	h
-2.33205052273227	11.60371222848061	-1.00266387290889	c
1.87624615769338	11.17833034248465	-2.85116524418074	c
-5.75670971564712	8.09061555368678	-3.68992717658254	h
-6.64139388260346	6.23642931931424	-6.40600407934141	h
-6.27236589731519	4.73892473356733	-3.35053141973669	h
0.41618611716071	7.61922771826897	-7.24993327885841	h
-2.58554178659660	7.64065721252223	-8.84420374746074	h
-2.04029003717292	9.73877798780164	-6.23678685275828	h
-3.06004162299644	1.61291162129212	-5.20824822942788	h

-3.47189703836494	3.04716386111339	-8.27104471160120	h
-0.35212644736982	2.76062147630198	-6.90774960906089	h
-12.43005812755996	-4.68266504255380	3.09937350752904	h
-10.49433588008819	-5.24690797261212	5.82588585589333	h
-10.71741011225054	-2.10481725944121	4.52992755332443	h
-10.62994527240791	-4.13990527497067	-1.18736721611072	h
-9.16184482516018	-1.28052741661963	-0.02096853664615	h
-7.29289057009630	-3.61795498249931	-1.60698033516603	h
-6.42506446092513	-7.94641254417587	0.61700697145237	h
-7.81368359428894	-8.55042343771865	3.64805832240333	h
-9.80769463304740	-8.34097904420278	0.90027540575286	h
-5.74076042715528	-7.61058005491061	8.23638799717662	c
-7.36493600065243	-3.50077741188136	9.56674763000867	c
0.17865963754245	0.95391574464633	9.89094904394946	h
-1.58687217439202	1.86015374983492	7.10415934549754	h
-3.13938366406498	0.21213512387815	9.63717602191995	h
-2.14124903332628	-4.33469975567800	10.59751140585083	h
-0.30633214727393	-6.16726657832176	8.38878174796266	h
1.22532697050423	-3.95226000613803	10.47539654778705	h
3.37053386265834	-1.37192968981553	7.23318077460159	h
2.42895212078825	-3.88640040507825	5.13829596633799	h
1.81052998684646	-0.68329326993236	4.29832026284632	h
-2.26170792438704	14.21831253910059	-1.45185933163694	c
-3.99273654982025	10.72862654742908	-0.10157801702647	h
3.48678905795486	9.97786802681542	-3.39625844558254	h
1.93460505781930	13.79157023926751	-3.29541699039414	c
-6.78308592537313	-8.71498778063773	10.41182332636546	c
-4.64698108807090	-8.73971862643070	6.87398560125759	h
-7.59658921147535	-1.46333838808025	9.20539023203286	h
-8.40237183252280	-4.61519463894539	11.73915410616496	c
-0.12941145214377	15.31469440900381	-2.59770440490701	c
-3.88259367367382	15.40582985287091	-0.90235607637808	h
3.60795451761362	14.64613349460561	-4.19477147920117	h
-6.54220726058239	-10.75689071549042	10.74810292483181	h

-8.11349204546784	-7.22326594580479	12.16381657342527	c
-9.44423000147003	-3.44094358051517	13.10901978644059	h
-0.07591084891165	17.36744493872969	-2.94935125621882	h
-8.92619276488493	-8.09658378806673	13.87219040618602	h

15.22	100.00
21.39	10.31
25.88	23.73
28.05	44.68
33.69	10.75
35.55	9.53
36.81	6.50
39.25	8.30
41.50	14.59
49.71	3.80
51.73	4.62
55.26	5.19
56.20	9.26
58.72	8.80
60.30	8.31
62.66	4.89
66.79	5.79
69.40	5.20
71.62	7.38
75.56	19.20
76.11	2.40
79.21	17.36
82.57	1.90
85.02	4.89
88.60	4.77
89.98	4.69
94.81	14.22
96.53	3.05
100.58	4.55
102.16	5.52
104.96	0.97
106.32	4.57
113.91	2.14
119.12	7.07
121.05	3.71
125.69	1.56
128.39	1.70
133.61	3.41
142.83	2.17
146.10	1.51
149.01	0.73

154.79	0.73
160.02	0.40
165.81	1.25
178.58	0.40
186.05	1.28
200.01	7.69
208.52	2.63
218.64	1.09
221.73	0.16
222.64	1.06
228.74	0.28
230.47	0.56
235.09	0.98
235.89	0.88
241.41	1.88
249.94	0.77
250.81	0.98
255.97	0.33
257.02	0.38
258.88	0.42
259.44	0.56
260.81	0.17
264.96	0.75
267.11	1.48
273.08	0.76
279.43	1.15
286.81	0.33
290.81	0.09
293.59	1.02
295.84	0.39
300.38	0.39
302.30	0.94
303.01	0.10
304.46	0.26
305.22	0.48
307.88	2.52
310.69	0.56
311.07	1.07
315.21	1.26
318.81	0.43
319.21	0.48
321.50	0.46
323.00	0.55
325.04	0.27
327.69	0.28
344.78	0.08
351.07	0.44

355.84	0.12
358.49	0.33
359.26	0.46
363.23	0.13
369.27	0.11
372.35	0.43
373.62	0.42
377.25	0.04
381.29	0.60
385.39	0.24
392.31	0.17
394.15	0.27
397.80	0.85
403.12	0.10
406.83	0.04
427.51	9.04
431.56	25.79
443.58	1.34
444.49	0.50
446.61	0.11
456.64	1.30
457.97	0.82
460.15	0.66
463.44	3.64
466.03	14.88
469.45	0.71
471.54	0.69
475.96	2.54
478.05	1.59
483.08	1.41
485.16	1.60
486.31	0.77
493.08	2.44
498.49	3.33
502.94	0.19
504.94	0.24
510.76	12.23
522.16	0.82
533.03	3.73
557.84	0.35
562.47	10.95
564.37	0.88
567.55	2.73
570.36	41.09
577.60	1.05
577.97	1.16
593.74	4.89

594.08	1.17
596.55	0.31
605.45	0.54
607.41	0.49
608.08	0.40
612.36	6.04
619.55	4.89
666.36	0.58
669.66	0.29
683.78	0.08
684.92	0.01
704.89	0.13
706.64	0.17
711.76	0.16
715.46	2.93
746.02	0.03
749.71	0.22
751.69	0.66
753.92	0.48
754.86	0.60
769.38	0.24
785.47	0.03
789.81	0.04
791.31	0.04
807.89	0.64
808.01	0.09
808.84	0.13
811.99	0.29
812.26	0.13
818.86	0.05
820.04	0.09
839.68	0.04
843.24	0.08
845.62	0.06
851.77	0.13
866.58	0.15
883.66	0.04
890.95	0.19
896.06	0.34
896.93	0.19
897.17	0.05
903.52	0.36
904.60	0.06
905.26	0.15
907.32	0.13
912.54	0.10
915.05	0.15

916.04	0.06
916.25	0.48
917.41	0.11
918.07	0.35
920.63	0.04
922.78	0.02
928.23	0.15
929.74	0.13
934.47	0.02
935.49	0.05
937.62	0.02
941.63	0.02
943.88	0.02
946.36	0.02
963.20	0.04
966.06	0.07
985.58	1.59
985.82	4.18
986.35	0.00
987.97	1.39
1013.23	0.06
1013.27	0.15
1014.42	0.53
1015.40	1.03
1017.34	0.27
1018.41	0.09
1018.71	0.04
1021.00	0.26
1021.41	0.07
1024.85	0.23
1027.12	0.30
1028.42	0.36
1029.13	0.17
1029.71	0.32
1031.94	0.06
1038.21	0.20
1038.89	0.63
1041.75	0.05
1046.94	0.24
1055.08	0.15
1070.39	0.06
1072.68	0.16
1075.90	0.02
1080.78	0.05
1146.60	1.58
1148.92	1.03
1150.64	0.13

1152.22	0.12
1165.78	0.24
1168.49	1.39
1170.99	0.53
1172.99	0.15
1181.99	0.20
1184.69	0.21
1194.76	0.46
1194.92	0.15
1195.43	0.81
1200.71	0.18
1208.16	0.10
1208.78	0.19
1214.65	0.16
1215.27	0.43
1227.50	0.39
1228.74	0.29
1228.97	0.07
1231.20	0.46
1231.65	0.05
1233.56	0.20
1266.69	0.92
1271.55	0.90
1271.94	0.33
1274.48	1.79
1297.93	0.04
1299.78	0.04
1346.14	0.05
1346.44	0.08
1347.04	0.05
1347.93	0.05
1348.25	0.12
1349.36	0.07
1349.54	0.05
1350.08	0.07
1350.78	0.14
1352.92	0.05
1355.40	0.04
1355.96	0.04
1356.53	0.11
1359.26	0.05
1362.12	0.01
1362.36	0.02
1378.49	0.14
1380.40	0.14
1381.33	0.41
1381.77	0.43

1383.02	0.12
1384.19	0.17
1393.09	0.05
1395.29	0.84
1400.48	0.10
1409.94	0.07
1410.82	0.13
1411.71	0.09
1412.33	0.19
1413.26	0.09
1419.23	0.06
1419.99	0.25
1420.71	0.02
1421.84	0.47
1423.24	0.25
1423.90	0.50
1424.02	0.43
1425.55	0.31
1426.49	0.45
1427.89	0.29
1428.28	0.28
1430.79	0.52
1431.36	0.34
1431.83	0.20
1434.70	0.39
1437.43	0.18
1437.80	0.04
1438.92	0.29
1440.57	0.60
1441.06	0.46
1442.44	0.57
1443.86	0.20
1444.72	0.27
1445.26	0.17
1445.43	0.13
1446.64	0.18
1448.12	0.64
1448.26	0.75
1457.26	0.02
1458.63	0.05
1460.83	0.17
1461.39	0.21
1462.30	0.34
1463.09	0.15
1469.04	0.39
1470.69	0.16
1472.65	0.72

1475.91	1.00
1476.15	0.77
1483.09	0.10
1528.98	8.99
1534.40	7.44
1591.12	0.12
1591.27	0.19
1618.14	9.44
1619.00	7.03
1877.92	0.29
1880.66	0.09
1942.08	3.83
1981.39	1.23
2927.32	0.58
2929.75	0.45
2936.19	0.46
2937.18	0.56
2937.67	0.12
2940.24	0.21
2941.46	0.36
2941.68	2.51
2945.18	1.68
2947.14	0.12
2947.28	0.21
2951.45	0.26
2951.86	0.37
2952.68	1.11
2955.36	0.15
2957.42	2.00
2959.54	0.60
2961.40	1.41
3012.95	0.34
3014.96	0.15
3016.08	0.12
3018.41	0.65
3020.28	1.30
3020.83	0.39
3024.45	0.19
3025.97	0.55
3026.99	0.11
3027.62	0.28
3029.54	0.16
3030.59	0.24
3033.27	0.14
3034.95	0.12
3035.26	0.09
3035.46	0.19

3037.43	0.14
3039.00	0.09
3039.92	0.30
3042.43	0.58
3042.73	0.02
3044.50	0.21
3044.79	0.27
3044.84	0.12
3044.88	0.19
3045.31	0.22
3048.58	0.36
3050.41	0.08
3056.65	0.13
3057.00	0.13
3057.50	0.11
3059.38	0.12
3060.66	0.12
3061.74	0.05
3063.82	0.31
3068.76	0.18
3090.06	0.14
3092.88	0.11
3099.91	0.56
3101.35	0.51
3108.84	0.08
3108.85	0.61
3115.64	0.06
3116.86	1.17
3121.11	1.85
3127.76	0.45
3149.52	0.26
3156.26	0.06
3161.31	0.15
3164.03	0.07
3174.14	0.13
3174.31	0.14
3180.29	0.16
3194.15	0.31

2

5.15428587913817	5.68151328509770	8.22225358376784	mo
9.35367349751854	8.60183556271216	9.61456558865994	as
5.23973012533593	9.67238291443601	10.41424968694115	si
6.22735881736213	3.54014549390150	11.02893562585982	c
5.27700684446581	6.43163614360824	3.76942733986616	c

7.31707798593161	4.73054764485468	4.33138233542592	c
6.27018864107175	2.44300024952588	5.29783117077765	c
2.97136496816441	5.16639273460392	4.40076350545356	c
1.99138140008266	6.38472421543439	9.98781410306528	c
3.55160151948920	2.66925003946065	5.36585445991053	c
9.88128736852609	11.16345512126909	5.60729135890425	as
3.93138264491593	10.48297875321072	13.58244688142104	n
3.13811026888940	12.49795744123934	10.09924576114767	n
2.50508231901918	12.33338837146100	12.57689037393047	c
6.85355455492486	2.09194360347872	12.59890785655330	o
5.47191260758654	8.32848726659130	2.95546087745678	h
9.33772299176712	5.13461198996862	4.08387857190894	h
7.36243311413773	0.77964612561044	5.88803739782982	h
1.06401298258574	5.93869375144285	4.13982238529657	h
-0.00934552639599	6.81542748285597	10.92217163267857	o
1.68136332171659	0.52749153982232	5.86716392130241	c
14.70576384354165	13.14996249580637	5.50555756429979	mo
10.83434403956430	15.16773844000134	6.82813345901450	si
4.39661827298217	9.75911076255694	16.24463164579939	c
1.96735767404928	13.94969059467776	8.01270559184350	c
0.46496777650505	13.75506102643702	13.88214994111232	c
1.80761595933473	-1.28461586682230	3.56529106732732	c
2.36935756902149	-0.93799112378513	8.29438626616846	c
-1.03475192883809	1.55697602094946	6.12262008768566	c
15.51680002271338	11.59619402308560	8.75456416849042	c
15.11704498224944	13.50032260685405	0.97846936576245	c
14.83275756043487	10.87041660895333	1.53759308415110	c
15.32188558689993	16.44186350256577	7.05923542105193	c
16.91627689749756	10.03511476297362	3.06873543205319	c
17.38783501388374	14.34180139058027	2.19444620958577	c
18.50906742901675	12.21933494508797	3.46947255584008	c
10.15199767684608	17.26791453391807	9.56278677748214	n
9.44594101107722	18.17783124183767	5.63695197102187	n
9.47345619285934	19.16072048947657	7.98823453343225	c

5.87978096216527	11.90542922819782	17.54872436626553	c
6.00060463081167	7.33248100500507	16.19985912304609	c
1.90872400811934	9.20960367754522	17.66721677132626	c
2.83300865521309	12.72432407567545	5.51982024146227	c
-0.94841801444824	13.87964138063001	8.12500234866488	c
2.94365079927609	16.69570455765320	8.13686815733999	c
-1.90357854778708	12.55946688943822	14.17373719740966	c
0.83045657171890	16.22789208162522	14.79368937348219	c
3.72508111818440	-2.12534468174176	3.37050918465315	h
0.42419731123537	-2.85673695793347	3.78031701185370	h
1.36908048425372	-0.26354164966822	1.77961296743611	h
2.16933064862903	0.29347873995786	9.98221009304262	h
1.10682645603485	-2.60652787880424	8.52068271175486	h
4.35070468296726	-1.63608506212093	8.25411587637960	h
-1.69437282759370	2.46209403228089	4.34288105111878	h
-2.36333121809558	-0.01492810958322	6.55500179807773	h
-1.16978218685135	2.96834486056719	7.67213942976671	h
16.16135596730699	10.63154064180389	10.65328156712408	o
13.85956464720922	14.64031514645185	-0.21427545339825	h
13.27724398434005	9.67461475444356	0.86178161032921	h
15.74473700514517	18.50095449994086	7.85333903209876	o
17.49904963249488	7.29199824884953	3.75870537434868	c
18.17661375949315	16.26098004131289	2.11427385435011	h
20.30212249990411	12.24612574021508	4.50837145691660	h
10.32942347273090	17.18271982791872	12.34923933841384	c
9.20546700783767	19.32705898148252	3.10928756355375	c
9.03574276028462	21.84728040851397	8.67065613325905	c
4.76210798530847	13.68537857843250	17.56374233541718	h
6.31620617606535	11.38916685940436	19.53916631760118	h
7.68659352278363	12.25897992991906	16.53753067367995	h
7.76851215578861	7.59722316579296	15.09411041193264	h
6.53479961354888	6.79852403281567	18.15919038657436	h
4.93400611392471	5.74140234396457	15.35164938352229	h
0.71897196372745	7.87841625315978	16.56196158082753	h

2.35133923893083	8.32449608989515	19.52109043880019	h
0.80229916603437	10.95096285747356	18.03885822057762	h
2.11580350234187	13.84278391939193	3.89213615794968	h
4.93264303225913	12.65704866197938	5.39491882391204	h
2.10534863971189	10.76135098371273	5.36579662768716	h
-1.73201927509729	14.64588689684580	6.33177898555647	h
-1.61942456621933	11.90187465485242	8.35410227796600	h
-1.71716126686499	15.03007274090339	9.69937184953244	h
2.35682889939414	17.61095629820932	9.93457160332450	h
5.03816716543359	16.71813855286604	8.02024356258524	h
2.17550410927518	17.82655729350036	6.53917172111038	h
-2.14910043444188	10.62621493252732	13.44141438433024	h
-3.89184915283081	13.84678359645314	15.35999145832817	c
2.68339473078028	17.14602558580581	14.57866740995522	h
-1.16732624225717	17.50272947526624	15.99342443076397	c
18.35797700862175	5.94680416828809	1.29862837361518	c
19.64607423842473	7.13969688666594	5.72445623061733	c
15.13304060513878	5.94561730640203	4.81464055288096	c
11.69619055288562	14.71084622699659	13.03762079377303	c
7.62708921867239	17.16275214499611	13.44028748036653	c
11.85759756766564	19.43692721900985	13.39809894070662	c
8.37738526592168	17.16804662782384	1.33067322220319	c
7.18803096355600	21.42999387919942	3.06408883245081	c
11.80354723253000	20.37480757894015	2.28417016340884	c
6.61666087160558	22.73999103679364	9.32947615795685	c
11.11249670579705	23.51915780772211	8.60893874450350	c
-5.74108155785901	12.91274632199218	15.57699671462139	h
-3.52804981003456	16.31878542531985	16.27230844861342	c
-0.87246668229227	19.43414607097388	16.71618570116670	h
20.03348173703915	6.90533333883811	0.46394358793932	h
18.87437382116338	3.94435507443581	1.69130232752872	h
16.82721732534852	5.95466611701748	-0.14343043296411	h
19.14854984956523	8.16978920457816	7.48384307491651	h
20.01555439239817	5.13287060785961	6.23186451418451	h

21.43972545630746	7.93756623920222	4.97163365282060	h
13.52422285058137	6.05354508844907	3.46608733567831	h
15.54066371266529	3.91106652384950	5.16117035396900	h
14.50730888811525	6.81516650692754	6.62005121038840	h
13.66936445377851	14.72082911617875	12.33048129953820	h
11.74846610526241	14.48350257034620	15.12456094925440	h
10.71538554657013	13.03459157330810	12.22734691498751	h
6.56229997112951	15.51533411135694	12.69066632459931	h
7.67832728540623	17.03055868671256	15.53552191471361	h
6.60290954691557	18.92111700766171	12.91631108304147	h
10.81826945527310	21.25040294005877	13.22908607770502	h
12.25791059475167	19.11467672091397	15.43562153404781	h
13.68287295799012	19.60883703431177	12.37485184053872	h
8.22576632793620	17.86823841123104	-0.64397070202416	h
9.76833857958756	15.59271135290651	1.37271966074789	h
6.51594939999792	16.39308950513578	1.92237659098522	h
5.38354310197639	20.74389815183335	3.89520786011557	h
7.79438933295668	23.13693638224997	4.11923057379438	h
6.82600073213149	21.99891253215290	1.07401550190263	h
11.70500874730183	21.13751805448857	0.32730073767556	h
12.41701580829563	21.92036247386156	3.56657229977150	h
13.25359608280564	18.85622807698240	2.35446209870203	h
5.00203726099016	21.42804497242012	9.34015361141089	h
6.27485570533234	25.30405825419945	9.92250518664701	c
10.75594262330272	26.07954301834964	9.19994070354274	c
12.99733733225866	22.77010658470849	8.13691726233235	h
-5.09361164745427	17.32484805162574	17.20925680692549	h
4.37975984171694	26.00303165586741	10.43223730614403	h
8.33980625646703	26.97517608841346	9.85531469720328	c
12.37964592395120	27.38374628529733	9.15763393538998	h
8.06551285054223	28.98773747972140	10.31909424702338	h

11.05	93.71
15.19	100.00
20.35	13.27
23.13	5.20

26.10	58.40
32.13	55.56
32.63	17.14
33.71	9.08
38.26	33.22
38.90	25.11
40.15	41.46
46.50	47.02
46.82	35.10
51.34	24.78
53.32	41.15
53.95	6.45
60.96	23.98
62.49	21.30
64.07	18.42
65.07	6.57
69.32	43.81
76.23	7.71
77.76	16.66
80.97	31.49
84.33	6.08
86.67	4.88
88.68	6.88
90.69	20.80
92.98	13.77
97.27	9.30
99.40	8.30
104.31	10.13
109.16	4.73
111.37	9.92
114.03	1.65
121.93	10.52
123.05	6.29
125.82	3.60
130.55	3.12
134.97	8.11
139.50	0.79
149.80	3.41
152.44	11.60
160.16	11.47
160.67	3.54
166.97	1.61
173.38	1.96
194.59	0.92
199.38	4.52
215.94	0.74
218.94	1.71

220.58	10.35
223.42	1.00
224.48	26.65
230.39	4.50
233.80	9.90
237.30	3.64
238.30	1.80
248.98	0.11
250.37	1.48
251.07	2.48
256.61	0.90
258.66	0.29
260.30	0.61
268.50	0.68
270.64	1.07
280.96	1.83
282.02	2.04
285.76	1.87
289.46	0.48
295.48	2.90
296.50	0.26
300.43	0.56
300.93	0.19
303.54	1.19
309.00	5.18
310.58	0.59
312.95	3.71
314.25	0.52
315.82	1.11
319.67	2.11
321.01	2.82
326.62	0.18
328.17	0.06
333.22	0.40
334.48	0.94
341.64	0.36
345.70	0.33
350.84	0.66
351.75	3.57
356.75	1.01
358.72	1.95
364.52	0.30
367.77	1.97
372.22	0.84
375.14	0.60
380.70	0.75
383.42	0.29

389.04	0.19
389.88	0.34
398.02	0.76
398.11	1.06
399.79	0.12
403.83	7.19
406.68	0.18
407.90	0.80
451.48	0.11
451.92	0.39
452.15	0.23
454.88	0.31
461.76	0.23
464.88	0.83
465.72	0.39
470.13	0.87
477.04	0.47
478.06	0.72
481.52	6.48
485.34	1.65
485.82	7.36
488.63	0.06
497.48	6.02
501.63	7.30
503.00	1.76
504.54	4.86
509.50	0.56
510.09	0.41
533.46	0.48
538.94	0.45
559.58	1.90
565.18	0.17
565.51	1.34
566.05	1.24
576.75	0.89
577.87	1.67
591.31	1.50
594.47	0.06
606.86	0.72
607.35	0.61
613.08	0.99
613.82	0.71
637.19	8.80
642.17	13.08
665.41	0.69
667.39	0.34
683.71	0.02

685.01	0.01
700.94	0.39
702.49	1.70
707.09	0.34
707.46	0.10
758.20	0.80
758.56	2.45
763.00	7.47
766.04	11.00
768.10	4.82
777.00	1.25
787.25	0.44
794.81	0.15
795.06	0.31
800.22	0.07
808.90	1.05
809.86	1.29
814.04	0.62
814.54	0.29
819.61	0.25
820.52	0.11
846.46	0.12
847.24	0.39
848.74	0.10
850.13	0.12
881.06	0.34
881.25	0.43
892.67	0.64
894.97	0.44
897.57	0.35
898.48	0.20
906.81	0.23
910.79	0.30
911.94	0.33
914.62	0.35
915.95	0.95
916.48	0.46
916.57	0.64
920.19	0.22
922.17	0.33
923.80	0.36
925.11	0.04
926.27	0.05
928.43	0.29
928.70	0.36
940.22	0.05
941.51	0.06

943.23	0.03
946.56	0.05
952.49	0.04
953.38	0.14
967.76	0.23
969.53	0.07
985.88	4.54
986.28	5.44
989.19	0.83
991.06	0.03
1010.33	0.07
1013.39	0.08
1017.28	0.89
1017.94	0.07
1018.64	0.10
1019.35	0.25
1022.04	0.35
1022.70	0.19
1026.37	0.65
1027.10	0.32
1028.46	0.55
1029.28	0.06
1029.98	0.73
1030.83	0.39
1033.68	0.52
1034.91	0.31
1036.54	0.31
1037.08	0.26
1042.41	0.32
1044.52	0.65
1076.09	1.39
1077.32	0.08
1078.01	0.87
1079.76	1.11
1145.16	3.25
1147.26	3.09
1151.33	0.31
1151.64	0.23
1167.16	0.45
1167.72	3.09
1171.28	0.93
1172.32	1.63
1179.62	0.52
1180.92	0.26
1194.52	0.82
1194.62	1.80
1197.15	0.08

1200.38	0.05
1213.29	0.67
1214.60	0.18
1219.44	0.48
1220.13	0.25
1227.52	0.16
1227.99	0.21
1230.16	0.54
1233.81	0.21
1235.01	0.44
1237.70	0.14
1267.73	1.52
1272.27	1.27
1274.20	2.36
1283.59	2.60
1298.44	0.02
1298.67	0.06
1338.32	0.03
1344.74	0.18
1348.17	0.16
1348.54	0.12
1348.61	0.07
1348.90	0.12
1349.60	0.06
1351.07	0.10
1351.19	0.06
1353.22	0.13
1355.68	0.04
1356.16	0.09
1356.36	0.09
1356.55	0.06
1361.34	0.02
1361.50	0.03
1379.48	0.46
1379.79	0.33
1381.05	0.19
1381.34	0.17
1383.36	0.20
1384.17	0.21
1388.44	0.07
1393.13	0.15
1399.64	0.15
1412.26	0.17
1412.83	0.04
1413.02	0.32
1414.56	0.16
1416.08	0.14

1417.97	0.07
1418.57	0.16
1419.13	0.05
1421.49	1.34
1423.73	0.72
1423.93	0.77
1424.63	0.69
1428.53	0.61
1429.08	0.78
1430.17	1.25
1430.51	0.78
1430.70	0.76
1433.00	0.37
1433.49	0.34
1436.26	1.74
1437.02	0.52
1437.72	1.79
1440.25	1.22
1441.77	1.73
1441.86	2.06
1441.89	0.22
1442.75	0.19
1444.41	0.78
1445.26	0.44
1447.13	1.36
1447.19	1.60
1447.45	0.46
1448.93	1.14
1450.30	0.64
1455.00	1.22
1455.19	0.31
1456.49	0.17
1462.11	1.25
1464.14	1.32
1465.10	2.70
1467.17	3.08
1470.58	5.37
1470.97	1.00
1473.06	1.56
1474.77	1.60
1518.28	31.14
1522.47	33.89
1590.36	0.15
1590.47	0.39
1617.11	11.03
1617.52	21.19
1845.20	1.20

1853.98	1.20
1924.19	0.83
1931.63	4.40
2927.91	0.92
2928.35	0.98
2929.44	1.35
2937.46	0.18
2938.82	0.57
2939.56	1.10
2941.68	4.80
2943.05	0.11
2943.15	0.36
2944.05	2.93
2945.55	1.06
2947.04	0.06
2949.30	0.24
2950.78	0.97
2950.84	0.20
2951.09	3.60
2953.94	1.09
2954.34	5.19
3013.05	0.55
3014.08	0.45
3016.27	0.80
3017.07	0.57
3017.27	0.05
3020.92	1.24
3021.37	2.04
3022.25	0.74
3022.67	0.53
3025.60	0.46
3030.82	0.40
3031.48	0.16
3032.59	0.26
3033.10	0.35
3033.66	0.22
3034.85	0.48
3035.97	0.38
3037.58	0.04
3039.01	1.21
3040.99	0.24
3041.02	0.22
3041.08	1.78
3041.72	0.35
3044.64	0.34
3044.81	0.35
3046.51	0.22

3046.55	0.25
3051.74	0.18
3052.86	0.35
3055.59	0.30
3056.99	0.19
3057.33	0.17
3057.70	0.22
3062.93	0.35
3063.11	0.21
3069.64	0.37
3092.62	0.32
3092.69	0.27
3102.18	1.10
3103.07	1.12
3110.33	0.79
3112.25	1.25
3117.94	1.07
3120.03	1.75
3122.88	2.47
3129.33	0.98
3145.00	0.43
3147.63	0.36
3153.60	0.25
3156.13	0.24
3161.84	0.32
3166.47	0.40
3172.63	0.57
3174.53	1.29

3

-2.69549141984545	-7.00444755615354	0.01935864875944	mo
2.04504541755271	-4.20862559238392	1.41706735350017	sb
-2.46649431698932	-2.91518039243137	2.04683529799753	si
-1.55176166860585	-9.09871115297487	2.83931017883862	c
-2.73279581509822	-6.36524193851314	-4.45628971069977	c
-0.74596165194955	-8.14462666031531	-3.95109245516611	c
-1.84752920706430	-10.35477256011976	-2.86858845774787	c
-5.06151723868335	-7.50714026537773	-3.68694104174844	c
-5.75752248881329	-6.26085817352914	1.93269492692260	c
-4.54648022598180	-10.00336111138765	-2.68197419080397	c
2.60634182953551	-1.37186765578293	-3.25804914211346	sb
-3.80487651660111	-1.95544413867523	5.17952238220272	n

-4.49040989095549	-0.03882677494636	1.62117801816587	n
-5.12429092348912	-0.06935248872352	4.10276891652701	c
-0.90906171835970	-10.50812780834391	4.43671773810956	o
-2.50117450478480	-4.50472099535653	-5.34232941398118	h
1.27362475991121	-7.85493477289103	-4.33366165825078	h
-0.80490408238588	-12.05350741849995	-2.28911565696801	h
-6.94261929572614	-6.65634377777692	-3.88525058602855	h
-7.70221120812359	-5.83213176249582	2.97299775132946	o
-6.47939394500056	-12.05262221344526	-2.04604400606704	c
7.64052257269197	1.10303624529109	-3.14336948969418	mo
3.67144886669274	2.98137074805889	-1.84350419087441	si
-3.44894298501049	-2.65014259730118	7.86510507734776	c
-5.46125238861888	1.49314035379545	-0.50561375135939	c
-7.05897359583025	1.54234801451984	5.34586623725204	c
-6.49427270687309	-13.94778476888480	-4.28358499696716	c
-5.76792887630886	-13.45742660978932	0.41045957647709	c
-9.14653320666956	-10.91287521507081	-1.74780564823013	c
8.31535873794223	-0.69509963877866	0.00757854592749	c
8.11249054758238	1.67560926170763	-7.63522993370831	c
8.09535792692384	-0.99655069099824	-7.19557014791813	c
8.17434991176365	4.26485341956119	-1.31798091248088	c
10.22045436591862	-1.67342038423102	-5.64566830758046	c
10.25430224132444	2.69470291497826	-6.33061937972330	c
11.55905533747345	0.64411451657779	-5.10477275858006	c
2.94453174406451	4.95811823111166	0.98848574850057	n
2.27663229264003	6.04837710822178	-2.89908408507781	n
2.24133394825975	6.90785200817063	-0.49832388677604	c
-1.77790200037608	-0.62238788707711	9.13401147285156	c
-2.09631397437121	-5.22588082832821	7.91460419083039	c
-5.99453073157117	-2.91550712036252	9.26740689306716	c
-4.83543896543617	0.05090590901472	-2.95338999082599	c
-8.34981120628649	1.88803329045444	-0.36994938070400	c
-4.08000188521113	4.06327770874388	-0.49915332979941	c
-9.52753423297528	0.56771215764148	5.62166016483316	c

-6.49115106988388	3.99514420221330	6.20551163553874	c
-4.61824760433239	-14.87109671831820	-4.50539056664832	h
-7.93011196424150	-15.45493442745505	-3.97023146127698	h
-6.94891695286526	-12.97344382336035	-6.09122535380803	h
-5.86425299971923	-12.16034693956866	2.05784182826008	h
-7.08456836253896	-15.06630240307194	0.73756234076921	h
-3.81662748015132	-14.23288761489771	0.33198607496998	h
-9.82301283709207	-10.04013305004531	-3.53742094438643	h
-10.51864402580989	-12.41965730283689	-1.22842068379510	h
-9.18660154087407	-9.45128832132685	-0.24079985883631	h
8.84925299917388	-1.81395448034795	1.85697202838164	o
6.77302031521695	2.72682037649191	-8.82024604629767	h
6.71011707642987	-2.32239115698591	-7.98800467698840	h
8.56778385946864	6.25520074929569	-0.35887332014221	o
11.08741270523232	-4.36535207351154	-5.05954178743344	c
10.84650748176132	4.68497060579573	-6.31856542373489	h
13.31697008190991	0.81200792650135	-4.02035633128239	h
3.07119099338504	4.73172325303031	3.77049742718412	c
2.08177459068687	7.33797891764132	-5.36190451368127	c
1.69941128561370	9.53837677818136	0.31547408013680	c
-2.72538078531084	1.25363734241209	9.09255720752451	h
-1.40722110552166	-1.12574304423737	11.14129204704222	h
0.06208838700545	-0.46300203645195	8.13130720119688	h
-0.27093050997780	-5.15825190310189	6.87590670139391	h
-1.68758036389710	-5.77576871541526	9.89940233988267	h
-3.28559198010783	-6.71611520708987	7.04414269059188	h
-7.29225422609968	-4.15950665434509	8.18251197001831	h
-5.65880623494983	-3.78423127121444	11.15117562011356	h
-6.93203190513416	-1.06654219647753	9.57720072799334	h
-5.43696770614666	1.17734247866014	-4.62166173192685	h
-2.76505912547766	-0.28582118537701	-3.11577006007275	h
-5.81294352845497	-1.80649978031615	-2.99460140662066	h
-9.01482023001963	2.72952097718464	-2.17729514880100	h
-9.33374241105717	0.05264966023487	-0.09367656863643	h

-8.91190705121483	3.18212421367879	1.17977712527564	h
-4.50781861379046	5.11085030103636	1.27111377571014	h
-2.00476952910571	3.77242180889746	-0.62393614719534	h
-4.68657020655180	5.24097692191463	-2.13224789383318	h
-9.93135042392083	-1.35819403951888	4.94184381457505	h
-11.41421151503799	2.05431687986118	6.73866286390035	c
-4.55999868503630	4.73707972236647	6.00003299429275	h
-8.38822895419984	5.47248659287753	7.33417768477638	c
12.17411596114501	-5.47688511287343	-7.54616271075546	c
13.17407877889417	-4.37003889455088	-3.02339271947379	c
8.86359816581920	-6.02726162198288	-4.15355189374760	c
4.61433904199537	2.34156964016078	4.36432288927003	c
0.35922604465172	4.44575514159769	4.79953074562010	c
4.39592678116884	7.03204452578243	4.98379709158124	c
1.32769772907520	5.28061739491735	-7.28692303907738	c
0.04059322487023	9.41903436805320	-5.34374718980919	c
4.68725525948309	8.45937502588642	-6.05913955153270	c
-0.76277574973779	10.30372060000319	0.97448173012339	c
3.71144735229353	11.28644698589914	0.39118129023122	c
-13.34168380667916	1.29199685030764	6.94621607465411	h
-10.84909193182457	4.50845729784818	7.59548347322811	c
-7.93642770069223	7.38877937998592	8.01511978354562	h
13.75179786774972	-4.29135778118740	-8.27290262528371	h
12.90800292363254	-7.42386268759996	-7.22782700304376	h
10.69876981907731	-5.57210980223150	-9.04177864800798	h
12.50666244769389	-3.48088979711302	-1.24306749064123	h
13.74853068323198	-6.34489028591449	-2.58608713202985	h
14.89141184570768	-3.34674455249641	-3.67423497338804	h
7.28199575282965	-6.03896991613582	-5.53866845609926	h
9.50065230983152	-8.01565088349890	-3.89519711243876	h
8.10218167635643	-5.33229882965027	-2.32523767046781	h
6.59666251748948	2.54144791452274	3.71233028534415	h
4.62640210056665	1.99555497598001	6.43558416242130	h
3.77991575531232	0.64662446815592	3.44036885208928	h

-0.56505658984681	2.74859268022214	3.97396648359305	h
0.38308384831353	4.24022833480556	6.88940028290746	h
-0.78456378559143	6.14072255291506	4.31514092992917	h
3.22206311998295	8.76747038451190	4.89992430146681	h
4.77992118814822	6.61616291067825	7.00745483958422	h
6.22337020540644	7.40410920350921	4.01971631450945	h
1.22196724485282	6.09384780501297	-9.22084480107631	h
2.73835607492450	3.72219860392325	-7.29997247508093	h
-0.53958016016962	4.45215569471198	-6.79493817983496	h
-1.77642570510116	8.66919940487080	-4.59948537202610	h
0.59771653434300	11.07117991264101	-4.18011231372072	h
-0.27708519010601	10.09410777213700	-7.30817593617333	h
4.63136743104558	9.33273222662947	-7.97101492178698	h
5.24931044865133	9.93519796418253	-4.67492634390504	h
6.15243078802233	6.95357661933126	-6.03726258924049	h
-2.32457544512579	8.93298647592541	0.87717652336117	h
-1.21241784843647	12.81518270033722	1.70461749366753	c
3.24872337766325	13.79412442903562	1.11834796934037	c
5.63270609343980	10.63739968260455	-0.08145912637903	h
-12.33564550762318	5.67219132485820	8.47692757065780	h
-3.14155415674403	13.41408283975800	2.21432695085845	h
0.78900401752085	14.56187454837799	1.77424583347161	c
4.82250412714932	15.15731384193607	1.18336330504912	h
0.43096756569598	16.53320138331261	2.34563223481972	h

4.57	100.00
17.83	10.20
20.07	4.68
23.45	1.47
25.86	3.77
28.33	2.45
31.27	0.73
32.41	6.88
35.53	3.29
36.77	2.65
38.41	1.72
42.78	1.16
43.64	5.57
47.39	0.83

51.53	3.47
55.25	3.23
56.66	1.06
57.45	2.15
61.71	3.80
62.31	0.85
65.15	0.38
72.50	0.45
74.23	0.88
76.81	2.23
81.92	0.66
85.65	1.17
86.24	0.39
87.34	1.35
91.08	1.21
95.15	0.71
95.98	0.58
99.15	0.56
101.22	0.81
102.74	0.77
107.48	0.66
111.72	0.58
116.99	1.41
121.36	0.06
125.76	0.54
130.91	0.28
132.95	0.14
140.01	0.31
142.01	0.48
153.88	0.05
154.40	0.56
161.05	3.17
162.78	0.12
172.50	0.52
179.50	0.36
182.18	0.36
211.72	0.15
213.09	0.27
219.14	0.01
223.53	0.01
228.76	0.13
231.86	0.30
235.96	0.14
237.70	0.13
249.01	0.12
251.45	0.17
255.48	0.01

257.63	0.07
258.31	0.01
260.12	0.07
265.74	0.02
269.31	0.08
276.24	0.09
280.30	0.03
283.12	0.33
285.11	0.03
295.56	0.27
298.78	0.01
300.36	0.03
300.95	0.01
304.01	0.13
307.01	0.26
309.17	0.23
310.03	0.05
314.21	0.34
315.63	0.12
318.92	0.12
321.64	0.01
323.07	0.17
329.61	0.05
331.97	0.03
334.31	0.02
340.09	0.05
342.11	0.04
348.94	0.21
351.16	0.13
356.89	0.10
358.76	0.07
362.53	0.05
362.84	0.28
369.76	0.05
373.05	0.05
380.92	0.08
383.34	0.01
384.00	0.02
386.43	0.02
389.65	0.18
395.08	0.22
396.88	0.03
397.96	0.02
406.09	0.02
406.90	0.04
451.31	0.02
451.75	0.01

451.94	0.02
455.05	0.03
461.58	0.07
463.31	0.03
463.95	0.03
466.83	0.56
469.30	0.24
470.50	0.83
474.24	0.02
477.07	0.02
485.20	0.03
487.03	0.00
498.17	0.32
499.51	0.36
503.77	0.34
504.35	0.56
508.09	0.05
509.21	0.03
532.57	0.05
536.16	0.05
558.93	0.10
564.86	0.02
565.54	0.05
566.03	0.11
577.54	0.08
577.83	0.13
590.85	0.08
593.73	0.01
606.77	0.05
607.18	0.05
611.78	0.04
615.02	0.05
628.70	0.69
632.90	0.98
664.47	0.03
667.73	0.03
683.66	0.00
684.67	0.00
699.71	0.02
700.76	0.07
706.56	0.03
707.12	0.01
757.11	0.17
757.63	0.34
760.58	0.43
762.85	0.99
768.93	0.14

776.87	0.09
787.08	0.04
793.62	0.01
794.25	0.01
798.30	0.01
809.01	0.09
809.52	0.10
813.79	0.05
814.21	0.03
818.08	0.01
819.42	0.01
845.01	0.01
845.96	0.01
846.87	0.03
848.49	0.01
879.99	0.04
880.32	0.02
892.08	0.09
895.61	0.04
896.50	0.03
897.66	0.02
906.81	0.02
909.42	0.01
910.49	0.03
913.79	0.02
916.54	0.02
916.96	0.08
917.19	0.08
918.69	0.01
919.85	0.03
923.14	0.02
924.60	0.00
926.08	0.01
928.76	0.02
928.77	0.03
939.99	0.00
941.80	0.00
943.92	0.00
946.86	0.00
949.65	0.00
951.65	0.01
966.91	0.02
968.21	0.01
985.82	0.28
986.40	0.41
988.20	0.11
989.10	0.03

1009.99	0.00
1013.52	0.01
1016.69	0.06
1017.29	0.01
1018.88	0.02
1019.38	0.01
1021.54	0.03
1022.31	0.01
1026.09	0.05
1026.20	0.01
1028.29	0.02
1028.96	0.01
1029.03	0.09
1030.67	0.03
1033.26	0.04
1034.28	0.02
1035.19	0.04
1035.62	0.01
1042.03	0.03
1044.50	0.05
1075.16	0.15
1076.08	0.07
1076.37	0.00
1077.41	0.08
1144.51	0.34
1146.85	0.26
1151.18	0.03
1151.44	0.02
1166.40	0.01
1166.81	0.22
1170.67	0.09
1171.67	0.12
1180.00	0.05
1180.83	0.02
1195.24	0.10
1195.34	0.12
1197.06	0.01
1199.87	0.00
1212.69	0.07
1213.70	0.01
1218.25	0.04
1219.31	0.05
1227.12	0.02
1227.70	0.01
1230.30	0.04
1233.47	0.02
1234.21	0.04

1238.31	0.01
1266.58	0.13
1271.41	0.10
1274.74	0.22
1281.18	0.18
1297.81	0.00
1298.24	0.00
1339.27	0.00
1345.64	0.01
1347.13	0.01
1348.62	0.01
1348.76	0.01
1349.14	0.00
1349.86	0.01
1349.97	0.01
1350.90	0.01
1352.17	0.01
1353.41	0.00
1353.89	0.00
1356.50	0.01
1357.85	0.00
1361.29	0.00
1361.64	0.00
1379.37	0.02
1379.75	0.03
1380.43	0.04
1381.55	0.02
1381.98	0.01
1382.30	0.02
1388.96	0.00
1394.08	0.01
1395.56	0.01
1411.35	0.01
1411.71	0.02
1411.82	0.01
1412.15	0.02
1415.33	0.02
1415.63	0.00
1419.28	0.01
1419.33	0.01
1422.69	0.10
1423.18	0.06
1424.21	0.06
1424.38	0.04
1427.21	0.03
1427.90	0.02
1429.17	0.06

1429.41	0.08
1429.80	0.04
1430.53	0.10
1430.96	0.06
1436.63	0.06
1437.02	0.04
1437.30	0.11
1439.78	0.12
1440.68	0.14
1441.92	0.11
1442.16	0.09
1442.99	0.04
1443.89	0.05
1444.56	0.03
1446.46	0.06
1446.71	0.13
1447.40	0.12
1447.96	0.01
1450.71	0.18
1455.31	0.00
1456.02	0.07
1457.32	0.01
1460.78	0.07
1462.29	0.17
1464.87	0.18
1464.99	0.04
1469.66	0.51
1470.99	0.23
1473.13	0.15
1475.08	0.14
1520.34	2.61
1522.56	2.82
1590.50	0.02
1590.69	0.03
1617.64	0.48
1617.82	2.06
1853.69	0.08
1860.43	0.08
1921.52	0.16
1932.57	0.25
2928.35	0.07
2928.53	0.09
2930.12	0.13
2937.88	0.02
2938.63	0.04
2939.45	0.05
2941.98	0.36

2942.19	0.10
2942.60	0.01
2943.69	0.02
2943.75	0.26
2945.11	0.08
2946.53	0.09
2950.48	0.18
2950.87	0.18
2951.01	0.03
2953.84	0.13
2954.64	0.36
3013.36	0.04
3013.85	0.03
3016.32	0.07
3016.58	0.02
3017.15	0.05
3020.60	0.11
3021.40	0.18
3022.52	0.05
3022.92	0.04
3026.62	0.05
3029.69	0.04
3030.25	0.01
3031.81	0.02
3032.86	0.02
3032.92	0.02
3034.09	0.04
3035.18	0.03
3036.54	0.01
3037.61	0.03
3039.23	0.10
3040.48	0.08
3040.56	0.07
3043.81	0.01
3043.84	0.02
3044.92	0.03
3045.72	0.03
3046.05	0.02
3046.43	0.02
3049.14	0.02
3053.62	0.03
3055.92	0.02
3057.59	0.01
3057.90	0.01
3059.12	0.01
3062.43	0.02
3065.13	0.04

3092.23	0.02
3092.37	0.02
3101.85	0.09
3102.35	0.09
3110.26	0.07
3111.36	0.09
3118.00	0.10
3119.35	0.16
3123.02	0.19
3129.66	0.07
3144.86	0.03
3146.85	0.02
3154.64	0.02
3156.03	0.02
3161.91	0.02
3167.04	0.03
3171.66	0.04
3175.22	0.10

4

-0.68158640844528	-4.71175720029462	-6.13577283168831	c
0.85519235971499	-5.79797831798439	-8.36581836069598	c
-3.54331879306182	-4.89022537589272	-6.69232269925228	c
-0.05310432575828	-6.21385539116751	-3.71703066384147	c
-0.00495300636498	-2.05168809558562	-5.61504199737464	n
0.30860407926856	-4.93910519825060	-10.19883326230057	h
0.51731654926690	-7.86834041799075	-8.50322466891223	h
2.91201340098446	-5.48419785360835	-8.07008775034720	h
-4.11478289673543	-6.89245721044531	-6.98610065578689	h
-4.03233127426932	-3.81297587008754	-8.42976446952149	h
-4.64645955130057	-4.11217454458197	-5.07994811650605	h
1.99001834096356	-6.06780184320256	-3.25352516726627	h
-0.55020225257387	-8.23798598804288	-3.98193954946972	h
-1.14124757902041	-5.47190853592371	-2.07811247822130	h
-0.00000000000000	0.00000000000000	-7.12144738347691	c
0.00000000000000	0.00000000000000	-2.65187436780161	si
0.00000000000000	-0.00000000000000	-9.93520947957312	c
0.00495300636498	2.05168809558562	-5.61504199737464	n
3.98985836842388	-0.00000000000000	-0.00000000000000	sb

-3.98985836842388	0.00000000000000	-0.00000000000000	sb
-2.27952730877104	0.33932251510698	-11.27226951284733	c
2.27952730877104	-0.33932251510698	-11.27226951284733	c
0.68158640844528	4.71175720029462	-6.13577283168831	c
0.00000000000000	-0.00000000000000	2.65187436780161	si
-4.04953941060981	0.63233116817799	-10.21656458603764	h
-2.27694619740754	0.33223363018809	-13.92500291366745	c
2.27694619740754	-0.33223363018809	-13.92500291366745	c
4.04953941060981	-0.63233116817799	-10.21656458603764	h
3.54331879306182	4.89022537589272	-6.69232269925228	c
0.05310432575828	6.21385539116751	-3.71703066384147	c
-0.85519235971499	5.79797831798439	-8.36581836069598	c
-0.00495300636498	2.05168809558562	5.61504199737464	n
0.00495300636498	-2.05168809558562	5.61504199737464	n
-4.06493077781560	0.59535612874349	-14.96145303892651	h
0.00000000000000	0.00000000000000	-15.25496944373040	c
4.06493077781560	-0.59535612874349	-14.96145303892651	h
4.03233127426932	3.81297587008754	-8.42976446952149	h
4.64645955130057	4.11217454458197	-5.07994811650605	h
4.11478289673543	6.89245721044531	-6.98610065578689	h
0.55020225257387	8.23798598804288	-3.98193954946972	h
1.14124757902041	5.47190853592371	-2.07811247822130	h
-1.99001834096356	6.06780184320256	-3.25352516726627	h
-2.91201340098446	5.48419785360835	-8.07008775034720	h
-0.30860407926856	4.93910519825060	-10.19883326230057	h
-0.51731654926690	7.86834041799075	-8.50322466891223	h
-0.00000000000000	0.00000000000000	7.12144738347691	c
-0.68158640844528	4.71175720029462	6.13577283168831	c
0.68158640844528	-4.71175720029462	6.13577283168831	c
-0.00000000000000	0.00000000000000	-17.33833123620273	h
0.00000000000000	0.00000000000000	9.93520947957312	c
-0.05310432575828	6.21385539116751	3.71703066384147	c
0.85519235971499	5.79797831798439	8.36581836069598	c
-3.54331879306182	4.89022537589272	6.69232269925228	c

0.05310432575828	-6.21385539116751	3.71703066384147	c
-0.85519235971499	-5.79797831798439	8.36581836069598	c
3.54331879306182	-4.89022537589272	6.69232269925228	c
-2.27952730877104	-0.33932251510698	11.27226951284733	c
2.27952730877104	0.33932251510698	11.27226951284733	c
1.99001834096356	6.06780184320256	3.25352516726627	h
-0.55020225257387	8.23798598804288	3.98193954946972	h
-1.14124757902041	5.47190853592371	2.07811247822130	h
0.30860407926856	4.93910519825060	10.19883326230057	h
0.51731654926690	7.86834041799075	8.50322466891223	h
2.91201340098446	5.48419785360835	8.07008775034720	h
-4.64645955130057	4.11217454458197	5.07994811650605	h
-4.11478289673543	6.89245721044531	6.98610065578689	h
-4.03233127426932	3.81297587008754	8.42976446952149	h
-1.99001834096356	-6.06780184320256	3.25352516726627	h
0.55020225257387	-8.23798598804288	3.98193954946972	h
1.14124757902041	-5.47190853592371	2.07811247822130	h
-0.30860407926856	-4.93910519825060	10.19883326230057	h
-0.51731654926690	-7.86834041799075	8.50322466891223	h
-2.91201340098446	-5.48419785360835	8.07008775034720	h
4.64645955130057	-4.11217454458197	5.07994811650605	h
4.11478289673543	-6.89245721044531	6.98610065578689	h
4.03233127426932	-3.81297587008754	8.42976446952149	h
-4.04953941060981	-0.63233116817799	10.21656458603764	h
-2.27694619740754	-0.33223363018809	13.92500291366745	c
2.27694619740754	0.33223363018809	13.92500291366745	c
4.04953941060981	0.63233116817799	10.21656458603764	h
-4.06493077781560	-0.59535612874349	14.96145303892651	h
0.00000000000000	-0.00000000000000	15.25496944373040	c
4.06493077781560	0.59535612874349	14.96145303892651	h
0.00000000000000	0.00000000000000	17.33833123620273	h

4.72	56.05
11.21	100.00
17.98	7.84
20.58	18.18

21.67	38.56
23.37	3.48
34.43	12.12
41.03	3.95
48.07	5.12
56.68	27.30
61.89	0.38
63.29	0.00
74.50	32.38
75.03	8.06
76.33	1.71
79.62	38.80
80.27	10.05
91.27	0.51
118.57	0.64
118.94	0.13
119.40	0.74
140.68	15.01
149.78	0.03
157.80	0.00
160.23	1.64
174.81	10.84
212.49	1.42
214.59	0.85
230.51	0.70
231.81	2.41
241.69	0.00
244.07	0.53
253.15	0.20
258.55	0.10
263.52	0.10
265.69	0.02
266.40	0.00
270.26	0.95
277.66	0.04
279.28	1.40
287.33	0.19
289.99	0.18
311.84	0.04
312.32	0.00
313.03	0.05
313.15	0.31
321.03	0.00
321.13	0.73
325.64	0.39
328.20	0.88
340.95	5.49

358.74	0.00
360.69	0.01
367.70	0.68
368.36	0.09
375.48	9.45
378.57	0.44
392.43	0.68
395.97	0.13
405.69	0.11
405.85	2.03
424.24	0.01
436.26	0.23
442.41	41.52
446.74	0.06
447.51	0.31
476.04	0.01
483.75	0.00
486.14	0.42
496.67	0.01
504.37	0.34
504.42	0.05
567.33	0.01
567.88	1.28
602.95	0.01
607.42	0.88
607.72	0.00
611.13	19.76
682.16	0.00
682.28	0.00
704.31	0.00
704.78	0.40
707.49	0.00
709.66	0.09
748.14	0.00
751.52	0.00
751.70	15.32
752.27	0.00
787.26	0.00
788.77	0.02
809.82	0.00
811.15	0.26
843.20	0.12
843.21	0.10
890.81	0.03
892.09	0.03
906.52	0.35
907.50	0.03

907.55	0.26
908.47	0.08
916.25	0.01
916.45	0.51
916.69	0.03
916.86	0.23
919.89	0.00
920.00	0.01
939.61	0.01
939.67	0.00
942.49	0.07
942.63	0.00
962.75	0.03
962.75	0.02
983.86	0.00
983.86	0.00
986.45	0.01
986.54	20.21
1015.38	0.00
1016.14	3.51
1022.88	0.00
1022.91	0.17
1024.54	0.66
1024.60	0.01
1028.15	0.01
1028.80	0.49
1032.38	0.02
1032.93	0.34
1066.30	0.02
1066.51	0.40
1075.78	0.00
1075.78	0.00
1151.30	0.02
1151.30	0.02
1164.98	0.00
1165.23	11.37
1170.63	0.00
1171.95	1.53
1199.16	0.01
1201.07	0.03
1211.61	0.03
1212.86	0.99
1215.01	0.24
1216.55	0.02
1229.92	0.00
1230.03	0.73
1230.22	0.00

1230.56	0.54
1265.05	0.01
1267.03	14.12
1295.55	0.03
1295.65	0.01
1346.77	0.04
1346.79	0.01
1346.98	0.10
1347.02	0.13
1349.43	0.25
1349.79	0.01
1353.59	0.00
1354.02	0.94
1362.64	0.00
1362.67	0.00
1379.53	0.39
1379.92	0.06
1383.36	0.01
1384.90	0.50
1412.42	0.00
1412.98	0.09
1413.21	0.05
1413.25	0.18
1422.28	1.12
1422.45	0.04
1422.87	0.02
1422.94	0.24
1428.93	0.69
1429.61	0.14
1431.45	0.04
1433.06	1.11
1437.48	0.12
1437.51	0.01
1443.60	0.04
1443.99	0.72
1444.52	0.55
1444.73	0.77
1444.87	0.15
1444.92	0.16
1445.47	0.07
1445.83	0.24
1457.30	0.05
1461.53	0.01
1461.88	0.14
1463.98	5.21
1464.47	0.35
1465.59	0.06

1468.66	0.09
1469.79	3.55
1518.73	0.11
1518.79	47.33
1591.36	3.09
1591.40	0.01
1617.37	0.15
1617.48	51.91
2935.92	0.11
2935.94	0.30
2935.95	0.12
2936.05	1.03
2941.15	0.02
2941.22	0.57
2942.05	1.85
2942.05	0.02
2948.04	0.35
2948.10	0.00
2948.17	0.00
2948.40	4.39
3026.31	0.02
3026.32	0.17
3026.43	0.01
3026.46	0.20
3028.65	0.05
3028.69	0.14
3028.89	0.00
3028.98	0.51
3031.74	0.00
3031.78	0.12
3032.05	0.01
3032.39	1.02
3033.17	0.73
3033.27	0.01
3033.30	0.06
3033.35	0.20
3040.70	0.00
3040.81	0.09
3040.86	0.21
3040.90	1.31
3054.26	0.19
3054.30	0.03
3054.48	0.00
3054.51	0.29
3091.51	0.00
3091.51	0.29
3100.17	0.83

3100.17	0.03
3108.76	0.00
3108.76	0.84
3115.26	0.26
3115.26	0.01
3120.70	0.03
3120.73	2.73

6

-3.98409867069716	2.23553472769210	-2.61322515501921	as
0.00659581102295	3.14988808457600	-4.46795784593879	as
-2.47425731520202	1.01730911037126	1.58951401219940	ge
-2.48608069854928	-0.92917992597273	-6.20698679628242	mo
2.82082817968145	1.27559041451666	-1.16143622449195	ge
1.90940379634840	-0.38905579410792	3.41916292503709	mo
-5.68663738017282	-0.49931008442106	3.20367521698110	n
-4.59483591895820	3.50427715093390	3.64632962950735	n
-5.65050699503006	1.11654221544214	-8.70675456103088	c
-3.77433231699878	0.10900522132700	-10.35581469870549	c
-3.80320884932807	-2.60703968358294	-10.09029923343824	c
-5.73956211960104	-3.29820490076258	-8.31613954727051	c
-6.84965600733248	-0.98019255021567	-7.44356992878362	c
0.99743419870109	-1.08629496282385	-7.47241557885367	c
-1.66068514728910	-3.67739654391264	-3.95787164232952	c
5.84554808787316	-0.11783220560696	-3.05008096858945	n
5.69197729607541	3.81796642376999	-1.71615244146804	n
0.78223367007186	-4.69232507064791	3.03214418579822	c
-0.51786073645611	-3.67972127953794	5.20285058405135	c
1.32921274730711	-2.97124013648550	7.03480275465063	c
3.79903118320966	-3.53386518569939	6.06963085122045	c
3.43294186120748	-4.60212364729384	3.57939299624258	c
5.19807616842429	1.33046664570446	3.27934705000802	c
1.16056560418597	2.40531784956731	5.68890496182521	c
-7.37491526229852	-2.65346224918895	2.56222114685222	c
-6.36572470583281	1.77498400906363	4.15014733127250	c
-4.62179785017809	6.28086593523281	4.02802028732156	c

-6.13346314862963	3.12121869181602	-8.46515148321263	h
-2.53230692247228	1.19454423099141	-11.61625266085736	h
-2.60449495809398	-3.92755640467899	-11.14828014808141	h
-6.71364703194237	-5.96347603136513	-7.80165733246335	c
-8.41472559589498	-0.82106449318987	-6.09163861838141	h
2.97764536149830	-1.17849940827517	-8.49096555254342	o
-1.25683913540728	-5.55937931056417	-2.78231388780688	o
6.94155017361365	-2.68605505743076	-3.30925392779991	c
7.06597677495126	2.12055794807868	-2.98319943048864	c
5.93527192222393	6.59688225635404	-1.54271332595255	c
-0.07990831425848	-5.44724553447750	1.29940394130147	h
-2.56262775680972	-3.47472720569018	5.45264342804659	h
0.91138631427618	-2.15377137017273	8.89553517800779	h
6.21553288746062	-3.51688724664207	7.64925472024934	c
4.93194194651066	-5.32345371599947	2.34612452812513	h
7.22709674502725	2.23247624772366	3.45353379931380	o
0.84775904079061	3.89422986517211	7.31429567784543	o
-5.71638088550289	-4.87738826328289	1.68458932733235	c
-9.11676926953684	-1.89431217399794	0.33996414111917	c
-8.96796045101898	-3.54443740297971	4.84541517105765	c
-8.67754782181531	2.21737828703330	5.71710348484089	c
-4.65623867574976	6.96687829907980	6.86324840386191	c
-2.20269542989302	7.31616054458681	2.78263289757492	c
-6.91382977536620	7.47715128689245	2.65996653850963	c
-8.85043700687100	-6.46818615964610	-9.74619636423362	c
-7.79916329393015	-6.17637415505243	-5.10555057065138	c
-4.60948170539966	-7.95312279489071	-8.15183983444634	c
4.71682534037568	-4.55989822462673	-3.25188635369255	c
8.39987175671135	-3.05567244206487	-5.81233703167824	c
8.71567827815034	-3.17245841552676	-1.04191574827697	c
9.52461889469979	2.61192056491766	-4.26870190562658	c
3.73469011938529	7.47993490291423	0.14704011229209	c
8.45489664599307	7.36216592510532	-0.27582101794736	c
5.71088842314592	7.83451360444683	-4.17701844423116	c

8.60938584599140	-3.68415884986356	5.99709422933204	c
6.07760412706972	-5.90411474609814	9.35297856485918	c
6.36722153854074	-1.13875777693098	9.33540546358539	c
-4.69863010808066	-5.78239844687945	3.27756592278900	h
-6.91870628337060	-6.35182189036093	0.80007337372272	h
-4.31788175659077	-4.26622013960952	0.25068939711856	h
-7.96491375908582	-1.41159894772221	-1.35057417378321	h
-10.39282419551421	-3.48505120363063	-0.17005272062633	h
-10.30611959479333	-0.24093600375801	0.83713025900729	h
-10.49640575591623	-2.20154316833280	5.34515247014883	h
-9.86316943431390	-5.38928732099937	4.38199092510055	h
-7.75094658532543	-3.81269252263294	6.53796838401186	h
-10.99577949708005	3.01579002678524	4.67887885040524	c
-8.50731550412214	1.74703500929266	8.33606579230169	c
-3.03282122562595	6.08146395996927	7.85071386886721	h
-4.51453340229720	9.05249758600453	7.08988565727857	h
-6.43810220079154	6.34276443467703	7.78000005260372	h
-2.18736291557493	6.87573221396941	0.72832222495769	h
-2.11602635237859	9.40308174374728	2.99937407414391	h
-0.48263429330080	6.50864766000110	3.65647907402139	h
-8.71569495954322	7.04629382711819	3.63976263327014	h
-6.70431772005160	9.56841916040821	2.61627870669280	h
-7.02398465002098	6.77921862267571	0.67974541395068	h
-8.12466601072920	-6.32665609873553	-11.71467706196403	h
-9.65221824833109	-8.39628832197479	-9.47861766847358	h
-10.40909614778898	-5.07247801345532	-9.53302363329462	h
-9.40931000294825	-4.85992789060807	-4.80768382714429	h
-8.50808063207850	-8.12607639649739	-4.75653473455844	h
-6.32961251195306	-5.74008799372925	-3.67732721180632	h
-3.02087076529191	-7.61812518783040	-6.82373897523724	h
-5.37673772544039	-9.87835303462544	-7.79168345638252	h
-3.85001091277536	-7.94266923673331	-10.11149302913875	h
3.44541633414681	-4.19243438704931	-1.62649729141536	h
3.55904563293389	-4.41223432246055	-4.98766931172406	h

5.42432820656122	-6.53133936055239	-3.09838179062834	h
8.84085019369937	-5.09832751809925	-6.04821388179741	h
7.22676912503096	-2.43840258787431	-7.43528799089774	h
10.21471568719730	-2.00688791101229	-5.83959728044921	h
10.39989513854182	-1.92097154937484	-1.12311625587272	h
7.71487202711694	-2.78533636123182	0.75646681263553	h
9.39039789108612	-5.16441658563468	-1.02716617755928	h
9.55802952160811	2.91521247563355	-6.91643616721858	c
11.79951421165129	2.72272786703645	-2.88974979503738	c
3.79075246304576	9.56323557319853	0.40456148578128	h
1.88827440013977	6.98747416343883	-0.72233875566420	h
3.83884339994351	6.57219609249565	2.03796686930755	h
8.72578830884000	6.29678326846328	1.51118318440198	h
10.09420277916760	7.00347617176923	-1.53261463979511	h
8.42671124339067	9.41845036614357	0.16166545353024	h
3.90809413202554	7.27718639287757	-5.10215903580112	h
5.74935552839122	9.92975286325633	-3.99930686279248	h
7.30436244418876	7.26156696806943	-5.41876600257402	h
8.60788188236464	-5.40388272494592	4.79007824250655	h
8.81105692153733	-2.00570993739608	4.75928603667921	h
10.31382324907602	-3.78196753380913	7.22437880770016	h
7.79911392290094	-6.04589721873487	10.55478963860310	h
4.39991363023631	-5.83865419519391	10.61831731086666	h
5.94269659724883	-7.65194397322418	8.19178078032806	h
6.46634944242818	0.60722039315829	8.17726681636984	h
4.70643313124050	-0.97305070106764	10.61129350936817	h
8.08425944113035	-1.22514264069807	10.54645706613541	h
-11.13565249530935	3.40128071090949	2.63959301423412	h
-13.12196413915660	3.31851174106707	6.23857590165945	c
-10.63160264964615	2.05629340206097	9.89235286915305	c
-6.68793743555383	1.12994980565738	9.13921903637821	h
7.77000745441168	2.78039846623826	-7.97487097452350	h
11.85506005494125	3.34106485014577	-8.17101432275938	c
14.09748807341457	3.12494778173400	-4.15568808560319	c

11.74908539438544	2.49563325997033	-0.82121276656537	h
-14.92966103292118	3.93767145957312	5.40863382474189	h
-12.94643681963052	2.83547333485047	8.84488637846407	c
-10.48007854273667	1.68376362255854	11.93647706503533	h
11.87423833693350	3.58151586092478	-10.24033015181218	h
14.12734678890191	3.43948915316452	-6.79428822511908	c
15.87599071327251	3.19841965800484	-3.07301090062521	h
-14.61835615179958	3.07213269631659	10.06527059183490	h
15.93248512673060	3.76051964350657	-7.78442989711122	h

8.78	100.00
15.32	54.72
23.71	16.44
24.97	9.67
29.99	6.37
34.47	3.43
40.39	4.87
42.71	4.95
45.50	1.08
50.81	0.48
56.96	0.99
59.00	9.01
59.96	0.48
64.17	2.82
66.35	5.37
67.16	7.84
72.17	1.96
74.30	5.32
75.98	6.78
78.01	3.46
79.77	3.91
84.43	6.87
86.47	3.03
88.01	5.49
93.60	0.32
95.82	2.84
97.02	1.01
100.73	0.98
105.28	2.53
109.33	1.93
113.92	1.11
116.77	1.14
122.69	0.43

124.26	2.87
127.63	0.73
129.06	0.10
131.31	0.68
134.23	1.33
139.51	0.78
142.60	1.28
145.91	0.90
150.16	0.38
151.46	0.18
155.49	0.36
163.65	0.43
165.25	0.72
173.32	0.51
177.72	0.98
185.46	0.33
192.09	0.54
192.89	0.16
203.89	0.04
212.37	0.20
217.20	0.22
217.73	0.03
227.57	0.30
231.72	0.13
232.31	0.26
233.86	0.08
237.68	0.09
243.53	0.10
247.77	0.44
247.94	0.31
250.41	0.22
251.19	0.02
253.75	0.25
259.47	0.19
265.66	0.15
269.30	0.54
270.13	0.17
271.63	0.31
275.71	0.09
280.11	0.12
284.46	0.14
286.99	0.06
289.04	0.20
290.13	0.40
291.29	0.04
292.42	0.22
297.40	0.06

300.36	0.05
303.85	0.69
307.66	0.02
310.73	0.12
312.35	0.02
320.37	0.32
323.72	0.31
326.11	0.09
329.23	0.07
332.26	0.23
337.27	0.06
343.56	0.79
353.19	0.02
355.13	0.10
363.45	0.29
369.03	0.39
376.14	0.09
377.78	0.03
378.86	0.11
386.34	0.12
388.98	0.22
390.14	0.08
394.72	0.13
396.74	0.07
400.86	0.04
405.25	0.12
409.73	0.04
409.92	0.19
443.25	0.09
444.35	0.03
456.44	0.10
456.75	0.03
459.98	0.10
461.64	0.21
462.77	0.06
468.63	0.08
471.66	0.03
477.09	0.08
479.12	0.07
481.93	0.17
483.08	0.13
489.27	0.09
501.58	0.12
501.93	0.08
506.41	0.78
514.18	0.97
527.33	0.82

539.08	0.30
557.83	0.28
559.66	0.95
560.39	0.26
566.62	0.15
571.87	0.07
575.57	0.08
577.25	0.13
581.10	0.28
590.45	0.03
591.92	0.08
600.92	0.15
607.17	0.16
608.18	0.15
615.03	0.07
668.97	0.16
671.36	0.03
674.07	0.02
679.09	0.14
704.25	0.05
704.56	0.16
707.09	0.08
707.79	0.05
738.95	0.04
742.65	0.12
744.59	0.01
750.72	0.01
779.15	0.08
783.57	0.02
788.52	0.01
796.57	0.05
800.91	0.05
805.90	0.06
809.61	0.25
809.82	0.02
810.56	0.14
813.64	0.14
815.27	0.03
822.50	0.04
835.42	0.07
840.12	0.06
845.94	0.02
869.55	0.01
872.94	0.00
882.82	0.03
889.74	0.02
895.83	0.05

899.62	0.06
901.48	0.09
904.46	0.06
906.64	0.07
908.17	0.06
911.66	0.09
914.63	0.03
915.39	0.04
916.41	0.05
916.97	0.03
917.63	0.10
917.98	0.17
918.66	0.16
923.56	0.00
929.31	0.06
930.51	0.05
937.88	0.01
939.58	0.01
944.24	0.01
945.65	0.00
946.94	0.00
953.73	0.00
959.48	0.01
965.40	0.02
982.34	0.06
984.45	0.00
986.08	0.77
986.31	1.29
1007.37	0.01
1011.36	0.17
1012.74	0.02
1013.73	0.96
1016.62	0.02
1017.28	0.05
1018.78	0.14
1019.79	0.06
1023.59	0.07
1025.57	0.30
1026.74	0.09
1026.92	0.06
1028.11	0.04
1029.29	0.12
1030.56	0.05
1033.57	0.05
1036.05	0.12
1042.97	0.10
1045.24	0.05

1053.38	0.07
1055.20	0.15
1061.90	0.03
1074.39	0.00
1075.06	0.00
1142.65	0.81
1149.81	0.41
1150.21	0.07
1150.73	0.08
1160.67	0.66
1163.04	0.31
1167.47	0.03
1168.43	0.28
1181.99	0.01
1184.26	0.07
1187.31	0.05
1192.80	0.30
1193.59	0.05
1196.21	0.27
1205.45	0.19
1210.62	0.12
1212.87	0.25
1217.85	0.10
1226.45	0.17
1228.85	0.01
1230.63	0.09
1231.26	0.17
1232.54	0.14
1233.71	0.04
1236.90	0.19
1246.94	0.15
1265.14	0.15
1272.91	0.14
1294.08	0.01
1295.98	0.01
1344.01	0.02
1345.93	0.03
1346.94	0.02
1347.49	0.04
1348.14	0.03
1348.40	0.05
1348.70	0.02
1350.99	0.03
1353.46	0.02
1354.46	0.01
1354.78	0.06
1356.81	0.01

1361.54	0.00
1362.53	0.00
1363.36	0.03
1363.65	0.04
1376.37	0.07
1377.27	0.05
1380.13	0.05
1380.97	0.05
1381.93	0.06
1384.08	0.13
1384.97	0.09
1390.26	0.06
1399.34	0.10
1410.05	0.02
1415.41	0.02
1416.56	0.02
1417.41	0.02
1418.01	0.02
1418.72	0.03
1419.23	0.04
1421.73	0.07
1423.50	0.18
1423.92	0.13
1424.02	0.36
1428.21	0.21
1428.87	0.24
1430.41	0.08
1430.63	0.16
1431.16	0.09
1431.70	0.08
1434.49	0.27
1436.04	0.39
1436.66	0.02
1437.55	0.74
1438.20	0.36
1440.10	0.47
1442.20	0.20
1442.49	0.21
1443.73	0.36
1444.43	0.24
1446.13	0.08
1446.43	0.11
1447.58	0.27
1447.75	0.15
1447.87	0.34
1451.47	0.10
1451.98	0.10

1454.61	0.50
1458.83	0.02
1460.39	0.06
1462.79	0.03
1465.11	0.12
1466.28	0.03
1471.55	0.10
1478.39	0.34
1479.41	0.04
1480.36	0.29
1481.37	0.05
1514.93	3.18
1524.01	1.79
1591.16	0.06
1591.34	0.05
1617.05	3.27
1617.39	2.04
1818.47	1.33
1889.52	0.22
1914.35	0.43
1939.74	0.23
2927.72	0.28
2931.48	0.27
2939.32	0.31
2942.89	0.11
2943.87	0.23
2944.41	0.06
2947.06	0.05
2947.25	0.06
2948.13	1.04
2948.17	0.03
2950.00	0.09
2951.26	0.73
2952.88	0.26
2953.84	0.36
2954.93	0.48
2958.05	0.97
2971.68	0.34
2972.82	0.27
3013.45	0.14
3017.26	0.04
3018.41	0.14
3021.45	0.31
3022.00	0.05
3022.84	0.10
3025.43	0.21
3026.27	0.34

3026.62	0.10
3026.87	0.04
3027.16	0.11
3030.41	0.13
3032.93	0.05
3033.66	0.06
3036.63	0.05
3037.62	0.15
3038.36	0.15
3040.71	0.19
3041.97	0.17
3044.59	0.13
3050.77	0.06
3057.09	0.04
3057.25	0.04
3057.31	0.14
3057.38	0.04
3059.60	0.04
3060.56	0.09
3064.29	0.05
3068.18	0.05
3069.73	0.04
3073.96	0.09
3075.09	0.03
3076.21	0.07
3083.95	0.06
3089.80	0.07
3090.41	0.05
3091.16	0.05
3091.64	0.05
3099.50	0.26
3099.75	0.24
3107.49	0.20
3108.29	0.18
3114.52	0.03
3115.85	0.23
3119.42	0.71
3121.77	0.58
3144.40	0.03
3144.45	0.06
3155.77	0.09
3157.13	0.09
3163.94	0.06
3168.97	0.25
3183.00	0.02
3184.34	0.13

7

-4.52912455163702	2.13096685846000	-3.21726630949481	sb
0.09055784440932	3.22765963570693	-5.35063490729733	sb
-2.58870945885932	1.03402536488797	1.30744550614091	ge
-2.56557770555551	-1.24826491190914	-6.94470530074204	mo
2.84411526024230	1.38081791066595	-1.42953170719163	ge
1.85560675549380	-0.30794053715088	3.16261909189266	mo
-5.66336140550844	-0.54837741433134	3.13604564467793	n
-4.65908374118684	3.48651829992522	3.46685995796461	n
-5.30599031354462	0.57022035965399	-10.09505616155525	c
-3.46488218145258	-1.01221940921037	-11.27769194295736	c
-3.87480172285104	-3.57414417583831	-10.43422402003219	c
-5.99997191260226	-3.59074875564012	-8.73222064717457	c
-6.85048824785276	-1.02006205311227	-8.52575362950200	c
0.95965711310750	-1.45816633163070	-8.05708182095755	c
-1.73795851594452	-3.78183202656380	-4.44552819449741	c
6.03265115802272	0.03398755091459	-3.06207155354263	n
5.70878224910520	3.96999883695139	-1.78351756062576	n
0.77202219329136	-4.61916535220377	2.76977676098800	c
-0.56090879548977	-3.61828511195554	4.92748814537184	c
1.25401646529380	-2.89154426305554	6.78060130925923	c
3.73935862239164	-3.43604355849769	5.84877734305153	c
3.41457967242435	-4.50526170811736	3.35237029319054	c
5.17252766477277	1.34545448639673	3.04616873200888	c
1.11762715021992	2.50114238617994	5.41418796950635	c
-7.32963906207571	-2.76503639535420	2.67296705295441	c
-6.31684884490374	1.70320663522432	4.14687643987780	c
-4.73005329131587	6.26130326864060	3.85728546375696	c
-5.53830200701698	2.61476920524404	-10.36845905253327	h
-2.01474126515151	-0.39812337186690	-12.63104818645668	h
-2.81365668161511	-5.23601293485980	-11.07327934670472	h
-7.38264356765272	-5.91068933816620	-7.71893506591117	c
-8.48907337052240	-0.37835441084759	-7.42642776692050	h

2.95739388170090	-1.68912683097235	-9.02782723136960	o
-1.28754188211022	-5.50874796615191	-3.07238356310602	o
7.08698211427895	-2.53163967496258	-3.44162482950238	c
7.23246806063847	2.27313217134970	-2.87504167490600	c
5.95945278503528	6.73693469678269	-1.49152330514110	c
-0.06560487380729	-5.37451685955083	1.02480614047258	h
-2.60718284502164	-3.42484307235953	5.15921898490871	h
0.80509021793206	-2.07789741811180	8.63578109619102	h
6.12745232699210	-3.41529095845829	7.47176145920375	c
4.93910863194172	-5.20966038576067	2.14062797672112	h
7.23145472658031	2.17541057623020	3.24637089328142	o
0.81971462070797	3.99883750061349	7.03577009099280	o
-5.73216804173501	-4.85509936007497	1.42020375888951	c
-9.46509735396974	-2.01731980467398	0.82098898753688	c
-8.47667505919710	-3.82577707464661	5.14331829987953	c
-8.44352315842982	2.06683260265409	5.97543296918500	c
-4.72396003357547	6.96487954669470	6.68954543761511	c
-2.35852146199188	7.35168306525820	2.56832637533613	c
-7.08123244380340	7.39427056254331	2.53752887008172	c
-9.57625322084618	-6.48005614846418	-9.57918775503080	c
-8.47267042879446	-5.37948990852652	-5.06976161554811	c
-5.61772173319838	-8.22797843633808	-7.58431365242616	c
4.82805231700827	-4.36919486932436	-3.44961123910128	c
8.50002705614548	-2.77860823561613	-5.98405588293231	c
8.87382129800128	-3.17586047047924	-1.22469234856706	c
9.85174395596492	2.77702136080612	-3.78839267796239	c
3.65329431604525	7.57763290629700	0.07477533593239	c
8.38674621153574	7.47229671942984	-0.03782449046256	c
5.92710732676106	8.05868386501010	-4.09483204046988	c
8.54963862871862	-3.63594035951232	5.86855729266048	c
5.93412257670345	-5.77435062856661	9.20961596224510	c
6.27124771656411	-1.01344765594347	9.12499556072696	c
-4.45908238116559	-5.80914929466159	2.78244632173113	h
-6.99212354736840	-6.33022946719083	0.62230910356346	h

-4.57054938224231	-4.10118602272972	-0.15283214840843	h
-8.65248317241028	-1.29184638165971	-0.97545500622274	h
-10.66300696595331	-3.68272986800392	0.36339056519337	h
-10.70123368909086	-0.53768419326409	1.64298552436993	h
-9.92244293421045	-2.55239470607433	5.96843988016325	h
-9.39782945775325	-5.67325892127214	4.74519938599453	h
-6.98114170839856	-4.13937247168484	6.58696230464474	h
-10.88124481173364	2.85146091786613	5.24910871371164	c
-7.94491195127816	1.55163813827032	8.54497732277197	c
-3.09550844034070	6.07380625990737	7.66302755591000	h
-4.55791287563764	9.05072937561386	6.89504326210360	h
-6.49748443493127	6.36930745239270	7.63866286594871	h
-2.33587627363011	6.86509749635868	0.52351500025790	h
-2.34688931465321	9.44511246373451	2.73245097313806	h
-0.60765844737164	6.62706965969317	3.45230406770324	h
-8.85157961025875	6.84813679997140	3.51785925798201	h
-6.97041194848167	9.49360742783697	2.55435705415044	h
-7.18147253030154	6.75090656309048	0.53843323971322	h
-8.84156163874957	-6.85792195940412	-11.51289541739102	h
-10.66268006972906	-8.16937583101840	-8.94791617100118	h
-10.90417997028627	-4.85392833488354	-9.70230876819635	h
-9.86159208587965	-3.80259336792490	-5.09394123097388	h
-9.46299722706212	-7.07884180084607	-4.32217707252688	h
-6.93843350029251	-4.85980242161497	-3.74147119000501	h
-3.99028044405702	-7.87498971066061	-6.30890402790782	h
-6.67617698013507	-9.89361644133121	-6.85581454386121	h
-4.87332212138746	-8.74479414377185	-9.48063216013169	h
3.59935617500287	-4.08325580567817	-1.77535235660738	h
3.63915677714725	-4.09770756793535	-5.14691144268344	h
5.50197441675258	-6.35783449089872	-3.43561012512721	h
8.95911659506230	-4.80186138308007	-6.32693299582198	h
7.28561067367713	-2.10487669244102	-7.55502865325231	h
10.30137587912404	-1.70537469082896	-5.99223351875084	h
10.60886597202320	-1.99627352402838	-1.27858587351273	h

7.91195437717894	-2.82579347563713	0.60263792661017	h
9.46352971356685	-5.19275228340981	-1.30455003031100	h
10.29062742887251	3.26606017474733	-6.36969813628340	c
11.89386092964555	2.75154517564768	-2.07768009949545	c
3.67955131921777	9.65547681650368	0.37687777175355	h
1.86233680040031	7.09315855325668	-0.91033600182544	h
3.64873034719808	6.63031210889158	1.94885021291089	h
8.54943741179833	6.36190557179437	1.73346992165840	h
10.11040252332490	7.15912059610521	-1.18863688834491	h
8.31263253313568	9.51548094715507	0.45244440222110	h
4.17851296900920	7.57234853460479	-5.15453893052903	h
5.99810563811326	10.14675415277655	-3.85363271499318	h
7.58627754111046	7.48288714759590	-5.24667715528661	h
8.55392322346593	-5.38007737415596	4.69696444247634	h
8.78915813083079	-1.98468422742981	4.60192606654178	h
10.23020021027084	-3.72378950045983	7.12911274638519	h
7.63578334358432	-5.91827341568368	10.43914926282418	h
4.23908985967646	-5.66948210560559	10.44882860283556	h
5.79511141517958	-7.53819453075705	8.07337643795426	h
6.42532993761165	0.71289673981397	7.94436757368994	h
4.58057519173422	-0.80422908246853	10.35431699543014	h
7.95558174907566	-1.10580047739520	10.38087328394457	h
-11.28283261098511	3.26500532221974	3.25041987257196	h
-12.79925748394612	3.10343399846007	7.06792583473351	c
-9.85992078390456	1.81050589514859	10.35914676579781	c
-6.03131466233905	0.94899017511942	9.10283875872250	h
8.68644864517978	3.25067428780114	-7.69686752091865	h
12.75620359514093	3.73257277106781	-7.23156668332139	c
14.35942929369923	3.19954276669882	-2.94968486012365	c
11.51954428135497	2.39875940187775	-0.06074360609054	h
-14.70263266934168	3.71347105155653	6.48061030222300	h
-12.29475449261059	2.58215144927165	9.62276044447338	c
-9.45033598306259	1.40678767506164	12.36149156393095	h
13.09028189643994	4.11702062497448	-9.25168456265660	h

14.79332625899986	3.69378114708135	-5.52489223961639	c
15.95210770842614	3.17154086148330	-1.60682882935421	h
-13.80283011790660	2.78137721041065	11.04635671579745	h
16.72998421804381	4.05097406031344	-6.20578706488436	h

11.08	100.00
12.81	31.23
20.34	27.32
24.22	7.84
26.06	8.76
31.90	4.43
38.55	9.22
41.36	2.37
46.58	0.91
52.08	1.33
55.47	5.64
56.74	2.99
60.11	11.30
61.57	4.30
63.99	7.05
66.65	1.47
69.70	3.18
71.99	5.83
73.97	7.30
75.76	7.23
76.34	1.41
80.94	4.42
81.50	10.58
83.04	0.66
86.52	2.09
93.92	3.28
97.30	0.86
100.26	0.97
105.27	4.98
105.77	1.31
107.74	0.75
109.91	2.51
114.22	0.31
115.73	2.30
119.89	3.41
122.52	1.52
126.11	0.44
129.19	1.31
132.30	0.88
135.56	0.13

141.28	0.66
144.17	0.22
146.50	0.66
151.26	0.21
156.01	0.37
162.23	0.28
169.41	0.68
171.29	0.51
174.94	0.46
185.42	0.13
187.89	0.36
203.23	1.48
204.97	0.49
206.72	0.20
210.28	0.10
217.83	0.03
224.59	0.12
227.17	0.10
228.04	0.25
231.64	0.07
235.16	0.11
239.31	0.15
239.96	0.08
244.74	0.65
247.12	0.37
249.62	0.23
249.92	0.09
252.33	0.20
256.39	0.46
263.19	0.31
266.86	0.14
270.65	0.32
274.06	0.20
274.68	0.14
280.12	0.05
282.12	0.09
286.44	0.11
290.93	0.01
294.85	0.03
295.79	0.10
301.62	0.02
305.79	0.12
306.95	0.65
308.98	0.06
310.73	0.03
317.78	0.19
317.99	0.13

318.82	0.19
322.19	0.21
330.37	0.16
335.04	0.05
342.03	0.81
355.33	0.01
355.87	0.12
362.88	0.28
366.79	0.37
374.49	0.06
376.89	0.03
379.28	0.11
383.40	0.13
389.99	0.21
392.45	0.12
395.54	0.12
401.25	0.05
402.48	0.07
405.59	0.09
409.40	0.12
409.56	0.09
443.38	0.09
445.52	0.04
453.43	0.05
455.89	0.00
456.41	0.07
460.19	0.17
465.29	0.10
467.91	0.05
469.90	0.04
475.82	0.09
478.08	0.04
480.94	0.18
481.70	0.09
482.83	0.08
501.91	0.14
502.25	0.07
505.88	0.71
519.54	1.42
525.64	0.33
541.25	0.25
556.86	0.20
557.46	0.81
558.31	0.50
566.47	0.20
572.32	0.07
575.05	0.10

576.82	0.10
581.55	0.30
590.09	0.06
592.24	0.06
600.15	0.14
607.30	0.13
608.06	0.15
615.39	0.08
665.19	0.06
669.57	0.04
673.78	0.02
678.71	0.09
703.50	0.07
704.84	0.11
706.62	0.09
708.83	0.05
737.65	0.04
742.81	0.11
744.68	0.04
750.10	0.01
773.48	0.13
783.55	0.02
787.37	0.02
791.90	0.08
799.02	0.05
800.45	0.08
809.57	0.01
810.41	0.15
811.13	0.26
813.34	0.15
816.07	0.03
823.88	0.06
839.38	0.06
842.39	0.02
846.90	0.02
868.22	0.01
881.00	0.02
883.29	0.02
892.62	0.03
893.88	0.05
896.98	0.07
900.55	0.06
903.47	0.06
905.83	0.02
906.54	0.10
909.95	0.10
914.00	0.03

914.58	0.03
915.51	0.04
916.98	0.03
917.51	0.08
917.62	0.15
919.47	0.20
924.70	0.00
929.44	0.05
931.16	0.05
936.29	0.02
938.79	0.01
943.06	0.01
945.01	0.01
945.55	0.00
952.25	0.01
959.01	0.01
966.86	0.02
982.23	0.04
985.44	0.11
985.76	1.06
986.72	0.93
1009.14	0.01
1011.34	0.18
1013.53	0.04
1013.99	0.93
1015.97	0.03
1017.82	0.06
1018.81	0.10
1020.47	0.03
1022.12	0.07
1025.25	0.23
1026.88	0.14
1027.77	0.09
1027.99	0.04
1028.54	0.06
1030.11	0.09
1033.83	0.03
1034.12	0.10
1041.66	0.09
1046.82	0.04
1052.37	0.05
1057.75	0.13
1065.09	0.04
1074.35	0.00
1075.56	0.01
1143.20	0.98
1150.21	0.07

1150.83	0.13
1150.88	0.36
1160.50	0.67
1163.72	0.33
1168.02	0.04
1168.38	0.26
1181.39	0.03
1185.22	0.09
1186.60	0.08
1192.77	0.27
1194.01	0.05
1197.56	0.29
1204.60	0.18
1209.48	0.10
1212.53	0.27
1216.11	0.12
1225.26	0.17
1228.78	0.01
1229.88	0.12
1230.60	0.12
1232.19	0.19
1234.93	0.07
1235.66	0.13
1250.44	0.18
1266.68	0.20
1273.86	0.14
1294.75	0.01
1296.96	0.01
1344.06	0.02
1345.95	0.03
1347.04	0.04
1347.20	0.05
1349.19	0.02
1349.31	0.03
1349.52	0.02
1351.26	0.03
1353.58	0.02
1354.68	0.02
1356.22	0.05
1357.66	0.02
1360.39	0.03
1361.42	0.00
1361.83	0.03
1362.35	0.00
1376.75	0.05
1377.71	0.08
1380.28	0.03

1381.66	0.04
1382.79	0.10
1384.75	0.15
1389.39	0.08
1389.99	0.06
1395.39	0.11
1410.91	0.03
1414.11	0.04
1414.90	0.02
1417.57	0.01
1417.99	0.02
1418.35	0.04
1420.54	0.02
1421.93	0.19
1423.72	0.20
1424.30	0.11
1424.39	0.07
1428.93	0.29
1429.01	0.12
1429.29	0.03
1429.47	0.14
1431.53	0.07
1433.14	0.24
1434.47	0.09
1435.14	0.42
1436.49	0.10
1437.33	0.08
1437.61	0.76
1440.25	0.41
1441.43	0.17
1442.83	0.20
1443.73	0.55
1445.13	0.23
1445.77	0.05
1446.42	0.23
1447.12	0.02
1448.71	0.61
1449.60	0.23
1451.26	0.17
1455.61	0.12
1458.41	0.16
1459.30	0.06
1459.81	0.25
1462.91	0.08
1464.35	0.12
1466.99	0.07
1470.52	0.12

1478.23	0.32
1478.65	0.05
1479.48	0.33
1481.18	0.04
1512.57	3.27
1521.03	2.14
1591.14	0.06
1591.40	0.05
1616.92	3.17
1617.80	2.14
1826.62	1.24
1882.96	0.33
1902.66	0.59
1933.85	0.28
2927.34	0.29
2931.70	0.27
2938.02	0.36
2941.39	0.02
2943.74	0.28
2944.65	0.12
2945.56	0.35
2946.65	0.19
2947.15	0.04
2948.87	0.75
2950.83	0.62
2951.91	0.14
2953.40	0.01
2954.97	0.42
2955.60	0.45
2957.01	1.21
2974.74	0.25
2975.17	0.33
3012.58	0.13
3015.61	0.01
3018.32	0.13
3019.41	0.30
3021.68	0.07
3022.59	0.11
3025.45	0.33
3025.56	0.06
3025.96	0.12
3026.33	0.22
3026.58	0.05
3031.63	0.09
3031.85	0.12
3035.58	0.09
3036.01	0.03

3038.73	0.03
3039.00	0.22
3039.29	0.16
3040.88	0.15
3044.62	0.19
3055.40	0.06
3057.34	0.08
3057.43	0.07
3059.09	0.06
3059.45	0.04
3059.62	0.03
3061.32	0.12
3069.16	0.04
3070.16	0.05
3070.61	0.03
3071.16	0.11
3071.34	0.03
3076.23	0.07
3077.64	0.05
3089.78	0.07
3091.12	0.05
3093.86	0.04
3099.38	0.25
3099.60	0.24
3104.78	0.04
3107.62	0.21
3108.06	0.18
3114.80	0.05
3115.51	0.21
3119.55	0.70
3121.45	0.62
3143.70	0.07
3144.17	0.01
3152.75	0.08
3156.61	0.09
3163.30	0.12
3171.57	0.24
3180.25	0.05
3193.79	0.09

8

4.87888804349135	22.92736421289526	10.28709895201477	sb
3.69501533989155	25.29135562712056	15.03199103936550	sb
1.68860100650521	18.75136982967052	11.61972805807211	sb
1.54369245988010	25.96793207478057	7.10085324547542	mo

7.01269962129138	29.31409227772297	13.51470349087191	sb
6.98705638535417	22.15419506575374	18.18990496851433	mo
3.73501897269717	19.05562056457067	16.27217277783881	ge
4.11202657285408	14.53919429766685	9.68162069369061	ge
4.87674435868171	29.03105714192021	8.89787810139834	ge
-1.88284045382562	24.87868347087760	9.92742455118387	c
-1.79921970557728	27.56939260340810	9.74529702610029	c
-2.65172811340749	25.95995561079293	5.70628788778433	c
-2.37959254975024	23.88663083601960	7.45877816157770	c
-2.26894581914743	28.23919047801741	7.16034165701637	c
2.91353763896517	27.81295006002325	4.23084764548591	c
3.01995209375801	23.23576001725531	5.12739027364270	c
4.77252340079474	33.64052822680129	15.40075695324009	ge
10.37894199619735	23.30310091208427	15.38591662360996	c
10.32115747700175	20.60951618849551	15.52359432100023	c
11.18928734337408	22.15822968507798	19.58100504242283	c
10.89232676242670	24.25766529189955	17.86538786200652	c
10.81557095795611	19.90136502783420	18.09235163023589	c
5.58599670644965	20.26928535077180	21.02051046807169	c
5.48794701026217	24.84455815947777	20.20494687432233	c
0.76810456987271	18.09077518058665	18.50653790116426	n
3.85422202257798	15.45615422003488	17.65655571852239	n
6.49009125672598	15.94160192498602	6.93424232886472	n
2.65830733875570	14.63682073038425	6.08973797359451	n
7.81061667953381	29.93936295478039	6.58224694290961	n
4.78506216925747	32.62376419743690	7.51139053161494	n
-1.61755272510791	23.76337826643007	11.65776350410061	h
-1.47842289988036	28.87194599705293	11.32483501866018	h
-3.65137692998196	25.78590863386405	3.00340938161281	c
-2.59669615451456	21.87345285866528	7.00968369170873	h
-2.40246461745461	30.17127142087378	6.42366843273957	h
3.65863477549687	28.87137582863116	2.40540848242575	o
3.79930857614973	21.62269137988516	3.79465133132098	o
2.43431834974723	32.36922308978532	18.27056856863209	n

6.33169459520894	33.58871866072131	18.94821197307821	n
10.08769182287894	24.44752953728228	13.68016682691989	h
9.99608991042542	19.33436222095140	13.92527325640172	h
12.20148378495638	22.29503054526237	22.27941779735026	c
11.09951424496963	26.26359792528858	18.34504180970804	h
10.96936457169572	17.95827282956925	18.79385138299157	h
4.81779565251785	19.19346734632028	22.82543490480831	o
4.69624823437845	26.42846172748821	21.56713441461143	o
1.69882723372029	15.78044213187328	18.96643477676561	c
-1.67136374619348	19.23762063121195	19.21520190724745	c
5.81948931869600	13.48394069861908	17.86923383506693	c
9.21930744432693	16.50936673600902	6.86890461323708	c
4.89362077846497	15.30268811406277	5.06478728006817	c
0.15332568694671	14.35286469189308	4.91537629215721	c
6.89748468631037	32.25887020087987	6.14443089071952	c
10.17203190613031	28.71407520522878	5.73532580855201	c
2.86680216458586	34.64467993571106	7.35170837712213	c
-2.28643169974167	23.69856014685573	1.49049780925606	c
-6.50467007849834	25.13657972350003	3.19349948546214	c
-3.34013229524537	28.33003903563312	1.61308117946242	c
-0.32195086200423	31.98438428278459	18.46215324071117	c
4.11728523075454	33.01159248247230	20.06473668570461	c
8.88929263918840	33.76258417898578	20.03185360915155	c
10.86450412999681	24.38367906429737	23.81504095190529	c
15.06075661810988	22.91432442833861	22.07945503752745	c
11.86528947393156	19.74225573558647	23.64751473986612	c
0.56760692910595	13.84268854090623	20.68077852043709	c
-3.81225733620037	18.03265817055756	17.63818417235607	c
-1.49092079232049	22.07301147072555	18.58490984913088	c
-2.20133872980660	18.96849113992582	22.07404870651702	c
7.71402695551213	13.98563915171561	15.71817282303382	c
4.71845620432758	10.80225919334899	17.50886476170169	c
7.20583602420379	13.67659065046070	20.43300094546881	c
9.95456419233583	17.19695683046396	9.59867644762424	c

9.75007372071515	18.82212090801713	5.16742155341001	c
10.80074834753968	14.21302375546925	6.00242542767449	c
5.57358486389366	14.93361500451583	2.34257984036703	c
-0.76994228587821	16.98641527940283	4.04870553870716	c
0.14200113349828	12.49483036191666	2.65809044731708	c
-1.62930731766426	13.32004305695912	6.97912813763382	c
8.04849091591666	34.16023669549333	4.40779971211787	c
12.38897192770596	29.62163467000672	7.40554978652704	c
9.79730161747489	25.84852245451071	6.06751482233692	c
10.75488791286948	29.22292566857062	2.91719966300252	c
0.97338152331708	34.14912681120205	9.50414934545673	c
4.04791024378756	37.28537766259173	7.75527416865803	c
1.45271723792136	34.53433533389109	4.79780302883032	c
-2.34038510559249	21.85002882478776	2.48605123668524	h
-0.27051541704093	24.19436341630988	1.18689792764324	h
-3.20204383038330	23.44411214392731	-0.38673423421514	h
-7.37836081485307	25.06584698815323	1.27976639224902	h
-7.53035303319826	26.57780324163741	4.33088094486358	h
-6.79247643505978	23.26728388037871	4.11243681039588	h
-1.32747819549575	28.92554002909004	1.55804051836849	h
-4.45901575939265	29.85846978702486	2.52554973709180	h
-4.01898625877514	28.15546260120470	-0.36947597141529	h
-1.20868948529434	31.19262958753537	15.80249374956818	c
-0.92127800931363	29.81549657222756	20.32440723062291	c
-1.70399827702935	34.43416294249050	19.24976544226502	c
3.57253585091803	33.44962225074856	22.80727361425087	c
9.69471339048592	31.09972452689144	20.92540875797299	c
9.07985304129453	35.66408168355890	22.24447361519089	c
10.65238293470021	34.65487607746513	17.88750332622008	c
10.94699979171388	26.24319222520093	22.84166580679785	h
8.84110223517002	23.91220752040145	24.10802928307516	h
11.78039134598418	24.60255371150265	25.69649556605679	h
15.94682327756467	22.96076359790092	23.98819271996010	h
16.06316356862223	21.47059329325553	20.92444565982960	h

15.36314184627063	24.78744622234613	21.17266100121365	h
9.84511722082940	19.17275175599316	23.70434456850159	h
12.95942011968668	18.20776376450603	22.71549500677055	h
12.55547589610886	19.88873451806521	25.62835488430453	h
-1.08012419288900	11.98392969884015	19.71596618502494	c
1.21959297685668	13.81943857419006	23.26544549054617	c
-3.39242186751663	18.21455620809942	15.58602346011005	h
-4.01773718305041	15.99417880081970	18.09681352763679	h
-5.64913369052339	18.97937360478979	18.02979355925890	h
-3.28717663459106	23.04661180436430	19.07104867051273	h
0.07109000973908	22.98341395720045	19.65394503705118	h
-1.13917387233860	22.37178314886682	16.53354300924039	h
-2.61106413817453	16.98698587656745	22.61375903024122	h
-0.54426923025124	19.62678448092968	23.18373422150836	h
-3.87037271992426	20.13426442451659	22.59781877508918	h
6.78610148902926	13.72585183361830	13.84900939246750	h
8.45388624818698	15.94237646959076	15.82185671165980	h
9.33728391326475	12.65858226642411	15.83261951593362	h
6.28871475015815	9.41871591767939	17.30732733741509	h
3.54362429606794	10.20333490184103	19.13796542534971	h
3.54535554908941	10.72415774410195	15.76693061794407	h
7.94997278994194	15.61521942122535	20.72513728270085	h
5.91693465844414	13.23416674318987	22.02640336666003	h
8.80905230303716	12.31704955957923	20.49857831814975	h
8.74245553195188	18.78205273664546	10.26299855685465	h
9.71382528148574	15.55946522766775	10.89721290529731	h
11.95033886458056	17.83251910548598	9.65482234477753	h
11.74189184395614	19.44004264831636	5.42692542486657	h
9.43388112308890	18.37648710971361	3.14190892808737	h
8.48088976829715	20.40941186494687	5.69381257658955	h
10.33728907633103	12.52178828059827	7.16435328785523	h
10.44801545417422	13.75257063521993	3.98557437411833	h
12.85045871177415	14.63210370951399	6.22078366899520	h
6.30940585327859	12.48336740704440	1.57899152368423	c

5.44065267266203	16.88236004764205	0.53475082917635	c
-2.73024855083745	16.89207453086914	3.29140792043809	h
-0.72497026703269	18.32902474649147	5.66451963053092	h
0.48473232315386	17.76364201284266	2.55719090564261	h
1.19415575550006	13.25008851164289	1.01033931617933	h
1.00936963346245	10.66121497544725	3.20619914266271	h
-1.83805198319082	12.13605114239882	2.04670216369223	h
-3.57818559523144	13.10552307861412	6.22464044660818	h
-0.97361816500905	11.44089984619057	7.65872330071920	h
-1.71581804721260	14.62945160189378	8.62405008562275	h
9.85568822869999	35.88241803772141	5.33568340552860	c
7.35300090377192	34.22311726717928	1.83623975495858	c
11.99996860831114	29.20893264157968	9.43022104899219	h
12.67827866427053	31.69233839074187	7.19301746019639	h
14.17329108268972	28.65400182893758	6.85268467843820	h
11.51433055981293	24.80032526432422	5.47039205676495	h
8.17152970369958	25.17113007434489	4.92488242747305	h
9.42454973567120	25.35163531789926	8.07532695733245	h
11.39301901838257	31.18838106589714	2.57075152550075	h
9.05581399110170	28.85143450480385	1.74100310479719	h
12.29322912000030	27.93431751089887	2.29179570505636	h
1.92963262225418	34.32288502355033	11.36879729860899	h
0.16196039857103	32.22413979698592	9.34975433400158	h
-0.59967121380052	35.53858606548944	9.44571356492222	h
2.52390161251359	38.72151272618176	7.94439515634015	h
5.26966152676682	37.85467897995539	6.14957889698048	h
5.19505962278480	37.30637982705156	9.51659674945442	h
0.69128806726840	32.60872331431000	4.46202332970781	h
2.72612587813043	35.01353149709063	3.20279475280942	h
-0.14095819056771	35.90643506756096	4.78656007740094	h
-0.17864839199203	29.46017192544162	15.20059522492799	h
-0.85043732923648	32.71744257061402	14.39922536517003	h
-3.26863608421622	30.78162621231945	15.79631022875500	h
-2.96732020139844	29.33925013062873	20.23463046024071	h

-0.46828680864362	30.34834421091530	22.30105462693373	h
0.19183371460700	28.10316120232481	19.83540602040901	h
-1.17019127467989	36.01612246296238	17.97070030530049	h
-1.22931959733595	34.98128102601317	21.21927054018740	h
-3.78748539244040	34.16798029906484	19.13832860707975	h
2.99013654650136	35.93919318886954	23.57520753292522	c
3.70831839274778	31.51532949307821	24.63102932250751	c
11.69124244702054	31.08778522187164	21.58750994140189	h
9.48875479469040	29.72894745783550	19.34690799847713	h
8.46967929298537	30.43604693337272	22.49477347564780	h
8.05376040205555	35.00069819036894	23.94704267511291	h
8.28942723116762	37.52895235103651	21.68578473987227	h
11.09747541043295	35.93077928132775	22.77453481369943	h
12.63269049060945	34.78997625424820	18.57544232901898	h
10.06773111606105	36.54828811669754	17.18306618204328	h
10.62127655972771	33.30532497749763	16.27365207357831	h
-1.56703385682893	12.00227811890491	17.69160595852734	h
-2.03265499894640	10.08500979256535	21.30727624977294	c
2.45891482243300	15.31790132159453	24.00515706147744	h
0.27886486519937	11.90813222986562	24.84763463871060	c
6.39439593231094	10.96090098744035	2.99756167719245	h
6.90278724980889	11.98834928507064	-0.95624399139345	c
4.88389200073051	18.78845234856258	1.13528420196198	h
6.02736550253021	16.37655736319420	-2.00642132630321	c
10.36856094297046	35.83809773512602	7.35331282657847	h
10.96196159739806	37.65743121877257	3.70220137355058	c
5.98102637358973	32.82808806492383	1.12893107241024	h
8.45774419242730	36.00557346547593	0.21209033850773	c
2.91274003181459	37.45147896260031	22.14544710220677	h
2.54532795769796	36.48613250663561	26.13044827941612	c
4.14391993518079	29.57791345964682	24.02744152275406	h
3.27407446404820	32.07330123277751	27.19163667999858	c
-3.35683420532598	8.66458292717210	20.56497472826109	h
-1.32422779496494	10.02929576473026	23.86714706296796	c

0.80710844539921	11.88599851905588	26.86297780057844	h
7.47501775893446	10.06878069289998	-1.53109690631282	h
6.75690598088357	13.93692752764547	-2.75923928382431	c
5.91347979991070	17.91326177425960	-3.40911159303739	h
12.37464042014006	39.00133712475383	4.43667945510736	h
10.26282799264338	37.72279491471529	1.13913557960430	c
7.91436853244600	36.04476522399163	-1.79891717861164	h
2.09156513007919	38.43590080402439	26.70918208047926	h
2.69281592787214	34.55158020551155	27.94827871739205	c
3.39041629082955	30.54782554357473	28.60625603568341	h
-2.04614140040940	8.52117829384274	25.10909994807038	h
7.21541678064771	13.54953705610321	-4.75493282151575	h
11.13143420078538	39.11716622209522	-0.14278942682637	h
2.35311719161941	34.98044988614511	29.95915269220424	h
15.30670612838387	20.51818321563895	8.45854050341983	o
14.51833179778581	22.64013095219528	9.96679993470831	c
17.01129103269544	18.96189118730520	9.87408062313096	c
16.11440805768309	22.56376109363181	12.39374460860427	c
14.78212455132639	24.40998036140462	8.86132173513469	h
12.45748601546242	22.44909937397023	10.38048062471584	h
16.64610690136485	19.71743673376702	12.65069034313969	c
16.55456484100626	16.94588168152949	9.48077351085418	h
19.00302200515744	19.31312679165600	9.24775312692326	h
15.11274066824666	23.36975299488664	14.05109088685327	h
17.91339247837255	23.62164484880738	12.12785629895189	h
14.96397742506705	18.74696648522170	13.46009873887334	h
18.30561998557629	19.27381625307392	13.85766983867993	h
-7.84802936297579	10.37686913114650	22.11226191336592	o
-7.77963079667726	11.07099970478206	24.72134621692540	c
-8.10421274228510	12.60220033529733	20.58229722616030	c
-9.69516747771308	10.84729918299273	25.60054244482308	h
-6.45700222321791	9.77423880338957	25.71436046503621	h
-6.93509108228559	13.84109663518965	24.76758574108770	c
-8.28331628389572	14.86777176813629	22.40552836730915	c

-6.40690011393670	12.75580481907096	19.33785691576232	h
-9.79943190062633	12.41673599224811	19.34447418347631	h
-7.45327856566178	14.84621001762707	26.53639485469038	h
-4.84915113171540	13.94807890835089	24.52581726543126	h
-7.39014627955217	16.59917989285500	21.63034853630896	h
-10.29146569048069	15.31166632857762	22.84596564734548	h

1.74	0.00
5.96	100.00
10.28	28.47
13.63	20.12
17.66	1.74
19.78	30.84
21.80	9.29
22.92	11.62
25.76	14.61
26.45	2.92
26.86	5.78
28.42	8.85
31.15	1.10
31.41	2.28
32.09	1.66
33.31	4.22
34.01	6.20
34.92	2.20
38.31	4.12
39.54	4.93
41.99	3.00
42.06	1.46
42.84	4.70
43.68	5.15
44.34	2.70
44.57	1.74
47.35	1.06
48.24	0.86
49.30	1.76
50.05	1.18
50.86	1.89
52.61	0.97
53.22	2.10
54.35	2.99
56.20	4.17
56.63	3.07
58.24	4.48
59.13	1.80
61.34	0.88

61.91	0.94
63.03	8.89
66.05	0.86
66.25	5.13
67.83	7.42
69.56	0.44
72.44	0.48
72.90	0.52
73.96	1.49
76.79	1.05
77.35	2.59
78.19	1.09
82.81	1.78
84.21	4.11
87.11	0.38
88.82	1.06
91.27	1.64
92.58	0.83
95.63	0.50
97.32	0.26
98.80	0.40
101.10	0.31
102.15	0.85
104.86	0.20
106.15	0.58
106.53	0.24
108.30	0.52
109.64	0.89
111.19	0.60
111.53	0.98
113.40	0.52
116.52	0.79
118.84	0.19
122.72	1.21
124.10	1.22
125.37	0.93
127.10	1.19
128.56	0.32
131.76	0.61
133.95	1.21
135.37	0.35
136.20	0.51
139.76	0.37
141.09	0.08
142.81	0.08
143.30	0.05
145.83	0.09

151.08	0.11
151.91	0.27
152.71	0.92
157.91	2.98
160.68	1.77
164.67	1.43
168.79	0.05
169.53	0.11
171.73	0.04
172.50	0.16
173.19	0.17
178.62	0.88
181.71	0.05
182.48	0.62
183.84	0.17
186.18	0.43
206.18	0.12
207.29	0.08
222.82	0.11
223.02	0.07
223.56	0.01
226.62	0.26
230.04	0.11
230.27	0.11
231.06	0.12
232.84	0.11
233.47	0.14
234.49	0.18
236.02	0.06
243.17	0.06
243.69	0.37
246.32	0.17
247.86	0.26
251.05	0.13
254.58	0.13
255.68	0.06
256.12	0.08
256.60	0.46
256.80	0.10
259.45	0.13
261.02	0.03
262.96	0.26
264.63	0.18
264.94	0.53
266.16	0.43
266.49	0.31
267.96	0.20

270.04	0.14
270.33	0.01
273.18	0.12
277.93	0.16
278.82	0.01
279.76	0.41
283.36	0.05
286.00	0.24
287.23	0.04
288.93	0.01
293.18	0.05
293.74	0.04
294.08	0.02
297.29	0.03
298.24	0.02
304.95	0.03
307.90	0.04
308.43	0.07
309.93	0.13
310.77	0.17
311.17	0.23
314.97	0.07
315.96	0.08
318.46	0.07
319.11	0.13
320.23	0.11
321.87	0.16
322.13	0.37
322.87	0.05
323.27	0.11
324.56	0.18
329.89	0.39
333.79	0.02
337.31	0.03
344.32	0.04
355.08	0.04
355.41	0.04
361.42	0.09
363.48	0.12
367.94	0.08
369.00	0.07
372.53	0.01
374.25	0.13
375.23	0.02
378.51	0.05
380.37	0.08
380.79	0.01

383.85	0.04
385.16	0.09
390.57	0.11
391.34	0.09
391.99	0.01
392.14	0.13
397.51	0.07
398.37	0.13
399.16	0.13
399.64	0.15
406.61	0.03
408.23	0.05
411.53	0.01
413.34	0.01
443.56	0.03
444.18	0.05
446.24	0.03
446.69	0.06
457.89	0.01
459.25	0.03
463.67	0.02
464.24	0.02
468.55	0.13
470.67	0.15
471.83	0.10
472.24	0.03
477.43	0.10
478.08	0.03
479.44	0.02
480.27	0.18
480.57	0.10
481.44	0.07
490.71	0.29
491.00	0.12
502.22	0.11
502.48	0.01
502.63	0.08
503.40	0.05
526.07	0.05
527.50	0.25
529.81	0.99
530.96	0.50
557.08	0.12
557.27	0.38
558.49	0.56
560.23	0.21
565.90	0.07

566.79	0.08
572.07	0.07
573.71	0.01
573.96	0.06
574.17	0.02
577.14	0.09
577.78	0.11
578.00	0.13
578.58	0.09
594.27	0.04
595.42	0.05
598.32	0.06
601.53	0.08
606.77	0.07
607.44	0.08
608.64	0.05
608.79	0.05
663.90	0.01
665.49	0.02
666.07	0.01
666.42	0.02
675.52	0.08
676.75	0.06
677.09	0.03
678.21	0.03
697.09	0.43
697.70	1.26
706.79	0.08
707.02	0.01
707.13	0.04
708.93	0.04
709.03	0.03
710.06	0.02
734.34	0.46
736.25	0.46
736.64	0.04
737.17	0.01
748.12	0.01
748.93	0.28
748.99	0.06
749.43	0.02
783.58	0.04
784.14	0.03
784.45	0.05
784.79	0.06
785.85	0.01
790.19	0.03

802.45	0.03
802.56	0.00
806.95	0.05
807.35	0.09
809.29	0.04
810.48	0.03
810.55	0.01
811.87	0.04
817.86	0.01
819.23	0.03
832.09	0.02
836.79	0.03
842.70	0.02
843.11	0.01
845.44	0.01
847.12	0.03
847.36	0.01
849.25	0.03
869.51	0.02
870.53	0.01
885.36	0.05
889.82	0.01
891.94	0.05
892.98	0.06
893.88	0.03
894.55	0.02
895.01	0.05
895.28	0.04
896.12	0.02
897.38	0.02
899.10	0.02
902.33	0.02
904.66	0.04
905.33	0.05
905.68	0.05
906.02	0.04
906.74	0.04
907.54	0.02
909.16	0.08
911.54	0.04
912.77	0.02
913.36	0.02
913.42	0.04
913.68	0.02
913.92	0.01
914.97	0.03
916.66	0.09

917.00	0.09
918.10	0.05
918.54	0.06
918.90	0.06
923.46	0.02
924.31	0.00
925.83	0.02
927.01	0.00
928.52	0.00
928.99	0.03
929.27	0.03
936.04	0.00
937.05	0.00
937.75	0.01
938.20	0.00
940.45	0.00
942.13	0.00
942.25	0.00
942.51	0.01
945.50	0.00
953.77	0.02
954.08	0.00
956.99	0.01
961.56	0.01
967.69	0.01
968.84	0.00
970.78	0.00
984.23	0.01
985.75	0.58
986.54	0.48
986.55	1.11
986.79	0.65
989.04	0.12
990.69	0.07
994.15	0.04
1009.40	0.08
1009.60	0.00
1012.49	0.12
1014.01	0.33
1014.16	0.24
1014.36	0.40
1016.75	0.01
1016.86	0.01
1017.90	0.07
1018.69	0.04
1019.41	0.01
1019.49	0.08

1020.79	0.03
1020.85	0.01
1021.70	0.04
1022.72	0.02
1023.41	0.00
1023.86	0.08
1024.26	0.03
1027.20	0.28
1027.30	0.10
1027.66	0.27
1027.69	0.00
1028.98	0.07
1029.44	0.09
1030.56	0.03
1031.82	0.00
1036.24	0.02
1038.85	0.04
1040.53	0.02
1043.92	0.03
1046.52	0.01
1052.15	0.07
1055.77	0.06
1058.82	0.12
1059.23	0.14
1067.35	0.01
1073.92	0.00
1075.42	0.00
1076.07	0.00
1077.00	0.02
1077.05	0.03
1136.95	0.03
1138.25	0.04
1146.97	0.22
1147.66	0.27
1149.43	0.04
1149.60	0.04
1149.96	0.04
1151.60	0.01
1152.20	0.08
1153.87	0.04
1162.30	0.25
1162.46	0.12
1163.62	0.37
1164.28	0.01
1164.51	0.15
1165.17	0.38
1168.94	0.11

1170.78	0.05
1171.59	0.02
1171.69	0.05
1180.68	0.03
1181.20	0.02
1193.72	0.09
1193.99	0.15
1195.19	0.02
1195.70	0.04
1197.41	0.02
1200.13	0.02
1207.24	0.17
1207.56	0.28
1209.17	0.12
1209.36	0.05
1212.74	0.07
1213.70	0.07
1214.22	0.08
1217.05	0.04
1218.81	0.13
1220.41	0.07
1223.51	0.23
1223.77	0.21
1224.81	0.10
1226.26	0.05
1226.53	0.05
1226.87	0.02
1227.36	0.06
1228.20	0.12
1228.86	0.03
1229.08	0.11
1229.27	0.06
1230.67	0.05
1248.76	0.28
1251.42	0.27
1254.36	0.01
1254.94	0.18
1269.11	0.06
1270.39	0.01
1270.64	0.08
1272.67	0.00
1297.86	0.00
1298.65	0.00
1300.55	0.01
1301.23	0.01
1306.29	0.01
1306.96	0.02

1328.03	0.05
1331.11	0.04
1344.35	0.01
1344.64	0.01
1344.93	0.01
1345.05	0.01
1345.18	0.01
1345.79	0.01
1346.16	0.01
1346.55	0.01
1346.74	0.01
1346.86	0.01
1346.94	0.02
1347.49	0.01
1347.58	0.02
1347.68	0.01
1348.03	0.02
1348.50	0.02
1348.58	0.02
1350.34	0.01
1351.17	0.01
1353.94	0.01
1357.55	0.01
1357.84	0.01
1360.43	0.01
1361.37	0.01
1361.46	0.01
1361.53	0.00
1361.66	0.00
1362.20	0.01
1375.49	0.01
1375.99	0.07
1376.45	0.07
1376.90	0.01
1377.30	0.07
1378.17	0.04
1379.09	0.08
1381.75	0.02
1381.90	0.06
1382.93	0.06
1396.38	0.01
1397.03	0.02
1398.32	0.01
1399.41	0.00
1412.26	0.01
1413.48	0.01
1413.64	0.01

1413.74	0.02
1414.61	0.00
1415.43	0.01
1415.84	0.01
1416.70	0.15
1418.47	0.12
1420.44	0.04
1420.55	0.05
1420.80	0.02
1422.00	0.08
1423.78	0.06
1424.24	0.03
1424.70	0.11
1425.06	0.14
1425.14	0.03
1425.17	0.03
1425.29	0.09
1425.98	0.03
1426.83	0.04
1427.23	0.03
1427.39	0.03
1428.07	0.06
1428.35	0.05
1428.90	0.07
1430.46	0.03
1431.46	0.06
1431.78	0.05
1432.65	0.05
1433.58	0.05
1435.45	0.01
1436.14	0.07
1436.35	0.03
1436.76	0.04
1437.70	0.01
1438.44	0.11
1438.79	0.27
1439.16	0.19
1439.40	0.10
1439.72	0.11
1440.14	0.31
1440.98	0.18
1441.60	0.27
1442.09	0.09
1443.68	0.07
1444.60	0.03
1445.00	0.08
1445.12	0.09

1445.66	0.05
1446.27	0.02
1446.54	0.03
1447.48	0.09
1447.76	0.20
1447.89	0.18
1448.64	0.02
1455.16	0.11
1456.33	0.00
1456.79	0.00
1459.30	0.01
1459.85	0.20
1459.95	0.26
1460.31	0.11
1460.68	0.07
1461.20	0.08
1462.36	0.13
1463.03	0.28
1463.30	0.08
1465.30	0.17
1466.67	0.07
1467.10	0.27
1468.23	0.08
1468.36	0.24
1469.15	0.37
1471.78	0.16
1474.72	0.14
1475.52	0.16
1476.22	0.11
1480.22	0.06
1480.87	0.03
1484.87	0.02
1506.71	2.25
1508.33	2.09
1518.39	1.36
1520.88	1.12
1589.82	0.03
1590.03	0.07
1590.26	0.08
1590.67	0.04
1615.18	1.49
1616.14	1.33
1616.37	1.28
1617.94	1.38
1860.60	0.17
1861.44	0.02
1911.21	0.00

1915.57	0.08
2870.12	0.44
2882.64	0.31
2922.17	0.35
2925.14	0.22
2928.18	0.10
2928.36	0.17
2928.69	0.12
2931.86	0.12
2932.10	0.23
2932.57	0.22
2934.12	0.14
2936.69	0.12
2936.74	0.20
2937.97	0.15
2939.92	0.17
2940.22	0.15
2940.52	0.15
2940.60	0.03
2940.72	0.07
2941.14	0.19
2941.25	0.23
2944.82	0.17
2945.73	0.15
2945.78	0.21
2945.94	0.24
2946.08	0.51
2946.13	0.48
2946.82	0.48
2949.32	0.30
2952.00	0.19
2952.32	0.31
2953.51	0.35
2953.61	0.42
2953.71	0.46
2955.27	0.19
2958.15	0.26
2962.91	0.05
2970.22	0.27
2972.87	0.05
2977.03	0.07
2989.14	0.05
2995.01	0.06
3014.23	0.09
3014.38	0.08
3015.58	0.07
3017.30	0.06

3018.05	0.07
3018.25	0.11
3018.27	0.20
3019.57	0.07
3020.75	0.06
3021.19	0.07
3021.95	0.03
3022.58	0.01
3023.06	0.03
3023.20	0.03
3024.13	0.04
3024.36	0.02
3025.33	0.05
3025.80	0.04
3026.77	0.10
3026.96	0.08
3027.39	0.14
3027.63	0.08
3028.12	0.14
3028.64	0.13
3029.12	0.13
3029.27	0.09
3030.59	0.05
3031.26	0.06
3031.62	0.06
3032.44	0.05
3033.88	0.15
3034.44	0.15
3034.96	0.02
3035.38	0.07
3036.69	0.13
3038.31	0.08
3041.05	0.09
3041.49	0.01
3041.60	0.09
3042.69	0.09
3043.60	0.14
3044.30	0.02
3045.02	0.01
3045.03	0.03
3045.77	0.05
3046.40	0.10
3049.61	0.05
3049.63	0.04
3050.75	0.03
3052.81	0.02
3053.82	0.02

3054.81	0.05
3056.21	0.04
3056.86	0.04
3057.73	0.05
3058.14	0.04
3058.51	0.04
3060.71	0.04
3066.46	0.03
3068.11	0.03
3068.63	0.10
3070.36	0.07
3076.20	0.04
3076.28	0.05
3086.16	0.05
3086.68	0.05
3088.89	0.04
3092.92	0.06
3096.16	0.15
3096.62	0.15
3098.63	0.14
3103.04	0.04
3105.38	0.09
3105.76	0.09
3107.56	0.13
3109.67	0.23
3113.99	0.50
3114.37	0.54
3115.80	0.34
3124.85	0.14
3124.91	0.08
3136.46	0.22
3143.37	0.01
3143.60	0.03
3155.93	0.10
3159.32	0.04
3162.23	0.08
3163.97	0.05
3166.85	0.03
3170.12	0.03
3181.56	0.05
3188.95	0.04

I-P

-0.97139624756359	-4.65067218664742	-5.85208696004099	c
0.41181358247514	-5.83269941586372	-8.13169394443354	c

-3.85611609419420	-4.61220921078459	-6.30849302956526	c
-0.37678638284401	-6.19271584061335	-3.45299257222896	c
-0.08609971430160	-2.05150447245442	-5.33639515710912	n
-0.13515742525007	-4.93577804475443	-9.94690558875507	h
-0.06836068968906	-7.87547923460239	-8.25648999517767	h
2.49356954707110	-5.65770073763455	-7.90520143711600	h
-4.59046274563468	-6.56714985691696	-6.55632813181757	h
-4.32018638415752	-3.51911947252502	-8.04307227813377	h
-4.83455018791093	-3.73024244143512	-4.67006014354879	h
1.68432872708159	-6.17385201227430	-3.04992599907662	h
-1.00721121055526	-8.18239226048185	-3.69310454248620	h
-1.37512138548873	-5.37002503716023	-1.79749288351782	h
0.00000000000000	0.00000000000000	-6.83655024982397	c
0.00000000000000	-0.00000000000000	-2.38152970388514	si
0.00000000000000	0.00000000000000	-9.65048637109743	c
0.08609971430160	2.05150447245442	-5.33639515710912	n
3.40765705165420	0.00000000000000	0.00000000000000	p
-3.40765705165420	0.00000000000000	0.00000000000000	p
-2.25951740055231	0.45554084844250	-10.98593245609181	c
2.25951740055231	-0.45554084844250	-10.98593245609181	c
0.97139624756359	4.65067218664742	-5.85208696004099	c
0.00000000000000	0.00000000000000	2.38152970388514	si
-4.01234200280714	0.83679640180507	-9.92991445563627	h
-2.25759608286405	0.44515865031279	-13.63864224119339	c
2.25759608286405	-0.44515865031279	-13.63864224119339	c
4.01234200280714	-0.83679640180507	-9.92991445563627	h
3.85611609419420	4.61220921078459	-6.30849302956526	c
0.37678638284401	6.19271584061335	-3.45299257222896	c
-0.41181358247514	5.83269941586372	-8.13169394443354	c
-0.08609971430160	2.05150447245442	5.33639515710912	n
0.08609971430160	-2.05150447245442	5.33639515710912	n
-4.03050210711678	0.79588710520276	-14.67497348517078	h
0.00000000000000	0.00000000000000	-14.96849129939675	c
4.03050210711678	-0.79588710520276	-14.67497348517078	h

4.32018638415752	3.51911947252502	-8.04307227813377	h
4.83455018791093	3.73024244143512	-4.67006014354879	h
4.59046274563468	6.56714985691696	-6.55632813181757	h
1.00721121055526	8.18239226048185	-3.69310454248620	h
1.37512138548873	5.37002503716023	-1.79749288351782	h
-1.68432872708159	6.17385201227430	-3.04992599907662	h
-2.49356954707110	5.65770073763455	-7.90520143711600	h
0.13515742525007	4.93577804475443	-9.94690558875507	h
0.06836068968906	7.87547923460239	-8.25648999517767	h
0.00000000000000	0.00000000000000	6.83655024982397	c
-0.97139624756359	4.65067218664742	5.85208696004099	c
0.97139624756359	-4.65067218664742	5.85208696004099	c
0.00000000000000	0.00000000000000	-17.05191637173812	h
0.00000000000000	0.00000000000000	9.65048637109743	c
-0.37678638284401	6.19271584061335	3.45299257222896	c
0.41181358247514	5.83269941586372	8.13169394443354	c
-3.85611609419420	4.61220921078459	6.30849302956526	c
0.37678638284401	-6.19271584061335	3.45299257222896	c
-0.41181358247514	-5.83269941586372	8.13169394443354	c
3.85611609419420	-4.61220921078459	6.30849302956526	c
-2.25951740055231	-0.45554084844250	10.98593245609181	c
2.25951740055231	0.45554084844250	10.98593245609181	c
1.68432872708159	6.17385201227430	3.04992599907662	h
-1.00721121055526	8.18239226048185	3.69310454248620	h
-1.37512138548873	5.37002503716023	1.79749288351782	h
-0.13515742525007	4.93577804475443	9.94690558875507	h
-0.06836068968906	7.87547923460239	8.25648999517767	h
2.49356954707110	5.65770073763455	7.90520143711600	h
-4.83455018791093	3.73024244143512	4.67006014354879	h
-4.59046274563468	6.56714985691696	6.55632813181757	h
-4.32018638415752	3.51911947252502	8.04307227813377	h
-1.68432872708159	-6.17385201227430	3.04992599907662	h
1.00721121055526	-8.18239226048185	3.69310454248620	h
1.37512138548873	-5.37002503716023	1.79749288351782	h

0.13515742525007	-4.93577804475443	9.94690558875507	h
0.06836068968906	-7.87547923460239	8.25648999517767	h
-2.49356954707110	-5.65770073763455	7.90520143711600	h
4.83455018791093	-3.73024244143512	4.67006014354879	h
4.59046274563468	-6.56714985691696	6.55632813181757	h
4.32018638415752	-3.51911947252502	8.04307227813377	h
-4.01234200280714	-0.83679640180507	9.92991445563627	h
-2.25759608286405	-0.44515865031279	13.63864224119339	c
2.25759608286405	0.44515865031279	13.63864224119339	c
4.01234200280714	0.83679640180507	9.92991445563627	h
-4.03050210711678	-0.79588710520276	14.67497348517078	h
0.00000000000000	-0.00000000000000	14.96849129939675	c
4.03050210711678	0.79588710520276	14.67497348517078	h
0.00000000000000	-0.00000000000000	17.05191637173812	h

16.10	17.83
21.79	20.47
24.34	5.80
38.11	9.23
39.17	51.87
41.58	52.75
48.73	18.48
51.79	2.88
58.41	100.00
64.34	57.36
72.12	1.20
73.83	0.39
78.65	28.37
81.64	31.07
83.03	8.42
91.74	70.66
100.94	26.10
129.06	4.01
134.15	0.09
146.36	4.49
150.68	45.83
152.93	7.04
160.84	1.61
163.88	2.93
228.48	15.49
229.64	0.24
230.87	0.12
238.72	0.76

242.44	1.04
244.15	0.68
245.51	0.13
254.53	2.85
259.39	0.00
263.93	2.71
268.37	0.06
268.80	4.74
271.37	0.00
279.04	3.78
281.32	0.02
292.77	0.00
300.34	0.02
305.77	0.00
305.99	0.13
307.58	0.00
311.53	0.28
324.28	1.33
325.47	0.01
330.50	0.13
331.37	1.62
367.66	2.60
369.59	0.27
372.01	0.01
372.09	3.98
373.35	0.01
391.74	0.00
394.01	2.58
399.71	0.21
403.00	2.44
403.13	0.21
413.18	4.79
440.31	0.19
443.99	1.12
445.20	0.41
462.55	0.01
475.88	0.00
477.54	0.06
489.73	0.27
491.87	1.17
503.80	0.97
504.60	0.03
509.74	51.53
567.95	0.13
569.01	5.84
605.41	0.08
607.54	2.72

612.42	0.03
621.79	1.18
641.04	40.68
684.21	0.01
684.65	0.00
702.47	1.13
702.60	0.02
710.28	0.00
716.15	0.41
751.15	0.12
753.01	0.00
753.47	0.01
760.40	27.26
788.28	0.01
789.82	0.04
810.17	0.02
813.45	1.28
839.20	0.25
839.20	0.26
890.01	0.10
890.82	0.13
908.81	1.46
908.93	0.09
909.84	1.57
909.95	0.15
916.34	0.03
916.53	0.09
917.92	0.50
918.27	0.09
919.09	1.12
919.44	0.35
946.35	0.15
946.38	0.00
947.05	0.10
947.14	0.05
959.19	0.09
959.19	0.07
982.51	0.00
982.51	0.00
985.86	0.04
985.98	29.16
1015.57	0.06
1016.34	6.61
1025.60	3.37
1025.60	0.21
1025.88	0.01
1026.24	0.16

1028.54	0.24
1030.97	0.78
1032.66	0.06
1034.43	1.76
1069.11	0.04
1069.41	0.32
1075.23	0.05
1075.24	0.01
1150.98	0.91
1150.99	0.03
1164.27	0.12
1164.52	6.92
1170.64	0.01
1172.32	4.91
1199.15	0.08
1201.44	0.03
1214.71	0.13
1215.02	2.92
1217.29	0.51
1217.99	0.03
1233.23	0.00
1233.63	2.59
1233.86	0.04
1234.15	2.04
1266.77	0.06
1269.10	14.17
1294.77	0.10
1294.87	0.00
1346.09	0.09
1346.51	0.02
1346.91	0.02
1347.24	0.69
1348.52	0.07
1348.77	1.46
1354.05	0.00
1354.61	1.20
1362.25	0.00
1362.29	0.00
1377.43	0.41
1377.94	0.42
1382.16	0.04
1384.11	1.19
1411.97	0.11
1412.38	0.34
1413.00	0.37
1413.10	0.55
1424.31	4.49

1424.38	0.13
1424.90	0.03
1424.96	1.60
1428.59	2.48
1429.33	0.21
1434.18	1.86
1436.22	0.09
1437.11	0.30
1437.26	0.15
1441.20	0.17
1441.28	1.31
1441.51	4.09
1441.54	2.40
1444.87	0.62
1445.24	1.83
1445.84	0.23
1446.43	0.02
1461.92	0.71
1462.12	0.02
1463.01	0.21
1466.12	10.52
1469.00	0.06
1469.08	1.54
1469.60	0.20
1471.97	3.77
1522.60	0.21
1522.68	81.83
1591.29	1.57
1591.34	0.05
1618.11	0.04
1618.17	67.00
2936.20	0.20
2936.21	1.17
2936.24	0.26
2936.37	4.89
2942.41	0.11
2942.46	0.97
2943.85	0.05
2943.87	3.02
2948.09	0.90
2948.24	0.07
2948.32	0.03
2948.70	21.55
3026.85	0.27
3026.87	1.34
3026.94	0.02
3026.94	1.26

3029.77	0.69
3029.87	0.14
3030.01	0.07
3030.14	2.73
3033.75	1.49
3033.83	0.13
3033.93	0.06
3034.00	0.23
3035.98	0.57
3035.99	0.93
3037.29	0.19
3037.64	4.56
3040.56	0.18
3040.75	3.98
3040.93	0.48
3040.96	1.13
3051.73	0.81
3051.79	0.13
3051.96	0.01
3052.01	1.57
3091.51	0.05
3091.51	0.84
3100.44	4.15
3100.44	0.16
3108.95	0.02
3108.96	3.48
3115.66	0.66
3115.66	0.03
3120.84	0.20
3120.87	11.30

I-As

-0.70727063327673	-4.70485483975125	-5.94048093406333	c
0.82726707008883	-5.79871350426276	-8.16714582114756	c
-3.57117256955586	-4.87352302800470	-6.48757156623478	c
-0.08704643180357	-6.19208799729913	-3.51294246457681	c
-0.01822416720740	-2.05103211024854	-5.41953438008720	n
0.28203120463675	-4.94343478118574	-10.00277873758759	h
0.49026239732487	-7.86918220603589	-8.30054486937616	h
2.88381877169719	-5.48414469135984	-7.87181245724913	h
-4.15383246730622	-6.87645462801143	-6.75578835418892	h
-4.05995578497000	-3.81506256191623	-8.23626823770466	h

-4.66110314198066	-4.06735721060660	-4.88067815758774	h
1.95009428770514	-6.02051317503356	-3.03245721657564	h
-0.56277616605086	-8.22281098623545	-3.76533344019272	h
-1.20058007080173	-5.44154146883685	-1.89666792919550	h
0.00000000000000	0.00000000000000	-6.92260312889034	c
0.00000000000000	0.00000000000000	-2.46081456228952	si
0.00000000000000	0.00000000000000	-9.73686530585408	c
0.01822416720740	2.05103211024854	-5.41953438008720	n
3.60394759833601	0.00000000000000	0.00000000000000	as
-3.60394759833601	-0.00000000000000	0.00000000000000	as
-2.28338299870558	0.31277519324801	-11.07326574633547	c
2.28338299870558	-0.31277519324801	-11.07326574633547	c
0.70727063327673	4.70485483975125	-5.94048093406333	c
0.00000000000000	0.00000000000000	2.46081456228952	si
-4.05690638124458	0.58530070337791	-10.01793400067788	h
-2.28065051826923	0.30428692332582	-13.72608355425042	c
2.28065051826923	-0.30428692332582	-13.72608355425042	c
4.05690638124458	-0.58530070337791	-10.01793400067788	h
3.57117256955586	4.87352302800470	-6.48757156623478	c
0.08704643180357	6.19208799729913	-3.51294246457681	c
-0.82726707008883	5.79871350426276	-8.16714582114756	c
-0.01822416720740	2.05103211024854	5.41953438008720	n
0.01822416720740	-2.05103211024854	5.41953438008720	n
-4.07195398172663	0.54520605804010	-14.76212066334980	h
0.00000000000000	0.00000000000000	-15.05628395844304	c
4.07195398172663	-0.54520605804010	-14.76212066334980	h
4.05995578497000	3.81506256191623	-8.23626823770466	h
4.66110314198066	4.06735721060660	-4.88067815758774	h
4.15383246730622	6.87645462801143	-6.75578835418892	h
0.56277616605086	8.22281098623545	-3.76533344019272	h
1.20058007080173	5.44154146883685	-1.89666792919550	h
-1.95009428770514	6.02051317503356	-3.03245721657564	h
-2.88381877169719	5.48414469135984	-7.87181245724913	h
-0.28203120463675	4.94343478118574	-10.00277873758759	h

-0.49026239732487	7.86918220603589	-8.30054486937616	h
0.00000000000000	0.00000000000000	6.92260312889034	c
-0.70727063327673	4.70485483975125	5.94048093406333	c
0.70727063327673	-4.70485483975125	5.94048093406333	c
0.00000000000000	0.00000000000000	-17.13978084370686	h
0.00000000000000	0.00000000000000	9.73686530585408	c
-0.08704643180357	6.19208799729913	3.51294246457681	c
0.82726707008883	5.79871350426276	8.16714582114756	c
-3.57117256955586	4.87352302800470	6.48757156623478	c
0.08704643180357	-6.19208799729913	3.51294246457681	c
-0.82726707008883	-5.79871350426276	8.16714582114756	c
3.57117256955586	-4.87352302800470	6.48757156623478	c
-2.28338299870558	-0.31277519324801	11.07326574633547	c
2.28338299870558	0.31277519324801	11.07326574633547	c
1.95009428770514	6.02051317503356	3.03245721657564	h
-0.56277616605086	8.22281098623545	3.76533344019272	h
-1.20058007080173	5.44154146883685	1.89666792919550	h
0.28203120463675	4.94343478118574	10.00277873758759	h
0.49026239732487	7.86918220603589	8.30054486937616	h
2.88381877169719	5.48414469135984	7.87181245724913	h
-4.66110314198066	4.06735721060660	4.88067815758774	h
-4.15383246730622	6.87645462801143	6.75578835418892	h
-4.05995578497000	3.81506256191623	8.23626823770466	h
-1.95009428770514	-6.02051317503356	3.03245721657564	h
0.56277616605086	-8.22281098623545	3.76533344019272	h
1.20058007080173	-5.44154146883685	1.89666792919550	h
-0.28203120463675	-4.94343478118574	10.00277873758759	h
-0.49026239732487	-7.86918220603589	8.30054486937616	h
-2.88381877169719	-5.48414469135984	7.87181245724913	h
4.66110314198066	-4.06735721060660	4.88067815758774	h
4.15383246730622	-6.87645462801143	6.75578835418892	h
4.05995578497000	-3.81506256191623	8.23626823770466	h
-4.05690638124458	-0.58530070337791	10.01793400067788	h
-2.28065051826923	-0.30428692332582	13.72608355425042	c

2.28065051826923	0.30428692332582	13.72608355425042	c
4.05690638124458	0.58530070337791	10.01793400067788	h
-4.07195398172663	-0.54520605804010	14.76212066334980	h
0.00000000000000	0.00000000000000	15.05628395844304	c
4.07195398172663	0.54520605804010	14.76212066334980	h
0.00000000000000	-0.00000000000000	17.13978084370686	h

16.97	5.24
24.89	24.78
25.61	2.57
34.84	14.58
43.16	53.39
47.14	39.50
53.10	33.53
54.55	16.59
54.80	7.11
59.21	70.28
69.99	1.99
72.68	1.50
78.51	100.00
79.44	15.90
79.55	11.34
80.50	29.76
95.52	33.55
111.07	0.03
123.10	1.64
123.39	0.01
129.44	7.23
146.76	45.30
151.56	0.09
158.43	1.21
178.96	0.04
221.97	0.44
222.95	4.49
230.85	0.00
231.48	9.38
240.63	6.89
241.57	0.04
246.07	8.76
253.12	1.90
258.50	0.07
263.57	1.23
264.63	0.13
266.94	0.01
267.38	4.43
277.95	0.02

279.48	2.93
290.02	0.04
293.49	0.01
307.65	0.02
307.89	0.17
308.47	0.01
309.66	0.51
321.15	1.58
321.55	0.01
326.21	0.21
326.76	1.41
363.68	1.12
367.17	0.00
367.96	0.16
370.25	0.98
373.64	0.06
389.26	5.67
391.70	1.72
394.21	2.08
399.68	0.05
403.55	0.22
403.64	2.58
436.86	0.00
443.56	1.12
444.14	0.13
464.92	0.06
473.67	72.02
476.44	0.04
483.30	0.08
485.81	0.94
503.72	0.95
504.50	0.03
536.19	0.40
566.55	0.05
567.46	5.16
607.57	0.01
607.57	2.49
608.13	0.01
625.42	39.79
683.46	0.00
684.01	0.00
702.78	0.97
702.88	0.00
708.53	0.00
712.29	0.33
750.00	0.05
752.58	0.00

752.79	0.01
756.37	28.36
786.93	0.01
788.52	0.07
810.92	0.00
814.01	1.04
839.34	0.16
839.34	0.23
892.59	0.09
893.46	0.17
908.70	1.50
908.91	0.08
909.95	1.08
910.08	0.21
916.75	0.04
916.77	0.17
917.58	0.76
917.66	0.07
918.45	0.18
918.69	0.86
947.85	0.09
947.91	0.00
950.04	0.12
950.21	0.03
959.29	0.08
959.30	0.05
982.44	0.00
982.45	0.00
986.05	0.02
986.16	34.76
1016.05	0.01
1017.00	7.49
1026.47	0.37
1026.76	0.00
1027.28	2.33
1028.44	0.09
1028.70	0.04
1030.94	0.67
1033.37	0.08
1034.64	1.36
1069.36	0.02
1069.67	0.47
1075.34	0.03
1075.35	0.01
1150.96	0.56
1150.96	0.01
1164.82	0.06

1165.07	9.76
1170.76	0.00
1172.48	5.09
1200.28	0.12
1202.60	0.02
1213.61	0.15
1214.45	2.35
1216.86	0.53
1218.04	0.06
1232.85	0.01
1233.02	2.25
1233.44	0.04
1233.85	1.82
1269.24	0.02
1271.64	21.85
1295.28	0.03
1295.39	0.00
1348.35	0.03
1348.49	0.10
1348.50	0.03
1348.73	0.39
1349.60	0.03
1349.62	1.34
1355.03	0.01
1355.55	1.69
1361.96	0.00
1362.00	0.00
1378.13	0.59
1378.69	0.23
1382.56	0.03
1384.35	1.19
1412.90	0.05
1413.43	0.31
1413.80	0.19
1413.89	0.35
1424.14	3.28
1424.32	0.51
1425.38	0.04
1425.41	1.02
1427.97	2.20
1428.68	0.93
1432.64	0.70
1434.35	0.63
1436.88	0.28
1436.91	0.00
1442.22	1.03
1442.32	2.15

1442.35	0.01
1442.69	3.45
1444.48	0.71
1444.76	1.21
1445.39	0.10
1445.96	0.09
1461.37	0.62
1461.48	0.09
1463.11	0.09
1465.24	8.26
1466.92	0.12
1469.55	1.18
1470.13	0.11
1471.47	6.37
1522.79	0.11
1522.85	93.26
1591.02	2.69
1591.07	0.02
1617.77	0.01
1617.85	76.47
2936.72	0.20
2936.73	0.94
2936.76	0.20
2936.88	3.81
2942.28	0.08
2942.32	1.17
2943.80	0.03
2943.82	4.02
2948.66	0.84
2948.81	0.01
2948.83	0.01
2949.18	17.11
3026.93	0.18
3026.94	0.99
3027.05	0.04
3027.07	1.06
3030.16	0.51
3030.19	0.01
3030.50	0.01
3030.60	2.08
3034.16	0.47
3034.24	0.47
3034.88	0.01
3034.99	0.89
3035.49	1.39
3035.54	0.07
3036.35	0.24

3036.69	3.28
3040.87	0.12
3040.94	0.21
3040.98	0.79
3041.07	3.82
3052.19	0.66
3052.23	0.10
3052.41	0.01
3052.45	1.22
3091.27	0.02
3091.27	0.73
3100.17	3.38
3100.17	0.06
3108.43	0.01
3108.44	2.94
3115.20	0.71
3115.20	0.02
3120.54	0.08
3120.57	9.68

[Mo(CO)$_5$PMe$_3$]

0.03037019372558	3.09403573773638	0.00000000000000	mo
0.05895335013985	3.07833277525779	-3.87621373079853	c
-0.05670874431178	6.91078161248182	0.00000000000000	c
0.05895335013985	3.07833277525779	3.87621373079853	c
3.90740962959838	3.16073936698430	0.00000000000000	c
-3.84229809101455	2.96613416321735	0.00000000000000	c
0.08900306545746	3.07978469483752	-6.07363916089323	o
-0.11685396614584	9.10899416083245	0.00000000000000	o
0.08900306545746	3.07978469483752	6.07363916089323	o
6.10374724294140	3.20915787254824	0.00000000000000	o
-6.03909269832562	2.88588026341758	0.00000000000000	o
0.01391995238093	-1.73241063084172	0.00000000000000	p
3.12984050567889	-3.33282570431421	0.00000000000000	c
2.92078332691271	-5.42527182745943	0.00000000000000	h
4.21583308350814	-2.74670194142101	1.69856308158907	h
-1.59383645802629	-3.24266209551495	2.72226026287768	c
-1.59383645802629	-3.24266209551495	-2.72226026287768	c
-1.55921905328769	-5.33964131988126	2.57005696926361	h
-1.55921905328769	-5.33964131988126	-2.57005696926361	h

-3.58768894578283	-2.58856547610304	2.80327060248511	h
-0.64860371772872	-2.66315414447642	-4.50504047845555	h
4.21583308350814	-2.74670194142101	-1.69856308158907	h
-3.58768894578283	-2.58856547610304	-2.80327060248511	h
-0.64860371772872	-2.66315414447642	4.50504047845555	h

13.47	33.81
53.35	9.93
54.93	4.18
56.72	5.33
76.91	61.29
79.19	67.58
83.82	2.34
84.42	100.00
89.40	36.28
89.45	38.73
163.27	4.61
167.42	3.79
187.86	10.93
191.46	0.04
214.34	1.13
215.37	0.79
250.75	1.51
254.09	1.32
322.73	1.93
354.84	0.00
399.64	0.37
399.68	0.42
412.30	9.45
414.77	1.41
414.78	0.00
419.60	23.29
439.49	23.83
493.88	0.06
527.51	0.01
528.13	0.00
538.20	0.04
607.90	0.08
608.14	0.07
644.16	0.84
645.88	6.86
703.40	2.54
704.40	2.46
778.65	0.00
840.63	0.48
841.86	0.53

938.52	0.51
939.16	0.60
948.43	1.52
1268.42	0.25
1269.33	0.23
1291.71	0.16
1392.74	0.00
1400.90	2.19
1401.61	2.07
1409.58	1.65
1409.82	1.71
1420.26	0.85
1976.99	0.00
1977.34	0.06
1991.01	8.02
2003.25	12.09
2082.02	3.57
2949.36	3.89
2949.66	0.33
2949.79	8.20
3048.43	1.61
3048.68	0.35
3049.09	6.22
3062.14	1.19
3062.28	1.20
3062.39	0.18

VII. References

[1] O. J. Scherer, H. Sitzmann, G. Wolmershäuser, *J. Organomet. Chem.* **1984**, *268*, C9-C12.
[2] P. J. Sullivan, A. L. Rheingold, *Organometallics* **1982**, *1*, 1547-1549.
[3] L. Dütsch, C. Riesinger, G. Balázs, M. Scheer, *Chem. Eur. J.* **2021**, *27*, 8804-8810.
[4] J. R. Harper, A. L. Rheingold, *J. Organomet. Chem.* **1990**, *390*, c36-c38.
[5] S. S. Sen, A. Jana, H. W. Roesky, C. Schulzke, *Angew. Chem., Int. Ed.* **2009**, *48*, 8536-8538.
[6] S. Nagendran, S. S. Sen, H. W. Roesky, D. Koley, H. Grubmüller, A. Pal, R. Herbst-Irmer, *Organometallics* **2008**, *27*, 5459-5463.
[7] R. Köppe, H. Schnöckel, *Z. Anorg. Allg. Chem.* **2000**, *626*, 1095-1099.
[8] M. F. Guns, E. G. Claeys, G. P. Van Der Kelen, *J. Mol. Struct.* **1980**, *65*, 3-17.
[9] A. J. Kornath, A. Kaufmann, S. Cappellacci, *J. Mol. Spectrosc.* **2009**, *255*, 189-193.
[10] G. M. Sheldrick, *Acta Crystallogr. A* **2008**, *64*, 112-122.
[11] G. M. Sheldrick, *Acta Crystallogr. A* **2015**, *71*, 3-8.
[12] O. V. Dolomanov, L. J. Bourhis, R. J. Gildea, J. A. K. Howard, H. Puschmann, *J. Appl. Crystallogr.* **2009**, *42*, 339-341.
[13] R. Ahlrichs, M. Bär, M. Häser, H. Horn, C. Kölmel, *Chem. Phys. Lett.* **1989**, *162*, 165-169.
[14] A. D. Becke, *Phys. Rev. A: At. Mol. Opt. Phys.* **1988**, *38*, 3098-3100.
[15] J. P. Perdew, *Phys. Rev. B: Condens. Matter* **1986**, *33*, 8822-8824.
[16] J. P. Perdew, *Phys. Rev. B: Condens. Matter* **1986**, *34*, 7406-7406.
[17] S. Grimme, J. Antony, S. Ehrlich, H. Krieg, *J. Chem. Phys.* **2010**, *132*, 154104.
[18] O. Treutler, R. Ahlrichs, *J. Chem. Phys.* **1995**, *102*, 346-354.
[19] P. Deglmann, K. May, F. Furche, R. Ahlrichs, *Chem. Phys. Lett.* **2004**, *384*, 103-107.
[20] D. Rappoport, F. Furche, *J. Chem. Phys.* **2007**, *126*, 201104.
[21] Y. Tao, W. Zou, S. Nanayakkara, E. Kraka, *J. Chem. Theory Comput.* **2022**, *18*, 1821-1837.
[22] W. Zou, Y. Tao, M. Freindorf, D. Cremer, E. Kraka, *Chem. Phys. Lett.* **2020**, *748*, 137337.
[23] E. B. Wilson, *The Theory of Infrared and Raman Vibrational Spectra. McGraw-Hill, New York* **1955**.
[24] J. J. Turner, J. A. Timney, *J. Mol. Spectrosc.* **2022**, *387*, 111662.
[25] J. Baker, P. Pulay, *J. Am. Chem. Soc.* **2006**, *128*, 11324-11325.
[26] T. Lu, F. Chen, *J. Comput. Chem.* **2012**, *33*, 580-592.
[27] T. Lu, *J. Chem. Phys.* **2024**, *161*, 082503.
